# Efficacy and Safety of Oral Non‐Statin Lipid‐Lowering Therapies in Dyslipidaemia: A Systematic Review and Network Meta‐Analysis

**DOI:** 10.1002/edm2.70353

**Published:** 2026-09-30

**Authors:** Tamer Hodrob, Reem J. Saad, Aya Hamdy, Mariam Saleh Alheneedy, Ahmed Mohamed Shehata Abdelfattah Elsayed, Elsaghir Ghazy, Hazem Ayesh

**Affiliations:** ^1^ Faculty of Medicine Al‐Quds University Abu Dis Palestine; ^2^ College of Biotechnology Misr University for Science and Technology Giza Egypt; ^3^ Faculty of Medicine Ain Shams University Cairo Egypt; ^4^ Faculty of Medicine Zagazig University Zagazig Egypt; ^5^ Faculty of Medicine University of Tripoli Tripoli Libya; ^6^ Deaconess Health System Evansville Indiana USA

**Keywords:** combination lipid‐lowering therapy, dyslipidaemia, ezetimibe, LDL‐C, network meta‐analysis, obicetrapib

## Abstract

**Introduction:**

Dyslipidaemia remains a major contributor to atherosclerotic cardiovascular disease (ASCVD), and many patients fail to achieve recommended low‐density lipoprotein cholesterol (LDL‐C) targets despite statin therapy or are unable to tolerate statins. Oral non‐statin therapies have emerged as important alternatives; however, their comparative efficacy and safety remain unclear.

**Methods:**

We systematically searched PubMed, Embase, Web of Science, and Cochrane Central up to December 2025 for clinical trials evaluating these medications in adults with dyslipidaemia. Primary outcomes were percent and absolute changes in LDL‐C, while secondary outcomes included other lipid parameters and safety profile. A frequentist random‐effects network meta‐analysis was performed.

**Results:**

Forty‐three trials comprising 17,021 participants were included. Combination therapies showed the greatest efficacy, with obicetrapib 10 mg plus ezetimibe 10 mg achieving the largest reduction in percent LDL‐C (−49.15%; 95% CI: −59.42 to −38.89), followed by bempedoic acid plus ezetimibe (−36.90%; 95% CI: −44.65 to −29.15). Obicetrapib also showed substantial improvements in HDL‐C and ApoB. Safety outcomes were generally reassuring, with no significant increase in serious adverse events, myalgia, headache, or elevated liver enzymes; however, treatment discontinuation was higher with bempedoic acid 180 mg and colesevelam 3.75 g. The certainty of evidence was low to very low for many efficacy outcomes, primarily because of heterogeneity and imprecision.

**Conclusions:**

Oral non‐statin combination therapies, particularly obicetrapib plus ezetimibe and bempedoic acid plus ezetimibe, provide the most effective LDL‐C lowering and are valuable for patients with statin intolerance or residual risk. Ezetimibe remains a well‐tolerated option. Long‐term cardiovascular outcome trials are needed to confirm clinical benefit.

## Graphical abstract

A network meta‐analysis of 43 randomized trials showed that oral combination therapies, particularly obicetrapib plus ezetimibe and bempedoic acid plus ezetimibe, achieved the greatest LDL‐C reduction with reassuring safety profiles, supporting their use as effective oral alternatives for patients with dyslipidaemia.

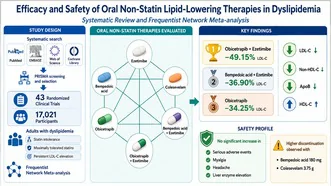

## 1 Introduction

Dyslipidaemia refers to an abnormal blood lipid profile characterized by elevated levels of low‐density lipoprotein cholesterol (LDL‐C), total cholesterol (TC), and triglycerides (TG), coupled with reduced high‐density lipoprotein cholesterol (HDL‐C) [1, 2]. It arises through primary (genetic or familial) or secondary (e.g., due to obesity, diabetes mellitus, or unhealthy lifestyles) mechanisms [1]. Dyslipidaemia remains highly prevalent worldwide, affecting approximately 39% of adults globally, with prevalence continuing to rise in recent decades [3, 4, 5]. Elevated LDL‐C has emerged as a major global risk factor for mortality, rising from the 15th leading risk factor in 1990 to the 8th in 2019 [1]. This trend supports its causal role in atherosclerotic cardiovascular disease (ASCVD), the leading cause of death worldwide, responsible for approximately 17.9 million deaths (32% of all deaths) in 2019 [1, 6, 7, 8]. Consequently, LDL‐C reduction has become a cornerstone strategy for the prevention and management of ASCVD [9].

Statins are the most commonly prescribed LDL‐C lowering agents, they hinder cholesterol biosynthesis by inhibiting 3‐βhydroxy 3β‐methylglutaryl Coenzyme A (HMG‐CoA) reductase and subsequentially reducing the risk of CVD in susceptible individuals [10, 11]. Nevertheless, many patients fail to achieve recommended LDL‐C targets, while 5%–30% report statin intolerance. These two issues may overlap, because patients who discontinue or reduce statin therapy because of intolerance may also fail to reach optimal LDL‐C control [12]. Thus, oral non‐statin therapies have emerged to address residual cardiovascular risk and unmet clinical needs [13, 14]. Among these, ezetimibe has been proven to significantly enhance lipid‐lowering effects when combined with statin or other oral non‐statin therapies compared to monotherapy [15, 16]. Furthermore, oral agents such as bempedoic acid, colesevelam hydrochloride and obicetrapib have shown promising efficacy with favourable safety and tolerability profiles [17, 18, 19].

Despite decades of clinical use of ezetimibe since 2002 and bempedoic acid since 2020 [20, 21]. Definitive comparative evidence regarding the efficacy and safety of oral non‐statin therapies in statin‐intolerant or maximally tolerated patients remains limited. Moreover, no comprehensive network meta‐analysis has directly compared multiple oral non‐statin lipid‐lowering agents across statin‐intolerant patients, individuals receiving maximally tolerated statin therapy, or those failing to achieve LDL‐C targets despite statin use. To address this gap, we conducted a network meta‐analysis evaluating both monotherapy and combination regimens, focusing on key efficacy outcomes, including different lipid parameters, as well as safety outcomes. This analysis aims to support evidence‐based clinical decision‐making and improve dyslipidaemia management.

## 2 Methods

### 2.1 Study Design and Registration

The study protocol was prospectively registered on the Open Science Framework (OSF; Registration. DOI: https://osf.io/6xunv/overview) [22]. This NMA was conducted in accordance with PRISMA‐NMA guidelines [23]. The PRISMA checklist was followed throughout the study (Supporting Infomation S12).

### 2.2 Search Strategy

We systematically searched PubMed, Embase, Web of Science, and the Cochrane Central Register of Controlled Trials to identify relevant studies from inception to 28 December 2025. The search terms included: (hypercholesterolemia OR hypercholesterolaemia OR dyslipidemia OR dyslipidaemia OR “mixed dyslipidemia” OR “mixed dyslipidaemia” OR “elevated LDL” OR “high LDL”) AND (ezetimibe OR bempedoic acid OR “bempedoic acid ezetimibe” OR Nexlizet OR Nustendi OR obicetrapib OR “TA‐8995” OR colesevelam) AND (placebo OR “background therapy” OR “standard therapy” OR comparator) AND (trial) AND (LDL OR “LDL cholesterol” OR “LDL‐C” OR “low‐density lipoprotein”). The detailed search strategy is reported in Supporting Infomation S1. No language or publication status restrictions were applied during the literature search. However, all studies that met the eligibility criteria were published in English; therefore, no language‐specific data extraction procedures were required.

### 2.3 Screening Process

Three reviewers independently reviewed and screened titles and abstracts, followed by full‐text reviews to specify the eligible studies. Any discrepancies were resolved by consensus or by a fourth reviewer when needed.

### 2.4 Study Selection

We included clinical trials evaluating oral non‐statin lipid‐lowering therapies (ezetimibe, bempedoic acid, bempedoic acid–ezetimibe, obicetrapib, and colesevelam) versus placebo or background statin therapy in adults with dyslipidaemia who were statin‐intolerant, receiving maximally tolerated statins, or not achieving LDL‐C targets despite statin use. Eligible studies required ≥ 8 weeks of follow‐up and reported either percentage or absolute changes in LDL‐C, while reporting of additional lipid outcomes and adverse events was preferred but not mandatory. Paediatric‐only studies, familial hypercholesterolemia, injectable or non‐pharmacological interventions, non‐comparative studies, and conference abstracts without full texts were excluded.

### 2.5 Data Extraction

Two independent reviewers manually extracted data using a standardized form. Extracted variables encompassed study characteristics (sample size, intervention details, follow‐up duration) (Supporting Infomation S4), participant demographics (age, sex, weight, BMI), and outcome measures, including percent and absolute changes in LDL‐C, HDL‐C, non‐HDL‐C, triglycerides, total cholesterol, ApoA, ApoB, as well as adverse event profiles. Combined means and standard deviations were calculated following the Cochrane guidelines [24]. Outcome measures were recorded in mg/dL and standardized, where necessary, to ensure comparability across studies.

### 2.6 Statistical Analysis

We conducted a frequentist random‐effects network meta‐analysis using the *netmeta* package in R [25], integrating data across trials to enable direct and indirect comparisons of multiple therapies. Mean differences (MDs) were calculated for continuous outcomes and relative risks (RRs) for dichotomous outcomes, each with 95% confidence intervals. The comparator was either background statin therapy or placebo, depending on the study design. In studies evaluating add‐on therapy, participants remained on maximally tolerated statin therapy throughout the trial. In placebo‐controlled trials, participants had discontinued statin therapy before randomization following the protocol‐specified washout period, and placebo served as the comparator. When multiple doses of the same intervention were evaluated, only the highest tolerated dose was included in the primary analysis to provide a consistent comparison of each agent under its most efficacious tested regimen and to avoid duplication of similar treatment nodes. Treatment rankings were estimated using *P*‐scores (ranging from 0 to 1), with higher values indicating greater relative effectiveness among the included interventions. Heterogeneity across studies was assessed using tau‐squared (*τ* ^2^), which measures between‐study variance; *I*‐squared (*I* ^2^), which estimates the proportion of total variability due to heterogeneity; and *Q* statistics, which test for the presence of heterogeneity. Regarding thresholds, we interpreted *I* ^2^ values as follows: 0%–25% indicating minimal heterogeneity, 25%–50% indicating moderate heterogeneity and above 50% indicating high heterogeneity [26]. Global inconsistency was examined using design‐by‐treatment interaction models to ensure validity of the NMA.

The outcomes evaluated included percent and absolute changes in LDL‐C, HDL‐C, non‐HDL‐C, TG, TC, ApoA, and ApoB. Detailed absolute outcome data are presented in Supporting Infomation S2 and S3. Safety outcomes included serious adverse events, treatment discontinuation due to adverse events, and the frequency of headache, myalgia, and elevated liver enzymes. Sensitivity analyses were conducted using a leave‐one‐out approach to assess the influence of individual trials on overall network estimates (Supporting Infomation S11). Publication bias was assessed using funnel plots and Egger's test for the primary outcomes.

### 2.7 Bias Assessment and Certainty of Evidence

The Cochrane Risk of Bias 2 (RoB 2) tool was used to assess the risk of bias for each outcome across five domains: randomization process, deviations from intended interventions, missing outcome data, measurement of the outcome and selection of the reported result. For each domain, the risk of bias was classified as low risk, some concerns, or high risk (Supporting Infomation S6) [27]. Two authors independently performed the assessments, with discrepancies resolved through discussion or consultation with a third reviewer. The certainty of evidence was evaluated using the Confidence in Network Meta‐Analysis (CINeMA) framework (Supporting Infomation S10) [28].

## 3 Results

### 3.1 Study Characteristics

A total of 2354 records were identified through database searches, of which 213 full‐text articles were assessed for eligibility. Ultimately, 43 studies met the inclusion criteria, comprising 17,021 participants (see Figure 1). The included trials evaluated bempedoic acid, ezetimibe, obicetrapib, colesevelam, and their combinations. The comparator was either background statin therapy or placebo. Study duration ranged from 8 to 52 weeks. The mean age of participants was 62.02 years (SD 10.69), with a mean body mass index (BMI) of 29.48 kg/m^2^ (SD 6.51) and a mean baseline LDL‐C of 134.69 mg/dL (SD 48.24). Baseline characteristics of participants and trials are summarized in Tables S4 and S5.

**FIGURE 1:**
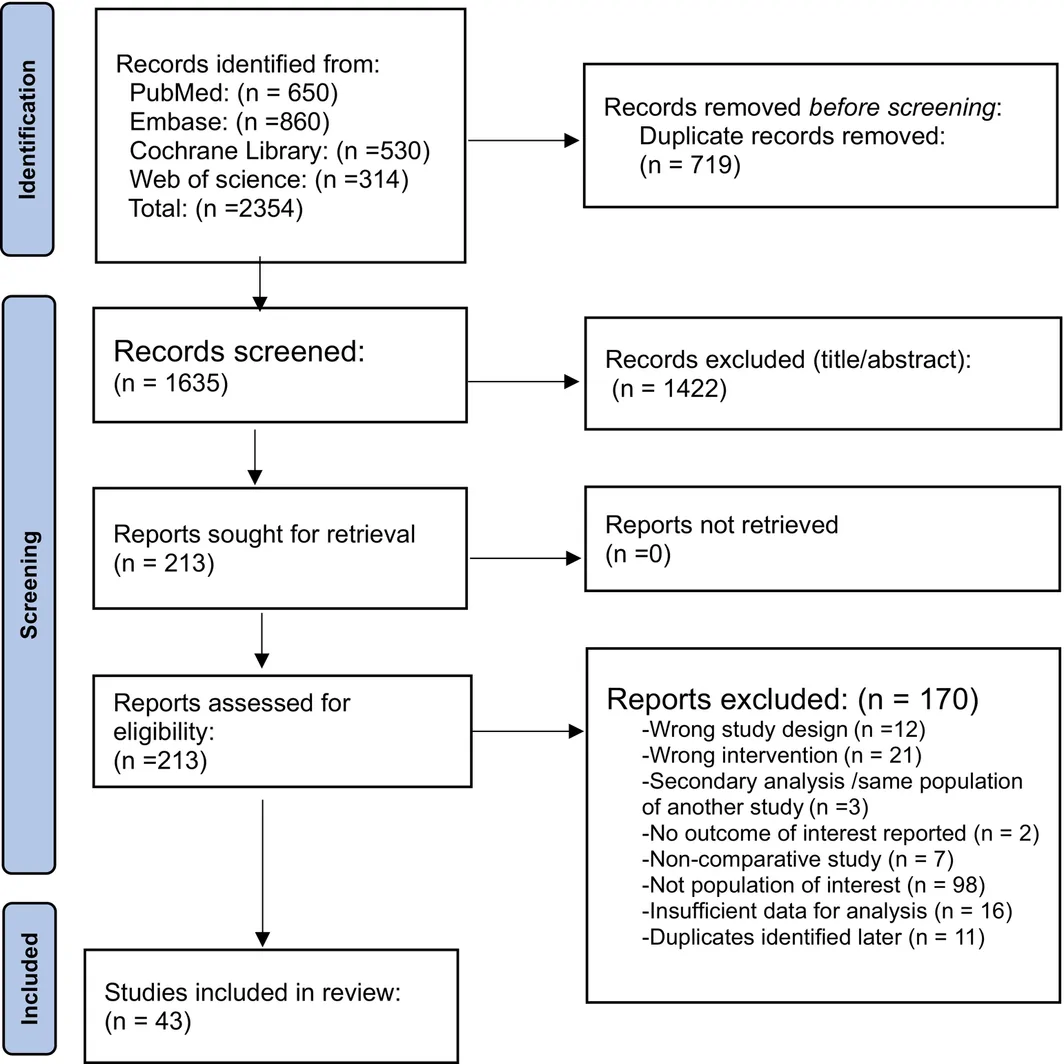
PRISMA flowchart for study selection.

### 3.2 Efficacy Outcome

#### 3.2.1 Percent LDL‐C

In the random‐effects model, all evaluated oral non‐statin therapies significantly reduced percentage LDL‐C levels compared with the comparator. The greatest reduction was observed with obicetrapib 10 mg plus ezetimibe 10 mg (MD: −49.15%; 95% CI: −59.42 to −38.89), followed by bempedoic acid 180 mg plus ezetimibe 10 mg (MD: −36.90%; 95% CI: −44.65 to −29.15) and obicetrapib 10 mg monotherapy (MD: −34.25%; 95% CI: −40.53 to −27.97) (Figures 2A and 3A). The heterogeneity analysis revealed high heterogeneity with an *I* ^2^ of 90.1% and *τ* ^2^ = 41.56. Tests of heterogeneity within designs were significant (*Q* = 314.71, df = 31, *p* < 0.0001), indicating substantial variability among the study results. Global inconsistency testing under the full design‐by‐treatment interaction model did not indicate incoherence (*Q* = 0.00, df = 0), supporting the validity of the indirect comparisons. The baseline covariates were examined using univariable meta‐regression, which identified baseline age (*p* < 0.048), baseline white (*p* < 0.001), male (*p* = 0.005), triglycerides (*p* = 0.033), and CAD (*p* = 0.0321) as significant effect modifiers, suggesting that part of the observed heterogeneity is explained by between‐trial differences in these baseline characteristics. Sensitivity analyses were conducted to further explore the robustness of the findings. Leave‐one‐out analysis confirmed the stability of the pooled estimates and treatment rankings (see Table S11.1). The certainty of evidence was generally low‐very low due to concerns of heterogeneity, imprecision, within‐study bias, and reporting bias (see Table S10.1). Assessment of publication bias using Egger's test demonstrated significant funnel plot asymmetry (test statistic = −7.9504, *p* = 0), suggesting a high concern for publication bias (see Figure S7.1). Treatment rankings were based on *P*‐scores, with higher values indicating better relative treatment performance. Obicetrapib 10 mg plus ezetimibe 10 mg ranked highest (*P*‐score = 0.9941), followed by bempedoic acid 180 mg plus ezetimibe 10 mg (*P*‐score = 0.7943), whereas placebo ranked lowest. The complete treatment ranking and league table for percentage change in LDL‐C are presented in Tables S8.1 and S9.1.

**FIGURE 2:**
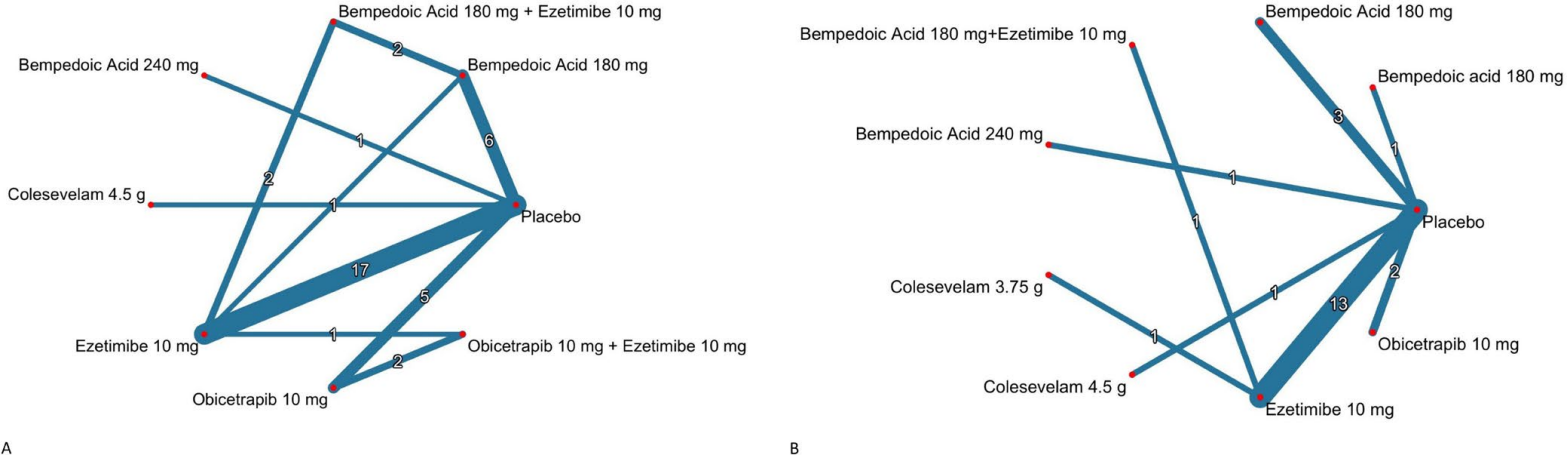
Network plot of treatment comparisons for percent change (A) and absolute change (B) in LDL‐C. Each circle indicates a treatment node. Lines connecting two nodes represent direct comparisons between two treatments; the thickness of the lines is proportional to the number of trials directly comparing the two connected treatments.

**FIGURE 3:**
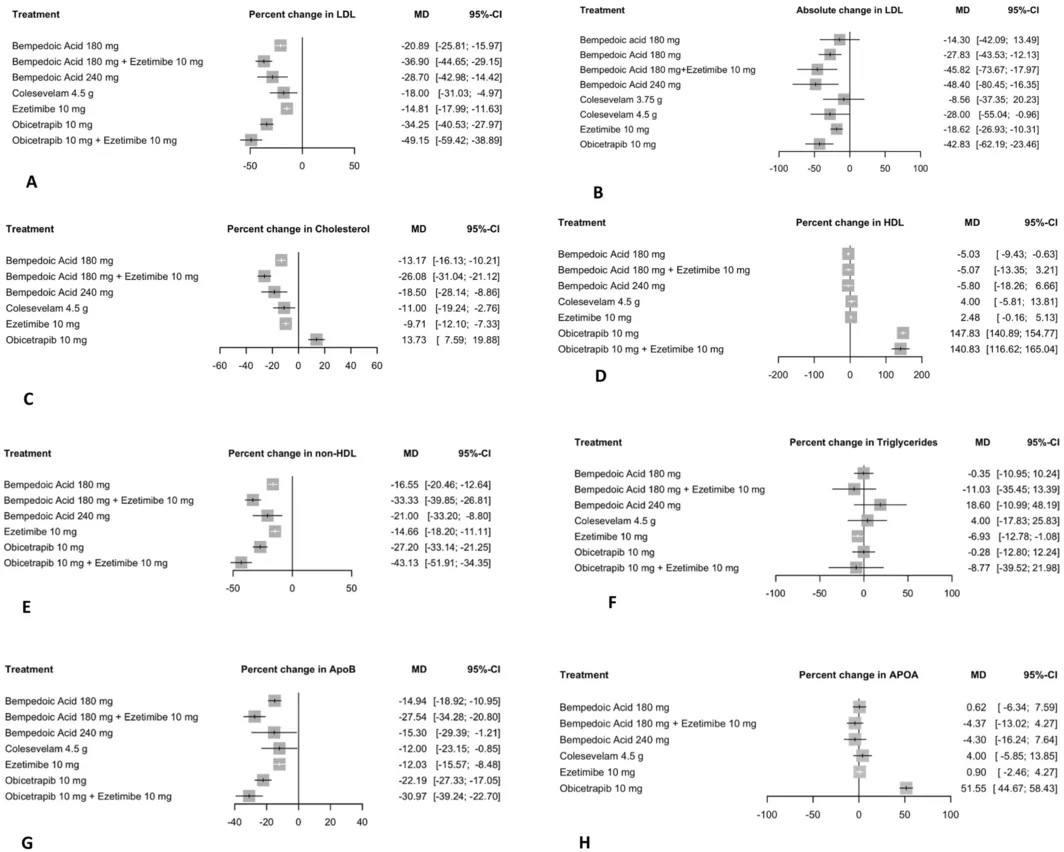
Meta‐analysis networks result for (A) percent change in LDL‐C, (B) absolute change in LDL‐C, (C) percent change in TC, (D) percent change in HDL‐C, (E) percent change in non‐HDL‐C, (F) percent change in TG, (G) percent change in APO B, and (H) percent change in APO A. Effect sizes are presented as mean difference (MD) and 95% confidence intervals (CI).

#### 3.2.2 Absolute LDL‐C

In the random‐effects model assessing absolute change in LDL‐C, bempedoic acid 240 mg demonstrated the greatest absolute reduction in LDL‐C compared with the comparator (MD: −48.40 mg/dL; 95% CI: −80.45 to −16.35), followed by bempedoic acid 180 mg plus ezetimibe 10 mg (MD: −45.82 mg/dL; 95% CI: −73.67 to −17.97) and obicetrapib 10 mg (MD: −42.83 mg/dL; 95% CI: −62.19 to −23.46). In contrast, colesevelam 3.75 g did not show a statistically significant reduction in LDL‐C (MD: −8.56 mg/dL; 95% CI: −37.35 to 20.23) (Figures 2B and 3B). The heterogeneity analysis revealed high heterogeneity with an *I* ^2^ of 92.2% and *τ* ^2^ = 182.91. Tests of heterogeneity within designs were significant (*Q* = 192.86, df = 15, *p* < 0.0001), indicating substantial variability among the study results. Global inconsistency testing under the full design‐by‐treatment interaction model did not indicate incoherence (*Q* = 0.00, df = 0), supporting the validity of the indirect comparisons. The baseline covariates were examined using univariable meta‐regression, which identified white (*p* < 0.001) as significant effect modifiers, suggesting that part of the observed heterogeneity is explained by between‐trial differences in these baseline characteristics. Sensitivity analyses were conducted to further explore the robustness of the findings. Leave‐one‐out analysis confirmed the stability of the pooled estimates and treatment rankings (see Table S11.2). The certainty of evidence was generally low—very low due to concerns of heterogeneity, imprecision, within‐study bias, and reporting bias (see Table S10.2). Assessment of publication bias using Egger's test demonstrated significant funnel plot asymmetry (test statistic = −4.2148, *p* = < 0.00001), suggesting a high concern for publication bias (see Figure S7.2). Treatment rankings were based on *P*‐scores, with higher values indicating better relative treatment performance. Bempedoic acid 240 mg ranked as the most effective intervention (*P*‐score = 0.8412), followed by bempedoic acid 180 mg plus ezetimibe 10 mg (0.8241), while placebo ranked lowest. Complete treatment rankings and league tables are presented in Tables S8.2 and S9.2.

#### 3.2.3 Percent Total Cholesterol

In the random‐effects model assessing percentage change in total cholesterol, all evaluated oral non‐statin therapies except obicetrapib 10 mg showed significant reductions compared with comparators. The greatest reduction was observed with bempedoic acid 180 mg plus ezetimibe 10 mg (MD: −26.08%; 95% CI: −31.04 to −21.12), followed by bempedoic acid 240 mg (MD: −18.50%; 95% CI: −28.14 to −8.86) and bempedoic acid 180 mg (MD: −13.17%; 95% CI: −16.13 to −10.21). In contrast, obicetrapib 10 mg was associated with a significant increase in total cholesterol (MD: 13.73%; 95% CI: 7.59–19.88) (Figure 3C). This finding is likely attributable to its pronounced HDL‐C–raising effect rather than an increase in atherogenic lipoproteins. The heterogeneity analysis revealed high heterogeneity with an *I* ^2^ of 88.0% and *τ* ^2^ = 15.61. Tests of heterogeneity within designs were significant (*Q* = 182.88, df = 22, *p* < 0.0001), indicating substantial variability among the study results. Global inconsistency testing under the full design‐by‐treatment interaction model did not indicate incoherence (*Q* = 0.00, df = 0), supporting the validity of the indirect comparisons. The baseline covariates were examined using univariable meta‐regression, which identified male (*p* = 0.0159), cholesterol (*p* = 0.0257), and study duration (*p* = 0.0269) as significant effect modifiers, suggesting that part of the observed heterogeneity is explained by between‐trial differences in these baseline characteristics. Sensitivity analyses were conducted to further explore the robustness of the findings. Leave‐one‐out analysis confirmed the stability of the pooled estimates and treatment rankings (see Table S11.3). The certainty of evidence was generally low—very low due to concerns of heterogeneity, imprecision, within‐study bias, and reporting bias (see Table S10.3). Assessment of publication bias using Egger's test demonstrated significant funnel plot asymmetry (test statistic = −3.8513, *p* = < 0.0001), suggesting a high concern for publication bias (see Figure S7.3). Treatment rankings were based on *P*‐scores, with higher values indicating better relative treatment performance. Bempedoic acid 180 mg plus ezetimibe 10 mg ranked as the most effective intervention (*P*‐score = 0.9856), followed by bempedoic acid 240 mg (0.7951), while obicetrapib 10 mg ranked lowest. Network plot, complete treatment rankings and league tables are presented in Figure S3.3 and Tables S8.3 and S9.3, respectively.

#### 3.2.4 Percent HDL‐C

In the random‐effects model assessing percentage change in HDL‐C, obicetrapib‐containing regimens showed the greatest increases compared with comparators. Obicetrapib 10 mg monotherapy demonstrated the highest increase (MD: 147.83%; 95% CI: 140.89–154.77), followed by obicetrapib 10 mg plus ezetimibe 10 mg (MD: 140.83%; 95% CI: 116.62–165.04). In contrast, bempedoic acid 180 mg was associated with a modest reduction in HDL‐C, while other bempedoic acid‐based therapies, colesevelam 4.5 g, and ezetimibe 10 mg showed no significant changes (Figure 3D). The heterogeneity analysis revealed high heterogeneity with an *I* ^2^ of 88.6% and *τ* ^2^ = 20.8. Tests of heterogeneity within designs were significant (*Q* = 176, df = 20, *p* < 0.0001), indicating substantial variability among the study results. Global inconsistency testing under the full design‐by‐treatment interaction model did not indicate incoherence (*Q* = 0.00, df = 0), supporting the validity of the indirect comparisons. The baseline covariates were examined using univariable meta‐regression, which identified male (*p* = 0.00002), white (*p* = 0.0082), and cholesterol (*p* = 0.0027) as significant effect modifiers, suggesting that part of the observed heterogeneity is explained by between‐trial differences in these baseline characteristics. Sensitivity analyses were conducted to further explore the robustness of the findings. Leave‐one‐out analysis confirmed the stability of the pooled estimates and treatment rankings (see Table S11.5). The certainty of evidence was generally low—very low due to concerns of heterogeneity, imprecision, within‐study bias, and reporting bias (see Table S10.5). Assessment of publication bias using Egger's test demonstrated significant funnel plot asymmetry (test statistic = 2.7276, *p* = 0.0115), suggesting a high concern for publication bias (see Figure S7.5). Treatment rankings were based on *P*‐scores, with higher values indicating better relative treatment performance. Bempedoic acid 180 mg ranked as the most effective intervention (*P*‐score = 0.8410), followed by bempedoic acid 180 mg plus ezetimibe 10 mg (0.8191), while obicetrapib 10 mg ranked lowest. Network plot, complete treatment rankings and league tables are presented in Figure S3.5 and Tables S8.5 and S9.5, respectively.

#### 3.2.5 Percent Non‐HDL‐C

In the random‐effects model assessing percentage change in non‐HDL‐C, all evaluated oral non‐statin therapies showed significant reductions compared with comparators. The greatest reduction was observed with obicetrapib 10 mg plus ezetimibe 10 mg (MD: −43.13%; 95% CI: −51.91 to −34.35), followed by bempedoic acid 180 mg plus ezetimibe 10 mg (MD: −33.33%; 95% CI: −39.85 to −26.81) and obicetrapib 10 mg monotherapy (MD: −27.20%; 95% CI: −33.14 to −21.25), while ezetimibe 10 mg showed the smallest reduction (MD: −14.66%; 95% CI: −18.20 to −11.11) (Figure 3E). The heterogeneity analysis revealed high heterogeneity with an *I* ^2^ of 88.6% and *τ* ^2^ = 27.23. Tests of heterogeneity within designs were significant (*Q* = 193.53, df = 22, *p* < 0.0001), indicating substantial variability among the study results. Global inconsistency testing under the full design‐by‐treatment interaction model did not indicate incoherence (*Q* = 0.00, df = 0), supporting the validity of the indirect comparisons. The baseline covariates were examined using univariable meta‐regression, which identified no associations between treatment effects on percent non‐HDL‐C and baseline covariates. Sensitivity analyses were conducted to further explore the robustness of the findings. Leave‐one‐out analysis confirmed the stability of the pooled estimates and treatment rankings (see Table S11.7). The certainty of evidence was generally low—very low due to concerns of heterogeneity, imprecision, within‐study bias, and reporting bias (see Table S10.7). Assessment of publication bias using Egger's test demonstrated significant funnel plot asymmetry (test statistic = −10.1254, *p* = 0.0), suggesting a high concern for publication bias (see Figure S7.7). Treatment rankings were based on *P*‐scores, with higher values indicating better relative treatment performance. Obicetrapib 10 mg plus ezetimibe 10 mg ranked as the most effective intervention (*P*‐score = 0.9938), followed by bempedoic acid 180 mg plus ezetimibe 10 mg (0.8186), while placebo ranked lowest. Network plot, complete treatment rankings and league tables are presented in Figure S3.7 and Tables S8.7 and S9.7, respectively.

#### 3.2.6 Percent TG

In the random‐effects model assessing percentage change in triglycerides, only ezetimibe 10 mg showed a significant reduction compared with comparators (MD: −6.93%; 95% CI: −12.78 to −1.08), whereas all other evaluated oral non‐statin therapies showed nonsignificant effects (Figure 3F). The heterogeneity analysis revealed high heterogeneity with an *I* ^2^ of 88.6% and *τ* ^2^ = 100.299. Tests of heterogeneity within designs were significant (*Q* = 175.18, df = 18, *p* < 0.0001), indicating substantial variability among the study results. Global inconsistency testing under the full design‐by‐treatment interaction model did not indicate incoherence (*Q* = 0.02, df = 2, *p* = 0.9877), supporting the validity of the indirect comparisons. The baseline covariates were examined using univariable meta‐regression, which identified no associations between treatment effects on percent TG and baseline covariates. Sensitivity analyses were conducted to further explore the robustness of the findings. Leave‐one‐out analysis confirmed the stability of the pooled estimates and treatment rankings (see Table S11.9). The certainty of evidence was generally low—very low due to concerns of heterogeneity, imprecision, within‐study bias, and reporting bias (see Table S10.9). Assessment of publication bias using Egger's test showed no significant funnel plot asymmetry (test statistic = −1.3042, *p* = 0.204), suggesting no concern for publication bias (see Figure S7.9). Treatment rankings were based on *P*‐scores, with higher values indicating better relative treatment performance. Bempedoic acid 180 mg plus ezetimibe 10 mg ranked as the most effective intervention (*P*‐score = 0.7602), followed closely by ezetimibe 10 mg (0.7557), whereas bempedoic acid 240 mg ranked lowest. Network plot, complete treatment rankings and league tables are presented in Figure S3.9 and Tables S8.9 and S9.9, respectively.

#### 3.2.7 Percent ApoB

In the random‐effects model assessing percentage change in ApoB, all evaluated oral non‐statin therapies showed significant reductions compared with comparators. The greatest reduction was observed with obicetrapib 10 mg plus ezetimibe 10 mg (MD: −30.97%; 95% CI: −39.24 to −22.70), followed by bempedoic acid 180 mg plus ezetimibe 10 mg (MD: −27.54%; 95% CI: −34.28 to −20.80) and obicetrapib 10 mg monotherapy (MD: −22.19%; 95% CI: −27.33 to −17.05), while bempedoic acid monotherapy, colesevelam 4.5 g, and ezetimibe 10 mg showed smaller but significant reductions (Figure 3G). The heterogeneity analysis revealed high heterogeneity with an *I* ^2^ of 88.6% and *τ* ^2^ = 29.18. Tests of heterogeneity within designs were significant (*Q* = 202.39, df = 23, *p* < 0.0001), indicating substantial variability among the study results. Global inconsistency testing under the full design‐by‐treatment interaction model did not indicate incoherence (*Q* = 0.0, df = 0), supporting the validity of the indirect comparisons. The baseline covariates were examined using univariable meta‐regression, which identified male (*p* = 0.0058), white (*p* = < 0.001), and triglycerides (*p* = 0.0121) as significant effect modifiers, suggesting that part of the observed heterogeneity is explained by between‐trial differences in these baseline characteristics. Leave‐one‐out analysis confirmed the stability of the pooled estimates and treatment rankings (see Table S11.11). The certainty of evidence was generally low—very low due to concerns of heterogeneity, imprecision, within‐study bias, and reporting bias (see Table S10.11). Assessment of publication bias using Egger's test demonstrated significant funnel plot asymmetry (test statistic = −7.554, *p* = 0.0), suggesting a high concern for publication bias (see Figure S7.11). Treatment rankings were based on *P*‐scores, with higher values indicating better relative treatment performance. Obicetrapib 10 mg plus ezetimibe 10 mg ranked as the most effective intervention (*P*‐score = 0.9565), followed by bempedoic acid 180 mg plus ezetimibe 10 mg (0.8687), while placebo ranked lowest. Network plot, complete treatment rankings and league tables are presented in Figure S3.11 and Tables S8.11 and S9.11, respectively.

#### 3.2.8 ApoA Percent Outcome

In the random‐effects model assessing percentage change in ApoA, most evaluated oral non‐statin therapies showed no significant effects compared with comparators. Obicetrapib 10 mg was the only therapy associated with a significant increase in ApoA levels (MD: 51.55%; 95% CI: 44.67–58.43), whereas bempedoic acid‐based therapies, colesevelam 4.5 g, and ezetimibe 10 mg showed no significant changes (Figure 3H). The heterogeneity analysis revealed high heterogeneity with an *I* ^2^ of 92.1% and *τ* ^2^ = 22.65. Tests of heterogeneity within designs were significant (*Q* = 126.37, df = 10, *p* < 0.0001), indicating substantial variability among the study results. Global inconsistency testing under the full design‐by‐treatment interaction model did not indicate incoherence (*Q* = 0.0, df = 0), supporting the validity of the indirect comparisons. The baseline covariates were examined using univariable meta‐regression, which identified male (*p* = < 0.001), white (*p* = 0.0022), triglycerides (*p* = 0.0104), cholesterol (*p* = < 0.001) and study duration (*p* = 0.0350) as significant effect modifiers, suggesting that part of the observed heterogeneity is explained by between‐trial differences in these baseline characteristics. Leave‐one‐out analysis confirmed the stability of the pooled estimates and treatment rankings (see Table S11.13). The certainty of evidence was generally low—very low due to concerns of heterogeneity, imprecision, within‐study bias, and reporting bias (see Table S10.13). Assessment of publication bias using Egger's test showed no significant funnel plot asymmetry (test statistic = −0.1943, *p* = 0.84), suggesting no concern for publication bias (see Figure S7.13). Treatment rankings were based on *P*‐scores, with higher values indicating better relative treatment performance. Bempedoic acid 180 mg plus ezetimibe 10 mg ranked as the most effective intervention (*P*‐score = 0.8363), followed by bempedoic acid 240 mg (0.7769), while obicetrapib 10 mg ranked lowest. Network plot, complete treatment rankings and league tables are presented in Figure S3.13 and Tables S8.13 and S9.13, respectively.

Across the remaining absolute lipid outcomes, bempedoic acid 240 mg consistently showed the greatest reductions in total cholesterol and non‐HDL‐C compared with comparator, whereas obicetrapib 10 mg showed the greatest increase in HDL‐C and the largest reduction in ApoB. In contrast, colesevelam 3.75 g generally showed the smallest improvements among active treatments. Heterogeneity ranged from low for HDL‐C (*I* ^2^ = 16.9%) to high for LDL‐C (*I* ^2^ = 92.2%) and total cholesterol (*I* ^2^ = 89.0%), while moderate heterogeneity was observed for non‐HDL‐C (*I* ^2^ = 45.7%) and ApoB (*I* ^2^ = 61.4%). Treatment rankings were generally consistent with the estimated treatment effects across outcomes. Detailed effect estimates are provided in Supporting Infomation S2. Complete treatment rankings are presented in Tables S9.4, S9.6, S9.8, S9.10 and S9.12. The corresponding network plots and funnel plots are presented in Figures S3.4, S3.6, S3.8, S3.10 and S3.12 and Figures S7.4, S7.6, S7.8, S7.10 and S7.12, respectively. League tables and CINeMA assessments are provided in Tables S8.4, S8.6, S8.8, S8.10 and S8.12 and Tables S10.4, S10.6, S10.8, S10.10 and S10.12, respectively. Leave‐one‐out analysis are provided in Tables S11.4, S11.6, S11.8, S11.10 and S11.12.

### 3.3 Safety Outcomes

#### 3.3.1 Serious Adverse Events (SAEs)

A total of 34 studies reported SAEs. In the random‐effects model, none of the evaluated oral non‐statin therapies showed a statistically significant increase in SAE risk compared with comparators (Figure 4A). Heterogeneity was negligible (*I* ^2^ = 0%), and sensitivity analysis confirmed robustness (see Table S11.14). The *P*‐scores, which rank treatments based on their effectiveness with higher scores indicating more favourable safety profile were highest for obicetrapib 10 mg plus ezetimibe 10 mg (*P*‐score = 0.84), followed by obicetrapib 10 mg and placebo. Complete treatment ranking table is presented in Table S9.14. The network plot, funnel plot, league table and CINeMA are provided in Figures S3.14 and S7.14 and Tables S8.14 and S10.14, respectively.

**FIGURE 4:**
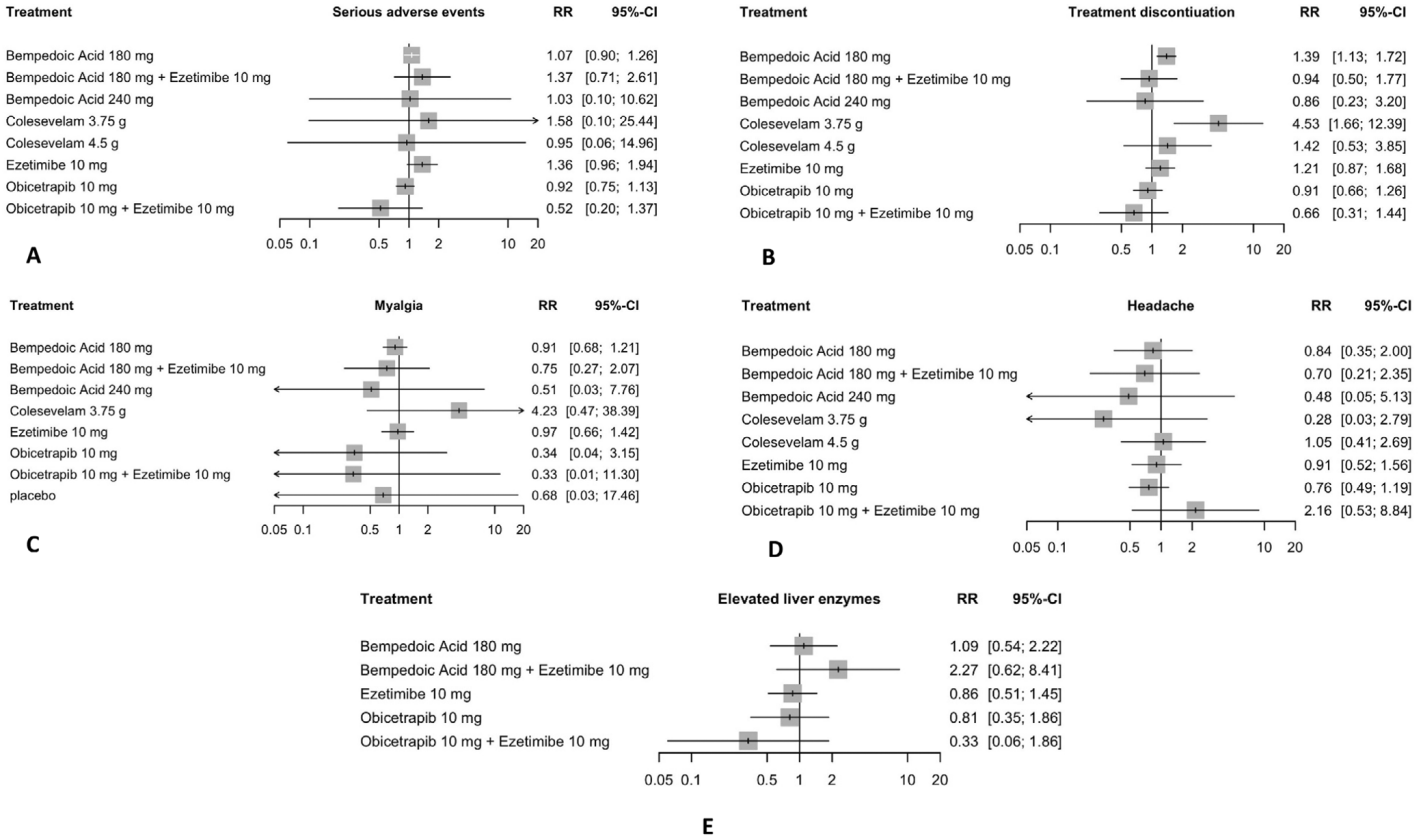
Network meta‐analysis results for safety outcomes. (A) Serious adverse events, (B) treatment discontinuation due to adverse events, (C) myalgia, (D) headache, and (E) elevated liver enzymes. Effect sizes are presented as risk ratio (RR) and 95% confidence intervals (CI).

#### 3.3.2 Treatment Discontinuation

In the random‐effects model assessing treatment discontinuation due to adverse events, most evaluated oral non‐statin therapies showed no statistically significant difference in discontinuation risk versus comparators. However, bempedoic acid 180 mg (RR: 1.39; 95% CI: 1.13–1.72) and colesevelam 3.75 g (RR: 4.53; 95% CI: 1.66–12.39) were associated with significantly increased risks (Figure 4B). Heterogeneity was negligible (*I* ^2^ = 0%), and sensitivity analysis confirmed robustness (see Table S11.15). The *P*‐scores, which rank treatments based on their effectiveness with higher scores indicating more favourable outcomes were highest for obicetrapib 10 mg plus ezetimibe 10 mg (*P*‐score = 0.85), followed by obicetrapib 10 mg (0.69) and bempedoic acid 240 mg (0.65). Complete treatment ranking table is presented in Table S9.15. The network plot, funnel plot, league table and CINeMA are provided in Figures S3.15 and S7.15 and Tables S8.15 and S10.15, respectively.

#### 3.3.3 Myalgia

In the random‐effects model assessing myalgia risk, none of the evaluated oral non‐statin therapies showed a statistically significant increase in myalgia risk versus comparators (Figure 4C). Heterogeneity was negligible (*I* ^2^ = 0%), and sensitivity analysis confirmed robustness (see Table S11.16). The *P*‐scores, which rank treatments based on their effectiveness with higher scores indicating more favourable outcomes, were highest for obicetrapib 10 mg ranked (*P*‐score = 0.74), followed by obicetrapib 10 mg plus ezetimibe 10 mg (0.68) and bempedoic acid 240 mg (0.61). Complete treatment ranking table is presented in Table S9.16. The network plot, funnel plot, league table and CINeMA are provided in Figures S3.16 and S7.16 and Tables S8.16 and S10.16, respectively.

#### 3.3.4 Headache

In the random‐effects model assessing headache risk, no statistically significant differences were observed among the evaluated oral non‐statin therapies compared with comparators (Figure 4D). Heterogeneity was negligible (*I* ^2^ = 0%), and sensitivity analysis confirmed robustness (see Table S11.17). The *P*‐scores, which rank treatments based on their effectiveness with higher scores indicating more favourable outcomes, were highest for colesevelam 3.75 g (*P*‐score = 0.81), followed by bempedoic acid 240 mg (0.66) and obicetrapib 10 mg (0.60). Complete treatment ranking table is presented in Table S9.17. The network plot, funnel plot, league table and CINeMA are provided in Figures S3.17 and S7.17 and Tables S8.17 and S10.17, respectively.

#### 3.3.5 Elevated Liver Enzymes

In the random‐effects model assessing elevated liver enzyme risk, no statistically significant differences were observed among the evaluated oral non‐statin therapies compared with comparators (Figure 4E). Heterogeneity was negligible (*I* ^2^ = 0%), and sensitivity analysis confirmed robustness (see Table S11.18). The *P*‐scores, which rank treatments based on their effectiveness with higher scores indicating more favourable outcomes, were highest for obicetrapib 10 mg plus ezetimibe 10 mg (*P*‐score = 0.89), followed by obicetrapib 10 mg (0.60) and ezetimibe 10 mg (0.58). Complete treatment ranking table is presented in Table S9.18. The network plot, funnel plot, league table and CINeMA are provided in Figures S3.18 and S7.18 and Tables S8.18 and S10.18, respectively.

## 4 Discussion

This network meta‐analysis provides a comprehensive comparison of oral non‐statin lipid‐lowering therapies in patients with dyslipidaemia who are statin‐intolerant, receiving maximally tolerated statins, or not achieving LDL‐C targets. Combination therapies, particularly obicetrapib plus ezetimibe, showed the greatest reductions in percent LDL‐C and non–HDL‐C, with pooled MDs of −49.15% (95% CI: −59.42 to −38.89) and −43.13% (95% CI: −51.91 to −34.35), respectively. These results are clinically meaningful, as LDL‐C remains the primary therapeutic target and residual cardiovascular risk persists in patients who do not achieve guideline‐recommended levels or cannot tolerate statins. Notably, obicetrapib also produced marked effects on HDL‐C and ApoB; however, the cardiovascular implications of these findings should be interpreted cautiously in light of previous experience with cholesteryl ester transfer protein (CETP) inhibitors. Although earlier CETP inhibitors, including torcetrapib, dalcetrapib, evacetrapib, and anacetrapib, favourably increases in HDL‐C and, in some cases, reduced LDL‐C, yet failed to demonstrate clear cardiovascular outcome benefits, and torcetrapib was discontinued because of serious adverse events [29, 30, 31, 32, 33]. Accordingly, these findings indicate that the marked HDL‐C increases observed with obicetrapib should be interpreted cautiously. Greater clinical relevance should therefore be placed on its effects on LDL‐C, non‐HDL‐C, and ApoB, which are more directly linked to atherogenic risk and cardiovascular risk reduction.

Safety analyses findings were reassuring overall. SAEs, myalgia, headache, and elevated liver enzymes were not significantly increased with evaluated therapies, and heterogeneity was low for these outcomes. However, treatment discontinuation due to adverse events was higher with bempedoic acid 180 mg and colesevelam 3.75 g, suggesting differences in tolerability despite acceptable overall safety profiles. These findings support individualized treatment selection, particularly in patients at higher risk of medication discontinuation.

The findings of our network meta‐analysis are consistent with recent systematic reviews and network meta‐analyses [34, 35, 36], which identified obicetrapib 10 mg as the most effective agent for reducing percentage LDL‐C. These findings are further supported by the recent GRADE‐assessed meta‐analysis by Emara et al., which demonstrated that obicetrapib consistently produces marked reductions in LDL‐C and non‐HDL‐C while improving achievement of lipid parameters irrespective of ASCVD risk [37]. Our analysis extends this evidence by showing that combination therapy with obicetrapib and ezetimibe achieves even greater reductions in LDL‐C and non‐HDL‐C than obicetrapib monotherapy. Consistent with previous studies, obicetrapib also produced the largest increase in HDL‐C. Overall, these findings reinforce obicetrapib as a highly potent oral lipid‐lowering therapy; however, its ultimate clinical value will depend on confirmation of cardiovascular benefit in dedicated outcome trials.

The observed superiority of obicetrapib may be attributed to its unique mechanism as a cholesteryl ester transfer protein (CETP) inhibitor. By inhibiting the transfer of cholesteryl esters from HDL to apolipoprotein B‐containing lipoproteins, obicetrapib produces substantial reductions in LDL‐C and non‐HDL‐C while markedly increasing HDL‐C (see Table 1) [38]. However, despite these favourable lipid effects, definitive cardiovascular outcome data are still lacking. Phase 3 trials, including broadway and brookyn, have showed robust LDL‐C lowering [39, 40], while the ongoing PREVAIL trial will determine whether these improvements translate into reductions in major adverse cardiovascular events. In contrast, bempedoic acid lowers LDL‐C by inhibiting ATP‐citrate lyase and has already demonstrated cardiovascular benefit in the CLEAR trial, particularly in statin‐intolerant patients [41]. Thus, although obicetrapib appears to provide greater lipid‐lowering efficacy, confirmation of long‐term cardiovascular benefit is still required before it can be considered an established therapy for routine clinical practice.

**TABLE 1:** Summary of mechanisms, efficacy, advantages and limitations of oral non‐statin regimens.

Therapy	Mechanism of action	Expected LDL‐C reduction	Advantages	Limitations
Ezetimibe	Inhibits the Niemann‐Pick C1‐Like 1 (NPC1L1) transporter, reducing intestinal cholesterol absorption	Modest	Well tolerated, oral, inexpensive and widely used	Limited LDL‐C lowering as monotherapy
Bempedoic acid	Inhibits ATP‐citrate lyase upstream of HMG‐CoA reductase, upregulating hepatic LDL receptors	Moderate	Useful in statin intolerance, proven CV outcome data	May increase uric acid, and discontinuation in some patients
Bempedoic acid + Ezetimibe	Dual hepatic cholesterol synthesis and intestinal absorption inhibition	Greater than either agent alone	Greater LDL‐C reduction than monotherapy	Compounded tolerability/discontinuation risks driven primarily by the bempedoic acid component
Colesevelam	Bile acid sequestrant	Modest	Systemically non‐absorbed agent Useful in specific populations where systemic agents are avoided (e.g., pregnancy)	GI side effects, and pill burden
Obicetrapib	CETP inhibition	Strong	Very potent LDL‐C and ApoB lowering, also raises HDL‐C	CV outcome benefit not yet established, HDL‐C effect uncertain
Obicetrapib + Ezetimibe	CETP + NPC1L1 inhibition	Greater than either agent alone	Highest efficacy among evaluated oral regimens Nearing the potency of injectable monoclonal antibodies	Long‐term outcome efficacy data are still pending

To place our findings in the context of current lipid‐lowering strategies, it is important to consider currently available injectable therapies. PCSK9 inhibitors, including alirocumab and evolocumab, typically reduce LDL‐C by approximately 50%–60% and have demonstrated significant reductions in major adverse cardiovascular events [42, 43]. Similarly, inclisiran achieves LDL‐C reductions of approximately 50% [44]. In our network meta‐analysis, obicetrapib plus ezetimibe reduced LDL‐C by 49.15%, approaching the efficacy reported with these injectable agents. Although injectable therapies remain important for patients requiring intensive LDL‐C reduction, their use may be limited by cost, access, and the need for subcutaneous administration [45]. Therefore, oral combination therapies such as obicetrapib plus ezetimibe offer a convenient, non‐invasive alternative and may represent an effective treatment option for selected patients.

This study has several notable strengths, including a comprehensive search strategy, inclusion of multiple oral non‐statin therapies, and the evaluation of both efficacy and safety outcomes. The use of a frequentist network meta‐analysis enabled simultaneous comparison and ranking of multiple interventions. Methodological rigour was ensured through meta‐regression and application of the CINeMA framework to evaluate the certainty of evidence. Additionally, sensitivity analyses, including leave‐one‐out, confirmed the robustness and consistency of the findings.

Despite these strengths, several limitations should be acknowledged. High statistical heterogeneity was observed across several efficacy outcomes (*I* ^2^ ≥ 90%). This heterogeneity likely reflects clinical diversity among the included trials, including differences in baseline LDL‐C levels, background statin therapy, follow‐up duration, and patient characteristics, as well as methodological variations such as sample size and treatment compliance. Although exploratory meta‐regression identified several study‐level factors associated with between‐study variability, these explained only part of the observed heterogeneity. Consequently, treatment rankings based on *P*‐scores should be interpreted with caution, particularly when the certainty of evidence is low or very low for the comparison. Although *P*‐scores provide a useful probabilistic ranking of relative treatment performance, they do not imply definitive superiority, especially when effect estimates are influenced by substantial heterogeneity, indirectness, and imprecision. In such settings, small differences in ranking should not be overinterpreted as clinically meaningful distinctions between therapies. Nevertheless, sensitivity analyses showed stable treatment rankings, and no significant global inconsistency was detected, supporting the overall robustness of the network estimates.

In addition, the analysis combined clinically heterogeneous populations, including statin‐intolerant patients, individuals receiving maximally tolerated statin therapy, and those not achieving LDL‐C targets despite statin treatment. These groups have very different baseline profiles. Statin‐intolerant patients typically start with higher baseline LDL‐C levels because they cannot take standard therapies and are more prone to side effects. In contrast, patients already on maximum statins have residual high cholesterol driven by biological resistance or severe baseline risk (like established cardiovascular disease). Because these underlying mechanisms and background therapies differ, individual treatment responses will vary. Since the included trials did not consistently report subgroup data so stratified network analyses were not feasible. Consequently, the pooled estimates should be interpreted as average treatment effects across diverse clinical populations rather than as effects specific to any single patient subgroup. Finally, the certainty of evidence was low for several outcomes because of imprecision and heterogeneity, which may reduce confidence in some estimates.

## 5 Implications for Practice and Future Research

This study has important clinical implications for dyslipidaemia management, particularly for patients who remain above LDL‐C targets despite maximally tolerated statins or who are statin intolerant. The marked lipid‐lowering effects of obicetrapib plus ezetimibe and bempedoic acid plus ezetimibe suggest that oral combination therapy may represent an effective escalation strategy before transitioning to injectable treatments. These findings also provide clinicians with comparative evidence to support individualized treatment decisions based on LDL‐C reduction needs, tolerability, patient preference, and treatment access, especially in settings where injectable therapies are unavailable or less acceptable. However, further long‐term and head‐to‐head trials are needed to determine whether these lipid improvements translate into cardiovascular benefit and to clarify the optimal sequencing of non‐statin therapies.

## 6 Conclusion

This network meta‐analysis showed that oral non‐statin combination therapies, particularly obicetrapib plus ezetimibe and bempedoic acid plus ezetimibe, achieve the greatest improvements in LDL‐C, non‐HDL‐C, and ApoB, making them promising options for patients who are statin‐intolerant or do not achieve recommended lipid targets However, these findings are limited to lipid outcomes and should not be interpreted as evidence of superior cardiovascular efficacy, particularly for obicetrapib‐based therapy, until cardiovascular outcome data become available. In addition, the certainty of evidence was low for several outcomes because of heterogeneity and imprecision, which may limit confidence in some estimates. Therefore, larger and longer‐term randomized controlled trials with cardiovascular outcome data are needed to confirm the durability of these lipid‐lowering effects and determine whether they translate into meaningful reductions in cardiovascular risk.

## Author Contributions

**Tamer Hodrob:** conceptualization, investigation, methodology, writing – original draft, writing – review and editing, project administration, formal analysis. **Reem J. Saad:** conceptualization, investigation, writing – original draft, methodology, project administration. **Aya Hamdy:** conceptualization, writing – original draft, methodology. **Mariam Saleh Alheneedy:** conceptualization, writing – original draft, methodology. **Ahmed Mohamed Shehata Abdelfattah Elsayed:** conceptualization, writing – original draft, methodology. **Elsaghir Ghazy:** conceptualization, writing – original draft, methodology. **Hazem Ayesh:** writing – review and editing, formal analysis, software, supervision.

## Funding

The authors have nothing to report.

## Conflicts of Interest

The authors declare no conflicts of interest.

## Supporting information


**Supporting Infomation S1:** Detailed search strategy.
**Supporting Infomation S2:** Meta‐analysis networks result for absolute changes in lipid parameters.
**Table S2.1:** Meta‐analysis networks result for absolute changes in lipid parameters.
**Figure S2.2:** Meta‐analysis networks result for absolute changes in lipid parameters.
**Supporting Infomation S3:** Network plots of treatment comparisons.
**Figure S3.1:** Network plot of treatment comparisons for percent LDL‐C.
**Figure S3.2:** Network plot of treatment comparisons for absolute LDL‐C.
**Figure S3.3:** Network plot of treatment comparisons for percent TC.
**Figure S3.4:** Network plot of treatment comparisons for absolute TC.
**Figure S3.5:** Network plot of treatment comparisons for percent HDL‐C.
**Figure S3.6:** Network plot of treatment comparisons for absolute HDL‐C.
**Figure S3.7:** Network plot of treatment comparisons for percent non‐HDL‐C.
**Figure S3.8:** Network plot of treatment comparisons for absolute non‐HDL‐C.
**Figure S3.9:** Network plot of treatment comparisons for percent TG.
**Figure S3.10:** Network plot of treatment comparisons for absolute TG.
**Figure S3.11:** Network plot of treatment comparisons for percent APO B.
**Figure S3.12:** Network plot of treatment comparisons for absolute APO B.
**Figure S3.13:** Network plot of treatment comparisons for percent APO A.
**Figure S3.14:** Network plot of treatment comparisons for serious adverse events.
**Figure S3.15:** Network plot of treatment comparisons treatment discontinuation.
**Figure S3.16:** Network plot of treatment comparisons for myalgia.
**Figure S3.17:** Network plot of treatment comparisons for headache.
**Figure S3.18:** Network plot of treatment comparisons for elevated liver enzymes.
**Supporting Infomation S4:** Baseline characteristics of the included studies.
**Table S4:** Study characteristics and outcomes in the included clinical trials.
**Supporting Infomation S5:** Baseline characteristics of participants.
**Table S5:** Baseline characteristics of participants.
**Supporting Infomation S6:** Risk of bias assessment of included trials.
**Table S6:** Risk of bias assessment.
**Supporting Infomation S7:** Publication bias (funnel plot).
**Figure S7.1:** Publication bias for percent LDL‐C.
**Figure S7.2:** Publication bias for absolute LDL‐C.
**Figure S7.3:** Publication bias for percent TC.
**Figure S7.4:** Publication bias for absolute TC.
**Figure S7.5:** Publication bias for percent HDL‐C.
**Figure S7.6:** Publication bias for absolute HDL‐C.
**Figure S7.7:** Publication bias for percent non‐HDL‐C.
**Figure S7.8:** Publication bias for absolute non‐HDL‐C.
**Figure S7.9:** Publication bias for percent TG.
**Figure S7.10:** Publication bias for absolute TG.
**Figure S7.11:** Publication bias for percent APO B.
**Figure S7.12:** Publication bias for absolute APO B.
**Figure S7.13:** Publication bias for percent APO A.
**Figure S7.14:** Publication bias for serious adverse events.
**Figure S7.15:** Publication bias for treatment discontinuation.
**Figure S7.16:** Publication bias for myalgia.
**Figure S7.17:** Publication bias for headache.
**Figure S7.18:** Publication bias for elevated liver enzymes.
**Supporting Infomation S8:** League table for each outcome.
**Table S8.1:** Percent LDL‐C league.
**Table S8.2:** Absolute LDL‐C league.
**Table S8.3:** Percent TC league.
**Table S8.4:** Absolute TC league.
**Table S8.5:** Percent HDL‐C league.
**Table S8.6:** Absolute HDL‐C league.
**Table S8.7:** Percent non‐HDL‐C league.
**Table S8.8:** Absolute non‐HDL‐C league.
**Table S8.9:** Percent TG league.
**Table S8.10:** Absolute TG league.
**Table S8.11:** Percent APO B league.
**Table S8.12:** Absolute APO B league.
**Table S8.13:** Percent APO A league.
**Table S8.14:** Serious adverse events league.
**Table S8.15:** Treatment discontinuation league.
**Table S8.16:** Myalgia league.
**Table S8.17:** Headache league.
**Table S8.18:** Elevated liver enzymes league.
**Supporting Infomation S9:** Treatment ranking for each outcome.
**Table S9.1:** Treatment ranking for percent LDL‐C.
**Table S9.2:** Treatment ranking for absolute LDL‐C.
**Table S9.3:** Treatment ranking for percent TC.
**Table S9.4:** Treatment ranking for absolute TC.
**Table S9.5:** Treatment ranking for percent HDL‐C.
**Table S9.6:** Treatment ranking for absolute HDL‐C.
**Table S9.7:** Treatment ranking for percent non‐HDL‐C.
**Table S9.8:** Treatment ranking for absolute non‐HDL‐C.
**Table S9.9:** Treatment ranking for percent TG.
**Table S9.10:** Treatment ranking for absolute TG.
**Table S9.11:** Treatment ranking for percent APO B.
**Table S9.12:** Treatment ranking for absolute APO B.
**Table S9.13:** Treatment ranking for percent APO A.
**Table S9.14:** Treatment ranking for serious adverse events.
**Table S9.15:** Treatment ranking for treatment discontinuation.
**Table S9.16:** Treatment ranking for myalgia.
**Table S9.17:** Treatment ranking for headache.
**Table S9.18:** Treatment ranking for elevated liver enzymes.
**Supporting Infomation S10:** Certainty of evidence for each outcome.
**Table S10.1:** Certainty of evidence for percent LDL‐C.
**Table S10.2:** Certainty of evidence for absolute LDL‐C.
**Table S10.3:** Certainty of evidence for percent TC.
**Table S10.4:** Certainty of evidence for absolute TC.
**Table S10.5:** Certainty of evidence for percent HDL‐C.
**Table S10.6:** Absolute HDL‐C.
**Table S10.7:** Certainty of evidence for percent non‐HDL‐C.
**Table S10.8:** Certainty of evidence for absolute non‐HDL‐C.
**Table S10.9:** Certainty of evidence for percent TG.
**Table S10.10:** Certainty of evidence for absolute TG.
**Table S10.11:** Certainty of evidence for percent APO B.
**Table S10.12:** Certainty of evidence for absolute APO B.
**Table S10.13:** Certainty of evidence for percent APO A.
**Table S10.14:** Certainty of evidence for serious adverse events.
**Table S10.15:** Certainty of evidence for treatment discontinuation.
**Table S10.16:** Certainty of evidence for myalgia.
**Table S10.17:** Certainty of evidence for headache.
**Table S10.18:** Certainty of evidence for elevated liver enzymes.
**Supporting Infomation S11:** Sensitivity analysis.
**Table S11.1:** Leave one out analysis for percent LDL‐C.
**Table S11.2:** Leave one out analysis for absolute LDL‐C.
**Table S11.3:** Leave one out analysis for percent TC.
**Table S11.4:** Leave one out analysis for absolute TC.
**Table S11.5:** Leave one out analysis for percent HDL‐C.
**Table S11.6:** Leave one out analysis for absolute HDL‐C.
**Table S11.7:** Leave one out analysis for percent non‐HDL‐C.
**Table S11.8:** Leave one out analysis for absolute non‐HDL‐C.
**Table S11.9:** Leave one out analysis for percent TG.
**Table S11.10:** Leave one out analysis for absolute TG.
**Table S11.11:** Leave one out analysis for percent APO B.
**Table S11.12:** Leave one out analysis for absolute APO B.
**Table S11.13:** Leave one out analysis for percent APO A.
**Table S11.14:** Leave one out analysis for serious adverse events.
**Table S11.15:** Leave one out analysis for treatment discontinuation.
**Table S11.16:** Leave one out analysis for myalgia.
**Table S11.17:** Leave one out analysis for headache.
**Table S11.18:** Leave one out analysis for elevated liver enzymes.
**Supporting Infomation S12:** PRISMA checklist.

## Data Availability

The data supporting the findings of this study are available from the corresponding author upon reasonable request.
