# Supplementary material for: Efficacy and Safety of Oral Non‐Statin Lipid‐Lowering Therapies in Dyslipidaemia: A Systematic Review and Network Meta‐Analysis
Source: Endocrinol Diabetes Metab. 2026 Sep 30;9(6):e70353. doi: 10.1002/edm2.70353 (PMC13627895; doi:10.1002/edm2.70353)
Supplement: Supplementary file 1 [file EDM2-9-e70353-s001.docx]

Table of Contents

*Supplement S1. Search strategy	6*

*Supplement S2. Meta-analysis networks result for absolute changes in lipid parameters.*

Table S2.1: Meta-analysis networks result for absolute changes in lipid parameters…….….….. 7

Figure S2.2: Meta-analysis networks result for absolute changes in lipid parameters…………....8

*Supplement S3. Network Plot of Treatment Comparisons	10*

Figure S3.1: Network Plot of Treatment Comparisons for Percent LDL-C	*10*

Figure S3.2. Network Plot of Treatment Comparisons for Absolute LDL-C	10

Figure S3.3. Network Plot of Treatment Comparisons for Percent TC ………………………………….11

Figure S3.4. Network Plot of Treatment Comparisons for Absolute TC………………………………..11

Figure S3.5. Network Plot of Treatment Comparisons for Percent HDL-C………………………….…12

Figure S3.6. Network Plot of Treatment Comparisons for Absolute HDL-C…………………………..12

Figure S3.7. Network Plot of Treatment Comparisons for Percent Non-HDL-C…………………….13

Figure S3.8. Network Plot of Treatment Comparisons for Absolute Non-HDL-C…………………..13

Figure S3.9. Network Plot of Treatment Comparisons for Percent TG………………………..………..14

Figure S3.10. Network Plot of Treatment Comparisons for Absolute TG……………………………….14

Figure S3.11. Network Plot of Treatment Comparisons for Percent APO B…………………….…….15

Figure S3.12. Network Plot of Treatment Comparisons for Absolute APO B…………………….…..15

Figure S3.13. Network Plot of Treatment Comparisons for Percent APO A…………….…….….…..16

Figure S3.14. Network Plot of Treatment Comparisons for serious adverse events	16

Figure S3.15. Network Plot of Treatment Comparisons treatment discontinuation	17

Figure S3.16. Network Plot of Treatment Comparisons for myalgia……………………………………..17

Figure S3.17. Network Plot of Treatment Comparisons for headache	18

Figure S3.18. Network Plot of Treatment Comparisons for Elevated liver enzymes	18

*Supplement S4. Baseline characteristics of the included studies	19*

Table S4: Study Characteristics and Outcomes in the included Clinical Trials	19

*Supplement S5. Baseline characteristics of participants	29*

Table S5: Baseline characteristics of participants	29

*Supplement S6. Risk of bias assessment of included trials	31*

Table S6: Risk of bias assessment	31

*Supplement S7. Publication bias (funnel plot)	35*

Figure S7.1: Percent LDL-C	*35*

Figure S7.2. Absolute LDL-C	35

Figure S7.3. Percent TC ………………………………………………………………………………………………………..36

Figure S7.4. Absolute TC…………………………………………………………………………………….…………………36

Figure S7.5. Percent HDL-C……………………………………………………………………………….………………….37

Figure S7.6. Absolute HDL-C……………………………………………………………………………….………………..37

Figure S7.7. Percent Non-HDL-C………………………………………………………………………….……………….38

Figure S7.8. Absolute Non-HDL-C…………………………………………………………………….…………………..38

Figure S7.9. Percent TG……………………………………………………………………………………….…….…………39

Figure S7.10. Absolute TG……………………………………………………………………………………..………….….39

Figure S7.11. Percent APO B…………………………………………………………………………………..…….………40

Figure S7.12. Absolute APO B……………………………………………………………………………………..…….….40

Figure S7.13. Percent APO A……………………………………………………………………………………….……..…41

Figure S7.14. Serious adverse events	41

Figure S7.15. Treatment discontinuation	42

Figure S7.16. Myalgia……………………………………………………………………………………………………………42

Figure S7.17. Headache	43

Figure S7.18. Elevated liver enzymes	43

*Supplement S8. League table for each outcome	44*

Table S8.1: Percent LDL-C	*44*

Table S8.2. Absolute LDL-C	45

Table S8.3. Percent TC …………………………………………………………………………………………………………46

Table S8.4. Absolute TC………………………………………………………………………………………………………..47

Table S8.5. Percent HDL-C……………………………………………………………………………………………………48

Table S8.6. Absolute HDL-C………………………………………………………………………………………………….49

Table S8.7. Percent Non-HDL-C……………………………………………………………………………………………50

Table S8.8. Absolute Non-HDL-C………………………………………………………………………………………….51

Table S8.9. Percent TG…………………………………………………………………………………………….………..…52

Table S8.10. Absolute TG………………………………………………………………………………………….………….53

Table S8.11. Percent APO B………………………………………………………………………………………….………54

Table S8.12. Absolute APO B………………………………………………………………………………………..……...55

Table S8.13. Percent APO A……………………………………………………………………………………………..…..56

Table S8.14. Serious adverse events	57

Table S8.15. Treatment discontinuation	58

Table S8.16. Myalgia………………………………………………………………………………………………………….…59

Table S8.17. Headache	60

Table S8.18. Elevated liver enzymes	61

*Supplement S9. Treatment ranking for each outcome	62*

Table S9.1: Percent LDL-C	*62*

Table S9.2. Absolute LDL-C	62

Table S9.3. Percent TC ………………………………………………………………………………………….……………..63

Table S9.4. Absolute TC…………………………………………………………………………………………………..……63

Table S9.5. Percent HDL-C………………………………………………………………………………………...………….64

Table S9.6. Absolute HDL-C…………………………………………………………………………………….…………….64

Table S9.7. Percent Non-HDL-C……………………………………………………………………………….……………65

Table S9.8. Absolute Non-HDL-C…………………………………………………………………………….…………….65

Table S9.9. Percent TG…………………………………………………………………………………………….……..……66

Table S9.10. Absolute TG……………………………………………………………………………………….…………….66

Table S9.11. Percent APO B………………………………………………………………………………….………………67

Table S9.12. Absolute APO B……………………………………………………………………………………….……….67

Table S9.13. Percent APO A………………………………………………………………………………………….………68

Table S9.14. Serious adverse events	68

Table S9.15. Treatment discontinuation	69

Table S9.16. Myalgia…………………………………………………………………………………………………….………69

Table S9.17. Headache	70

Table S9.18. Elevated liver enzymes	70

*Supplement S10. Certainty of evidence for each outcome	71*

Table S10.1: Percent LDL-C	*71*

Table S10.2. Absolute LDL-C	75

Table S10.3. Percent TC …………………………………………………………………………………….………………...80

Table S10.4. Absolute TC……………………………………………………………………………………….…………..…83

Table S10.5. Percent HDL-C………………………………………………………………………………………….……….86

Table S10.6. Absolute HDL-C………………………………………………………………………………………………..90

Table S10.7. Percent Non-HDL-C……………………………………………………………………………………….…93

Table S10.8. Absolute Non-HDL-C………………………………………………………………………………………..96

Table S10.9. Percent TG……………………………………………………..……………………………………………..…98

Table S10.10. Absolute TG………………………………………………………………………………..……………….102

Table S10.11. Percent APO B…………………………………………………………………………..…………………105

Table S10.12. Absolute APO B………………………………………………………………………….…………………109

Table S10.13. Percent APO A……………………………………………………………………………….……………..111

Table S10.14. Serious adverse events	114

Table S10.15. Treatment discontinuation	119

Table S10.16. Myalgia……………………………………………………………………………………………..….………125

Table S10.17. Headache	129

Table S10.18. Elevated liver enzymes	134

*Supplement S11. Sensitivity analysis	136*

Table S11.1: leave one out analysis for Percent LDL-C	*136*

Table S11.2. leave one out analysis for Absolute LDL-C	138

Table S11.3. leave one out analysis for Percent TC…………………………..………………………………..140

Table S11.4. leave one out analysis for Absolute TC……………………………………………..……………142

Table S11.5. leave one out analysis for Percent HDL-C……………………………………………………….143

Table S11.6. leave one out analysis for Absolute HDL-C……………………………….…………………….145

Table S11.7. leave one out analysis for Percent Non-HDL-C………………………………….……………147

Table S11.8. leave one out analysis for Absolute Non-HDL-C…………………………………………….149

Table S11.9. leave one out analysis for Percent TG……………………………………………………………150

Table S11.10. leave one out analysis for Absolute TG……………………………………………………….152

Table S11.11. leave one out analysis for Percent APO B……………………………………………………154

Table S11.12. leave one out analysis for Absolute APO B……………………………………………….….156

Table S11.13. leave one out analysis for Percent APO A……………………………………………….……157

Table S11.14. leave one out analysis for Serious adverse events	159

Table S11.15. leave one out analysis for Treatment discontinuation	161

Table S11.16. leave one out analysis for Myalgia……………………………………………….….……………163

Table S11.17. leave one out analysis for Headache	165

Table S11.18. leave one out analysis for Elevated liver enzymes	166

*Supplement S12. PRISMA check list	168*

*References	172*

**Supplement S1. Search strategy**

From inception to December 28, 2025:

****PubMed search Syntax: ****

( hypercholesterolemia OR hypercholesterolaemia OR dyslipidemia OR dyslipidaemia OR "mixed dyslipidemia" OR "mixed dyslipidaemia" OR "elevated LDL" OR "high LDL" ) AND ( ezetimibe OR bempedoic acid OR "bempedoic acid ezetimibe" OR Nexlizet OR Nustendi OR obicetrapib OR "TA-8995" OR colesevelam ) AND ( placebo OR "background therapy" OR "standard therapy" OR comparator ) AND (trial) AND ( LDL OR "LDL cholesterol" OR "LDL-C" OR "low-density lipoprotein" )

****** **Embase** **search Syntax: ****

( hypercholesterolemia OR hypercholesterolaemia OR dyslipidemia OR dyslipidaemia OR "mixed dyslipidemia" OR "mixed dyslipidaemia" OR "elevated LDL" OR "high LDL" ) AND ( ezetimibe OR bempedoic acid OR "bempedoic acid ezetimibe" OR Nexlizet OR Nustendi OR obicetrapib OR "TA-8995" OR colesevelam ) AND ( placebo OR "background therapy" OR "standard therapy" OR comparator ) AND (trial) AND ( LDL OR "LDL cholesterol" OR "LDL-C" OR "low-density lipoprotein" )

****** **Cochrane Library search Syntax: ****

( hypercholesterolemia OR hypercholesterolaemia OR dyslipidemia OR dyslipidaemia OR "mixed dyslipidemia" OR "mixed dyslipidaemia" OR "elevated LDL" OR "high LDL" ) AND ( ezetimibe OR bempedoic acid OR "bempedoic acid ezetimibe" OR Nexlizet OR Nustendi OR obicetrapib OR "TA-8995" OR colesevelam ) AND ( placebo OR "background therapy" OR "standard therapy" OR comparator ) AND (trial) AND ( LDL OR "LDL cholesterol" OR "LDL-C" OR "low-density lipoprotein" )

****** **Web of science search Syntax: ****

( hypercholesterolemia OR hypercholesterolaemia OR dyslipidemia OR dyslipidaemia OR "mixed dyslipidemia" OR "mixed dyslipidaemia" OR "elevated LDL" OR "high LDL" ) AND ( ezetimibe OR bempedoic acid OR "bempedoic acid ezetimibe" OR Nexlizet OR Nustendi OR obicetrapib OR "TA-8995" OR colesevelam ) AND ( placebo OR "background therapy" OR "standard therapy" OR comparator ) AND (trial) AND ( LDL OR "LDL cholesterol" OR "LDL-C" OR "low-density lipoprotein" )

| Outcome | Most Favorable Intervention | Treatment Effect | Least Favorable Intervention | Treatment Effect | Heterogenicity | Highest ranked treatment | Lowest ranked treatment |
| --- | --- | --- | --- | --- | --- | --- | --- |
| Absolute change in TC | Bempedoic acid 240 mg | MD: −44.70 mg/dl; (95% CI: −89.05 to −0.35) | Obicetrapib 10 mg | MD: 15.47; (95% CI: −16.71 to 47.65) | 89% | Bempedoic Acid 240 mg | Obicetrapib 10 mg |
| Absolute change in HDL-C | Obicetrapib 10 mg | MD: 85.07 mg/dL; (95% CI: 77.17 to 92.97) | Bempedoic acid 240 mg | MD: −1.00 mg/dL; (95% CI: −10.23 to 8.23) | 16.9% | Bempedoic Acid 240 mg | Obicetrapib 10 mg |
| Absolute change in non-HDL-C | Bempedoic acid 240 mg | MD: −43.90 mg/dL; (95% CI: −69.93 to −17.87) | Colesevelam 3.75 g | MD: −13.58 mg/dL; (95% CI: −34.18 to 7.01) | 45.7% | Bempedoic Acid 240 mg | Placebo |
| Absolute change in TG | Bempedoic Acid 180 mg + Ezetimibe 10 mg | MD: −10.72 mg/dL; (95% CI: −14.89 to −6.56) | Bempedoic acid 240 mg | MD: 16.40 mg/dL; (95% CI: −43.87 to 76.67) | 0.0% | Bempedoic acid 180 mg + Ezetimibe 10 mg | Colesevelam 4.5 g |
| Absolute change in APO B | Obicetrapib 10 mg | MD: −40.00 mg/dL; (95% CI: −50.07 to −29.93) | Ezetimibe 10 mg | MD: −11.38 mg/dL; (95% CI: −15.19 to −7.57) | 61.4% | Obicetrapib 10 mg | Placebo |

**Table S2.1:** Meta-analysis networks result for absolute changes in lipid parameters

**D**

**C**

**B**

**A**

**E**

**Figure S2.2:** Meta-analysis networks result for **(A)** Absolute change in TC**, (B)** Absolute change in HDL-C, **(C)** Absolute change in Non-HDL-C, **(D)** Absolute change in TG, **(E)** Absolute change in APO B: Effect sizes are presented as mean difference (MD) and 95% confidence intervals (CI).

**Supplement S3: Network plots of treatment comparisons**

Figure S3.1: Network Plot of Treatment Comparisons for percent LDL-C

FigureS3.2: Network Plot of Treatment Comparisons for absolute LDL-C

FigureS3.3: Network Plot of Treatment Comparisons for percent TC

FigureS3.4: Network Plot of Treatment Comparisons for absolute TC

FigureS3.5: Network Plot of Treatment Comparisons for percent HDL-C

FigureS3.6: Network Plot of Treatment Comparisons for absolute HDL-C

FigureS3.7: Network Plot of Treatment Comparisons for percent Non-HDL-C

FigureS3.8: Network Plot of Treatment Comparisons for absolute Non-HDL-C

FigureS3.9: Network Plot of Treatment Comparisons for percent TG

FigureS3.10: Network Plot of Treatment Comparisons for absolute TG

FigureS3.11: Network Plot of Treatment Comparisons for percent APO B

FigureS3.12: Network Plot of Treatment Comparisons for absolute APO B

FigureS3.13: Network Plot of Treatment Comparisons for percent APO A

Figure S3.14 Network Plot of Treatment Comparisons for serious adverse events

Figure S3.15 Network Plot of Treatment Comparisons treatment discontinuation

Figure S3.16 Network Plot of Treatment Comparisons for myalgia

Figure S3.17 Network Plot of Treatment Comparisons for headache

Figure S3.18 Network Plot of Treatment Comparisons for elevated liver enzymes

**Supplement S4**. Baseline characteristics of the included studies

Table S4: Study Characteristics and Outcomes in the included Clinical Trials

The table summarizes various clinical trials, detailing trial phase, registration, duration, treatment arms, and population characteristics.

| Study ID | Trial Phase | Year | NCT Code | Population Included | Population Excluded | Number of Participants | Intervention | Comparator | Duration (weeks) |
| --- | --- | --- | --- | --- | --- | --- | --- | --- | --- |
| Stein,2004 | Phase III | 2004 | NCT03867318 | Adults with primary hypercholesterolemia CHD, HeFH with an LDL-C level 130 mg/dL despite treatment with 10 mg/day of atorvastatin and diet | ALT or AST >2 times the ULN, significant renal or endocrine disease; pregnancy or lactation; advanced CHF, uncontrolled cardiac diseases within 3 months, or ongoing treatment with lipid-lowering agents other than 10 mg/day of atorvastatin | 621 | Ezetimibe 10 mg + Atorvastatin 40 mg | Atorvastatin 80 mg | 14 weeks |
| Marrazi,2024 | NR | 2024 | NR | Adults diagnosed with hypercholesterolemia treated with a combined lipid-lowering therapy including high-intensity statin plus ezetimibe for at least 3 months with persistent LDL-C levels above 70 mg/dL. | Renal failure, chronic liver diseases, cerebrovascular& cardiovascular diseases, cancers, triglycerides > 500 mg/Dl. | 120 | Bempedoic acid 180mg +statin + ezetimibe | Double-dose statin+ezetimibe | 12 weeks |
| goldberg,2019 | Phase III | 2019 | NCT02991118 | Adults with Fasting LDL-C ≥100 mg/dL, high cardiovascular risk, on maximally tolerated lipid-modifying therapy. | Total fasting triglyceride ≥500 mg/dL, renal failure, (BMI) ≥50kg/m2, significant cardiovascular disease. | 750 | Bempedoic acid 180 mg | Placebo | 52 weeks |
| Harada-Shiba,2024 | Phase II | 2024 | NCT05421078 | Adults with LDL-C > 70 mg/dL and TG < 400 mg/dL, Treated with a stable statin therapy | BMI > 35 kg/m2, Significant cardiovascular disease, HbA1c > 10%, Uncontrolled hypertension, Active muscle disease, GFR < 60 ml/min, Hepatic dysfunction, Anemia, Existing CETP deficiency, Homozygous Familial Hypercholesterolemia, cancer, Alcohol abuse, Treatment with PCSK9, Known CETP inhibitor allergy | 102 | Obicetrapib 10 mg | Placebo | 8 weeks |
| Ballantyne,2023 | Phase II | 2023 | NCT05266586 | Adults with LDL-C > 70 mg/dL and Triglycerides < 400 mg/dL,  Treated with a high-intensity statin therapy | BMI > 40 kg/m2, Significant cardiovascular disease, HbA1c >= 10%, Uncontrolled hypertension, Active muscle disease, GFR < 60 mL/min, Hepatic dysfunction, Anemia, malignancy, Alcohol abuse, Treatment with PCSK9, Known CETP inhibitor allergy | 119 | Obicetrapib 10 mg + Ezetimibe 10 mg  Obicetrapib 10 mg | Placebo | 12 weeks |
| Sarraju,2025 | Phase III | 2025 | NCT06005597 | Adults with ASCVD, on maximally tolerated lipid-modifying therapy, LDL-C ≥ 70 mg/dL, Triglycerides < 500 | class III or IV heart failure, MI, stroke, Uncontrolled HTN, homozygous FH, Liver disease, HbA1c ≥ 10.0%, TSH >1.5ULN, malignancy, CK>3 X ULN, Alcohol abuse, Treatment with gemfibrozil or ezetimibe. | 407 | Obicetrapib 10 mg + Ezetimibe 10 mg  Obicetrapib 10 mg  Ezetimibe 10 mg | Placebo | 12 weeks |
| Laufs,2019 | Phase III | 2019 | NCT02988115 | Adults with LDL-C ≥130 mg/dL for primary prevention or LDL-C ≥100 mg/dL for secondary prevention, and statin-intolerant. | Total fasting triglyceride ≥500 mg/dL, Renal dysfunction, BMI ≥50 kg/m2, cardiovascular disease or cardiovascular event in the past 3 months | 345 | Bempedoic Acid 180 mg | Placebo | 24 weeks |
| Ballantyne,2020 | Phase III | 2020 | NCT03337308 | Adults with LDL-C ≥ 130 mg/dL for primary prevention or LDL-C ≥ 100 mg/dL for secondary prevention, on maximally tolerated statin therapy for at least 4 weeks. | Triglyceride ≥ 400 mg/dL, Renal failure, cardiovascular disease or cardiovascular event within the past 3 months | 382 | Bempedoic Acid 180 mg + Ezetimibe 10 mg  Ezetimibe 10 mg  Bempedoic Acid 180 mg | Placebo | 12 weeks |
| Ballantyne,2016 | Phase II | 2016 | NCT02072161 | Adults with LDL-C ≥130 mg/dL and ≤220 mg/dL, TG level ≤400 mg/dL, on statin therapy for at least 3 months. | Cardiovascular, liver, GI, hematological or renal diseases, muscle-related symptoms due to ongoing statin therapy, uncontrolled DM, Use of metformin or thiazolidinediones within 4 weeks of screening, Uncontrolled hypothyroidism, HIV, malignancy, drug or alcohol abuse within 2 years. | 134 | Bempedoic Acid 180 mg | Placebo | 12 weeks |
| Ray,2019 | Phase III | 2019 | NCT02666664 | Adults with ASCVD or HeFH on stable statin therapy whose LDL-C remained ≥70 mg/dl | Participants were excluded if they were receiving gemfibrozil or simvastatin at doses >40 mg/day. PCSK9 inhibitors were not allowed prior to enrollment but could be initiated after week 24 if LDL-C exceeded 170 mg/dL and increased by ≥25% from baseline. | 2229 | Bempedoic Acid 180 mg | Placebo | 52 weeks |
| Hovingh,2015 | Phase II | 2015 | NCT01970215 | Adults with LDL-C levels >2.5 mmol/L, not on lipid-altering therapy at screening or on lipid-altering treatment regimens at screening | BMI >32 kg/m2, clinical manifestation of atherosclerotic vascular disease, Uncontrolled DM Uncontrolled hypertension, hyperaldosteronism, Active muscle disease. | 364 | Obicetrapib 10 mg  Obicetrapib 10 mg + Atorvastatin 20 mg  Obicetrapib 10 mg + Rosuvastatin 10 mg  Atorvastatin 20 mg | Placebo | 12 weeks |
| Teramoto,2012 | Phase IV | 2012 | NCT00871351 | Adults with LDL-C levels that had not reached the lipid management target values during treatment with stain | triglyceride> 400 mg/dL, CPK >2 X ULN, HbA1c >=8％, severe hepatic disorder, homozygous FH. hypersensitivity to ezetimibe, atorvastatin, or rosuvastatin. pregnant or lactating, cyclosporine treatment, hypothyroidism, renal failure, pancreatitis, or cancer. | 125 | Ezetimibe 10 mg + Atorvastatin 10 mg | Atorvastatin 20 mg  Rosuvastatin 2.5 mg | 20 weeks |
| Yang,2017 | NR | 2017 | NR | Adults with coronary artery disease, LDL-C level >100 mg/dL, individuals with 10-year risk of 10% to 20%, and LDL-C >130 mg/dL. | history of acute coronary syndrome, cerebrovascular disease, or symptomatic peripheral artery disease, uncontrolled hypertension, DM or arrhythmia, thyroid dysfunction, coagulopathy, renal failure, cancer, history of myopathy, Pregnancy, or breast-feeding. | 245 | Ezetimibe 10 mg + Rosuvastatin 20 mg | Rosuvastatin 20 mg | 12 weeks |
| Nicholls,2017 | Phase III | 2017 | NCT02227784 | Adults with LDL-C levels that had not reached the lipid management target values during treatment with stain | Have a hemoglobin A1c (HbA1c) >9.5%, NYHA class III or IV CHF  History of either a transient ischemic stroke or ischemic stroke <30 days  History of ACS <30 days. | 366 | Ezetimibe 10 mg + Atorvastatin 40 mg | Atorvastatin 40 mg  Atorvastatin 80 mg | 12 weeks |
| Sasaki,2012 | Phase III | 2012 | NR | Adults with LDL-C ≥120 mg/dL after treatment with pravastatin for 4 weeks or longer. | Using drugs for hyperlipidemia other pravastatin, insulin, thiazolidinediones, hypersensitivity to pravastatin, liver or kidney diseases, ACS, Stroke, Pregnancy, Familial hypercholesterolemia or secondary hyperlipidemia | 198 | Ezetimibe 10 mg + Pravastatin 10-40 mg | Double-dose pravastatin | 12 weeks |
| insull,2001 | Phase III | 2001 | NR | Adults with LDL cholesterol ≥130 mg/dL and ≤220 mg/dL and serum triglycerides ≤300 mg/dL) | any clinically important unstable medical condition. | 494 | Colesevelam 4.5 g | Placebo | 24 weeks |
| Sakamoto,2017 | Phase III | 2017 | NR | Japanese Adults with LDL-C levels that had not reached the lipid management target values during treatment with stain | Pregnant or lactating, Hypersensitive, Liver dysfunction, HbA1C≥9.4%, renal failure, Triglyceride ≥ 400 mg/dL, Secondary dyslipidemia, drug-induced dyslipidemia, Homozygotic FH | 109 | Ezetimibe 10 mg + Statin | Double-dose statin | 52 weeks |
| RUGGENENTI,2010 | Phase II | 2010 | NCT00157482 | Adults with type 2 diabetes, total cholesterol concentrations 135 mg/dl despite lipid-lowering therapy | Cardiovascular, liver or muscle diseases, primary hyperlipidemia, pregnancy or lactating, treated with steroids, immunosuppressive agents, fibrates, niacin, or cholestyramine. | 108 | Ezetimibe 10 mg | Placebo | 8 weeks |
| Lee,2021 | Phase III | 2021 | NCT04652349 | Adults with fasting serum levels of LDL-C ≤250 mg/dL and triglyceride levels < 500 mg/dL | ACS, advanced HF, stroke, type uncontrolled DM, hypertension, active liver disease, thyroid disease, or cancers, impaired drug absorption or history of alcohol abuse, history of muscle diseases. pregnancy or breastfeeding. | 279 | Ezetimibe 10 mg + Rosuvastatin 2.5 mg  Rosuvastatin 5 mg  Ezetimibe 10 mg | Rosuvastatin 2.5 mg | 8 weeks |
| Moreira,2014 | Phase 4 | 2014 | NR | Adults with LDL-C levels that had not reached the lipid management target values during treatment with stain | Patients with liver, renal or GI disease, malignancies, uncontrolled metabolic disorder. | 112 | Ezetimibe 10 mg + Simvastatin 40 mg | Rosuvastatin 40 mg | 12 weeks |
| Ballantyne,2018 | Phase III | 2018 | NCT03001076 | Adults with a history of statin intolerance, and required additional lowering of LDL-C | History of CVD, uncontrolled hypertension, revascularization procedure, Class IV heart failure, cerebrovascular event, symptomatic PAD | 269 | Bempedoic Acid 180 mg | Placebo | 12 weeks |
| Yamashita,2025 | Phase III | 2025 | NR | Adults with LDL-C levels that had not reached the lipid management target values during treatment with stain | homozygous FH, cardiovascular disease, uncontrolled DM or HTN, hematologic disorders, hypothyroidism or liver disease. History of chronic musculoskeletal symptoms, renal dysfunction or prior GI surgery. | 96 | Bempedoic Acid 180 mg | Placebo | 12 weeks |
| Yokote,2017 | Phase II | 2017 | NCT02055976 | Adults with LDL-C levels that had not reached the lipid management target values during treatment with stain | Pregnant or lactating, chronic medical or psychiatric condition or laboratory abnormality, History of cardiovascular or cerebrovascular events, CHF, poorly controlled DM or HTN, hypothyroidism, alcoholism or drug addiction, or cancers. | 121 | Bococizumab 50,100,150mg  Ezetimibe 10 mg | Placebo | 16 weeks |
| Gagne´,2002 | Phase III | 2002 | NR | Adults with primary hypercholesterolemia, currently taking a stable daily dose of a statin for 6 weeks and on a cholesterol-lowering diet | Heart failure, cardiovascular disease, or severe peripheral artery disease within the past 3 months, uncontrolled DM, Uncontrolled endocrine or metabolic disease, renal failure; active or chronic hepatic or hepatobiliary disease. | 769 | Ezetimibe 10 mg + Statin | Statin + Placebo | 8 weeks |
| ballantyne,2004_a | Phase III | 2004 | NR | Adults with a LDL cholesterol level at or above drug treatment threshold | Patients who didn’t met the criteria | 788 | Ezetimibe 10 mg + Simvastatin 10,20,40,80 mg | Atorvastatin 10,20,40,80 mg | 28 weeks |
| GOLDBERG,2004 | Phase III | 2004 | NR | Adults with LDL-C levels that had not reached the lipid management target values during treatment with stain liver transaminase <2X ULN, CK <1.5 times ULN | HX of CHF, cardiovascular diseases, uncontrolled DM or HTN, renal failure, coagulation abnormalities, taking non statin lipid-lowering drugs, immunosuppressant drugs, corticosteroids. | 886 | Ezetimibe 10 mg + Simvastatin 80 mg  Ezetimibe 10 mg  Simvastatin 80 mg | Placebo | 12 weeks |
| Farnier,2005 | Phase III | 2005 | NR | Adults with mixed hyperlipidemia and no coronary heart disease or CHD-equivalent disease (except for type 2 diabetes), or 10-year  CHD risk =20% | homozygous FH, type I or V hyperlipidemia, CHF, cardiovascular disease uncontrolled DM or HTN pancreatitis, gall bladder, renal or active liver disease, pregnancy or lactation. | 652 | Fenofibrate 160 mg  + Ezetimibe 10 mg  Fenofibrate 160 mg  Ezetimibe 10 mg | Placebo | 12 weeks |
| Qian,2022_a+b | Phase III | 2022 | NCT03768427 | Chinese adults with LDL-C levels that had not reached the lipid management target values during treatment with stain | Patients with uncontrolled hypertriglyceridemia requiring drug intervention or with a triglyceride level of ≥500 mg/dL, liver disease, Class III-IVHF, endocrine or metabolic disease. | 454 | Ezetimibe 10 mg + Atorvastatin 10,20 mg | Atorvastatin 20,40 mg | 12 weeks |
| Stein,2008 | phase 4 | 2008 | NCT00125125 | Adults with dyslipidemia who had previously documented MRSEs that had led to cessation of statin treatment. | Homozygous FH, muscle diseases, MRSEs with fluvastatin/AST> 2x ULN, renal failure, cardiovascular diseases or stroke within 6 months. | 199 | Ezetimibe 10 mg + Fluvastatin 80 mg  Fluvastatin 80 mg | Ezetimibe 10 mg | 12 weeks |
| Torimoto,2013 | NR | 2013 | NR | Adults with LDL-C levels that had not reached the lipid management target values during treatment with stain | Patients with a history of FH, stroke, ischemic heart disease within the past six months, liver or kidney failure, patients on insulin, pregnant or breast feeding | 75 | Ezetimibe 10 mg + Rosuvastatin 2.5 mg | Rosuvastatin 5 mg | 12 weeks |
| Ballantyne,2014 | Phase III | 2014 | NCT00525824 | Adults with fasting LDL-C 130 to <220 mg/dl and fasting  triglycerides < 400 mg/dl | History of statin-induced myopathy, cardiovascular diseases, stroke; had severe CHF, malignancy, homozygous FH, active liver disease, uncontrolled hypothyroidism, pregnant or breastfeeding women. | 833 | Ezetimibe 10 mg + Rosuvastatin 20 mg  Ezetimibe 10 mg + Simvastatin 80 mg | Ezetimibe 10 mg + Rosuvastatin 10 mg  Ezetimibe 10 mg + Simvastatin 40 mg | 12 weeks |
| Yu,2012 | Phase 4 | 2012 | NCT00652327 | Triglycerides ≤400 mg/dL, liver transaminases  ≤2 X ULN, and  CK level ≤2 X ULN. | Pregnant or lactating women. History of mental illness, drug or alcohol abuse,  active liver or renal disease, cardiovascular diseases, hypertension, diabetes, endocrine or metabolic disorders. | 83 | Ezetimibe 10 mg + Ongoing statin | Double-dose statin | 8 weeks |
| Stein,2007 | NR | 2007 | NR | Adults with LDL-C levels that had not reached the lipid management target values during treatment with stain | Not meeting the criteria | 109 | Ezetimibe 10 mg + Rosuvastatin 40 mg | Rosuvastatin 40 mg | 12 weeks |
| Bajaj,2020 | phase 4 | 2020 | NCT02682680 | Adults with T2DM whose LDL-C levels remained above recommended lipid targets despite treatment with statins. | triglyceride level ≥5.0 mmol/L, CK or ALT ≥ 3x ULN, pregnant or lactating, renal failure a history of severe gastroparesis or significant bowel resection. | 200 | Colesevelam 3.75 g | Ezetimibe 10 mg | 24 weeks |
| Luo,2016 | NR | 2016 | NR | Adults with LDL-C levels that had not reached the lipid management target values during treatment with lipid-lowering therapy | Patients with blood diseases, liver or renal dysfunction, severe infectious  diseases, or heart failure. | 148 | Ezetimibe 10 mg + Atorvastatin 20 mg | Atorvastatin 20 mg | 48 weeks |
| ballantyne,2004_b | NR | 2004 | NR | patients who completed the initial trial and chose to join the long-term study. | completers of the initial trial who did not enroll in the extension, plus any patients who dropped out of the initial 12-week study. | 246 | Ezetimibe 10 mg + Atorvastatin 10 mg | Atorvastatin 10 mg | 48 weeks |
| Ward,2022 | NR | 2022 | NR | Adults with documented statin intolerance and not  achieving ideal lipid goals | History of cardiovascular diseases, elevated creatine kinase, pregnant or lactating women history of necrotizing auto-immune myositis or autoantibodies against HMG-CoA reductase. | 50 | Nutraceutical+ Ezetimibe 10 mg combination  Nutraceutical  Ezetimibe 10 mg | Placebo | 8 weeks |
| Nicholls,2025 | Phase III | 2025 | NCT05142722 | Patients with an LDL cholesterol level > 100 mg/dl and HDL cholesterol level > 130 mg/dl, as well as those with an LDL-C 55 - 100 mg/dl or a non-HDL cholesterol level of 85 - 130 mg/dl and at least one additional cardiovascular risk facto | History of cardiovascular event within the previous 3 months class III or IV heart failure, uncontrolled HTN or DM. Homozygous FH, active liver disease or History of cancer. | 2530 | Obicetrapib 10 mg | Placebo | 48 weeks |
| Efrati,2007 | NR | 2007 | NR | Adults with LDL-C levels that had not reached the lipid management target values during treatment with lipid-lowering therapy | Cardiovascular diseases or cerebrovascular event, uncontrolled HTN or DM, heart failure, chronic or acute inflammatory conditions, serum triglycerides >250 mg/dL, abnormal liver function tests, CK levels exceeding the upper normal value | 40 | Ezetimibe 10 mg + Simvastatin 40 mg  Simvastatin 80mg  Ezetimibe 10mg | Simvastatin 40mg | 12 weeks |
| Moon,2023 | Phase 4 | 2023 | NCT03403556 | Adults aged 40 to 75 years old, with T2DM, estimated  10-year ASCVD risk ≥7.5%, BMI< 35 kg/m2 and HbA1c levels <10% | Type 1 diabetes mellitus or renal failure. | 106 | Ezetimibe 10 mg + Rosuvastatin 10 mg | Rosuvastatin 20 mg | 24 weeks |
| Strony,2008 | NR | 2008 | NR | Adults with primary hypercholesterolemia who completed a previous 12-week trial of ezetimibe plus statin therapy but did not achieve target LDL-C levels. | Those who had adverse events, poor compliance, interfering conditions, or used other lipid-lowering/interaction medications. | 436 | Ezetimibe 10 mg+ Pravastatin 10-40 mg | Ezetimibe 10 mg + Simvastatin 10-80 mg | 48 weeks |
| Thompson,2016 | phase IIb | 2016 | NCT01941836 | Adults with documented statin intolerance and tolerance and not  achieving ideal lipid goals | Cardiovascular disease  uncontrolled DM, hypothyroidism, non–statin-related musculoskeletal complaints, liver or renal dysfunction, unexplained CK elevation, or using  anticoagulants, systemic corticosteroids, cyclosporine, metformin, or thiazolidinediones within 3 months | 349 | Bempedoic Acid 180 mg  Bempedoic Acid 120 mg + Ezetimibe 10 mg  Bempedoic Acid 180 mg + Ezetimibe 10 mg  Bempedoic Acid 120 mg | Ezetimibe 10 mg | 12 weeks |
| Thompson,2015 | phase II | 2015 | NR | Adults with hypercholesterolemia and a history of intolerance to statin. | Type I DM, Cardiovascular diseases within 12 months, uncontrolled HTN, hypothyroidism, liver or renal dysfunction, or unexplained CK elevations >3 x ULN | 56 | Bempedoic Acid 60-240 mg | Placebo | 8 weeks |

Abbreviations used include: DM: Diabetes Mellites, HeFH: Heterozygous Familial Hypercholesterolemia, CHF: Congestive Heart Failure, BMI: Body Mass Index, ASCVD: Atherosclerotic Cardiovascular Disease, ACS: Acute Coronary Syndrome, HTN: Hypertension

Supplement S5. Baseline characteristics of participants

Table S5: Baseline characteristics of participants

| Study Id | **Age**  **(mean, SD)** | **Male%** | **White%** | **BMI**  **(mean, SD)** | **LDL-C**  **(mean, SD)** | **HDL-C**  **(mean, SD)** | **Non-HDL-C**  **(mean, SD)** | **TC**  **(mean, SD)** | **TG**  **(mean, SD)** |
| --- | --- | --- | --- | --- | --- | --- | --- | --- | --- |
| Stein,2004^1^ | 51.49±10.95 | 53.14% | 91.46% | NR | 186.76±46.64 | 49.95±12.32 | 213.22±48.41 | 263.12±47.55 | 114.05±86.32 |
| Marrazi,2024^2^ | 61.8±9.45 | 55% | NR | 27.85±6.78 | 88.7±8.41 | 37.05±4.39 | 110.9±27.34 | 147.4±46.42 | 108.8±32.92 |
| goldberg,2019^3^ | 64.3±8.77 | 61.40% | 94.30% | 30.20±5.14 | 120.39±37.9 | 51.3±12.96 | 151.69±43.26 | 202.99±43.84 | 144.61±63.99 |
| Harada-Shiba,2024^4^ | 62.9±9.59 | 75% | NR | 25.80±3.74 | 109.5±34.54 | 54.05±14.29 | 134.5±33.64 | NR | 126±48.64 |
| Ballantyne,2023^5^ | 64.09±8.25 | 63.40% | 91% | 30.93±5.17 | 101.11±30.86 | 53.22±17.05 | 125.87±32.52 | NR | 149.23±57.86 |
| Sarraju,2025^6^ | 67.83±8.41 | 61.76% | 82.26% | NR | 98.62±32.91 | 48.33±15.55 | 125.68±42.48 | NR | 125.79±66.41 |
| Laufs,2019^7^ | 65.17±9.53 | 67.59% | 51.21% | 30.26±5.61 | 157.57±39.86 | 51.62±14.47 | 192.6±44.64 | 244.22±46.34 | 165.21±77.82 |
| Ballantyne,2020^8^ | 65.05±9.11 | 47.70% | 81.60% | 30.25±4.98 | 146.92±39.97 | 50.65±14.18 | 177.85±43.91 | 228.32±47.04 | 150.52±69.79 |
| Ballantyne,2016^9^ | 56.5±9.96 | 41.11% | 82.22% | 30.5±5.99 | 136.5±25.64 | 54.5±14.44 | NR | 220.5±27.81 | 137.17±60.04 |
| Ray,2019^10^ | 66.13±8.95 | 73% | 73% | NR | 103.17±29.40 | 48.9±11.77 | 130.4±33.77 | 179.33±35.26 | 129.67±54.96 |
| Hovingh,2015^11^ | 65±5.9 | 86.25% | 97.5 | 25.5±2.28 | 116.01±21.27 | 38.67±11.60 | NR | 193.35±21.27 | 88.57±57.57 |
| Teramoto,2012^12^ | 61.02±11.66 | 49.46% | NR | 25.44±3.84 | 214.2±42.12 | 52.25±11.53 | 249.23±44.23 | 301.49±45.85 | 156.79±68.05 |
| Yang.2017^13^ | 63.74±9.19 | 67.90% | NR | 25.29±3.09 | 148.96±33.88 | 46.88±10.9 | NR | 221.41±38.33 | 149.07±71.57 |
| Nicholls,2017^14^ | 63.4 ± 9.2 | 66.10% | NR | 31.7 ± 7.0 | 83±32.8 | 46±25.35 | 119±44.73 | NR | 130±131.2 |
| Sasaki,2012^15^ | 67.64±9.72 | 30.80% | NR | 24.15±3.54 | 137.77±22.76 | 57±12.98 | 166.58±27.43 | 223.58±28.57 | 128.78±66.68 |
| insull,2001^16^ | 56±12 | 50% | 90% | 29±6 | 155±17.94 | 49.98±12.83 | NR | 240.97±22.95 | 167.60±71.03 |
| Sakamoto,2017^17^ | 62.16±10.27 | 57.80% | NR | NR | 132.96±21.05 | 55.67±12.62 | 159.89±26.45 | 215.43±28.66 | 154.51±91.61 |
| RUGGENENTI,2010^18^ | 65.4 | 59% | NR | 28.9±4.13 | 95±29.67 | 49±11.5 | NR | 158±33.22 | 114.5±81.46 |
| Lee,2021^19^ | 63.45±10.03 | 54.80% | NR | 25.45±7.03 | 138.64±27.94 | 49.24±12.73 | 156.41±29.43 | 205.66±31.93 | 153.06±77.01 |
| Moreira,2014^20^ | NR | NR | NR | NR | 160.51±41.02 | 53.51±14.91 | NR | 247.07±44.88 | 163.21±76.11 |
| Ballantyne,2018^21^ | 63.77±10.95 | 38.60% | 89.20% | 29.83±5.10 | 127.58±29.86 | 56.23±18.06 | 158.87±34.85 | 215.06±36.05 | 151.14±68.50 |
| Yamashita,2025^22^ | 64.2±12.93 | 46.90% | NR | 24.26±3.19 | 136.08±23.39 | 57.36±14.43 | 161.48±24.27 | 218.83±25.95 | 119.29±59.36 |
| Yokote,2017^23^ | 58.2±10.86 | 52% | NR | 25.19±3 | 135.65±24.46 | 57.83±12.51 | 164.8±25.66 | 222.62±28.46 | 143.55±57.82 |
| Gagne´,2002^24^ | 57.37±10.29 | 57.60% | 90.10% | 38.11±10.56 | 138.51±41.15 | 49.51±11.77 | 169±42.15 | 218.51±43.11 | 136.51±77.39 |
| ballantyne,2004_a^25^ | 60.03±10.51 | 52% | 90.30% | NR | 179.93±42.86 | 46.77±11.44 | 218.63±44.61 | 265.43±46.49 | 174±98.92 |
| GOLDBERG,2004^26^ | NR | 48.50% | 81.30% | NR | 175±26.01 | 50.01±12.55 | 210±30.49 | 259.51±29.98 | 168.01±90.99 |
| Farnier,2005^27^ | 53.75±9.62 | 62.50% | NR | 29.4±4.6 | 159.53±27.07 | 42.54±7.71 | 216.55±29.95 | 259.09±30.88 | 270.05±100.32 |
| Qian,2022_a^28^ | 62.4±7.95 | 54.20% | NR | 25.25±3.35 | 96.15±25.2 | 47.1±11.98 | 124.81±26.9 | 171.91±28.1 | 143.12±59.85 |
| Qian,2022_b^28^ | 60.01±9.82 | 71.10% | NR | 25.95±3.5 | 90.17±23.49 | 44.5±10.03 | 118.22±25.75 | 162.72±27.1 | 140.55±63.19 |
| Stein,2008^29^ | 60.99± 9.87 | 47% | NR | 28.3±4.49 | 175.18±44.12 | 53.01±14.7 | NR | 265.74±48.92 | 186.01±94.18 |
| Torimoto,2013^30^ | 64.72±12.37 | 53% | NR | 25.77±4.09 | 111.48±24.01 | 56.52±13.75 | NR | NR | 147±74.43 |
| Ballantyne,2014^31^ | 61.9±9.55 | 55.15% | NR | 31±6.54 | 163.97±24.40 | 48.15±12.71 | 201.55±27.31 | 249.7±27.84 | 187.34±80.66 |
| Yu,2012^32^ | 57.98±11.17 | 55.55% | NR | NR | 137.21±34.57 | 50.61±10.82 | NR | 210.2±38.85 | 150.12±64.51 |
| Stein,2007^33^ | 53±14 | 66% | 94% | NR | 291±59 | 45±11 | 331±61 | NR | 194±108 |
| Bajaj,2020^34^ | 59.44±10.24 | 48.50% | NR | 29.8±6.67 | 97.24±22.77 | 45.6±11.15 | 125.61±27.10 | NR | 138.68±63.25 |
| Luo,2016^35^ | 61.16±10.65 | 56.70% | NR | 24.96±5.05 | 137.08±16.28 | 45.63±16.61 | NR | 229.31±56.30 | 223.13±48.58 |
| ballantyne,2004_b^36^ | 57.76±10.69 | 41.05% | NR | NR | 182.45±23.20 | 53.43±14.89 | NR | 267.53±27.06 | 164.3±67.7 |
| Ward,2022^37^ | 61.8±8.80 | 53.84% | NR | 27.7±3.71 | 188.51±39.22 | 53.56±13.13 | 222.74±46.62 | 275.91±43.42 | 174.87±101.32 |
| Nicholls,2025^38^ | 65.37±9.80 | 66.28% | 74.62% | 29.5±5.50 | 98.2±37.36 | 49.57±14.83 | 125±43.80 | 174.57±44.80 | 128.78±59.90 |
| Efrati,2007^39^ | 57.5±12.19 | 80% | NR | 27±3.59 | 130±33.82 | 54.85±17.34 | NR | NR | 126.9±63.70 |
| Moon,2023^40^ | 61.51±6.77 | 65.65% | NR | 25.74±3.21 | 135.81±30.74 | 48.33±10.92 | 155.6±33.42 | NR | 195.98±124.15 |
| Strony,2008^41^ | 56.6±11.78 | 42.64% | 87% | 29.13±5.50 | 178.35±20.89 | 51.1±11.67 | NR | 264.45±24.92 | 175.35±60.74 |
| Thompson,2016^42^ | 59.5±9.50 | 48.50% | NR | 30.5±5.01 | 165.5±24.45 | 50.51±12.57 | NR | 250.51±32.52 | 205.25±264.48 |
| Thompson,2015^43^ | 62.64±6.40 | 50% | 96.42% | 29.66±4.35 | 179.05±35.65 | 53.38±15.67 | NR | 267.41±44.38 | 188.46±146.03 |

**Table S5:** The table summarizes the baseline characteristics of patients included in the study. Characteristics include the age, male participants, Race (White), body mass index (BMI), LDL-C, HDL-C, non-HDL, TC, and TG. Mean values with SD are provided for continuous variables, while percentages are provided for categorical variables. Abbreviations**:** BMI: Body Mass Index, LDL-C: Low-density lipoprotein cholesterol, HDL-C: High-density lipoprotein cholesterol, Non-HDL-C: Non-High-density lipoprotein cholesterol, TC: Total cholesterol, TG Triglycerides, NR: Not Reported

| **Study ID**  **Supplement S6.** Risk of bias assessment of included trials | **Randomization process** | **Deviations from Intended Interventions** | **Missing outcome data** | **Selection of the reported result** | **Measurement of the Outcome** | **Overall Judgment** |
| --- | --- | --- | --- | --- | --- | --- |
| Stein,2004 | Low risk | Low risk | Low risk | Low risk | Low risk | Low risk |
| Marrazi,2024 | Low risk | Low risk | Low risk | Low risk | Low risk | Low risk |
| goldberg,2019 | Low risk | Low risk | Low risk | Low risk | Low risk | Low risk |
| Harada-Shiba,2024 | Low risk | Low risk | Low risk | Low risk | Low risk | Low risk |
| Ballantyne,2023 | Low risk | Low risk | Low risk | Low risk | Low risk | Low risk |
| Sarraju,2025 | Low risk | Low risk | Low risk | Low risk | Low risk | Low risk |
| Laufs,2019 | Low risk | Some concerns | Some concerns | Low risk | Low risk | Some concerns |
| Ballantyne,2020 | Low risk | Low risk | Low risk | Low risk | Low risk | Some concerns |
| Ballantyne,2016 | Low risk | Low risk | Low risk | Low risk | Low risk | Low risk |
| Ray,2019 | Low risk | Low risk | Low risk | Low risk | Low risk | Low risk |
| Hovingh,2015 | Low risk | Low risk | Low risk | Low risk | Low risk | Low risk |
| Teramoto,2012 | Low risk | Low risk | Low risk | Low risk | Low risk | Low risk |
| Yang,2017 | Low risk | Low risk | Low risk | Low risk | Low risk | Low risk |
| Nicholls,2017 | Low risk | Low risk | Low risk | Low risk | Low risk | Low risk |
| Yamashita,2025 | Low Risk | Low Risk | Low Risk | Low Risk | Low risk | Low risk |
| ballantyne,2004_a | Low Risk | Low Risk | Low Risk | Low Risk | Low risk | Low risk |
| Ballantyne,2018 | Low Risk | Low Risk | Low Risk | Low Risk | Low risk | Low risk |
| Farnier,2005 | Low Risk | Low Risk | Low Risk | Low Risk | Low risk | Low risk |
| Gagne´,2002 | Low Risk | Low Risk | Low Risk | Low Risk | Low risk | Low risk |
| GOLDBERG,2004 | Low Risk | Low Risk | Low Risk | Low Risk | Low risk | Low risk |
| insull,2001 | High Risk | Low Risk | Low Risk | Low Risk | Low risk | High Risk |
| Lee,2021 | Low Risk | Low Risk | Low Risk | Low Risk | Low risk | Low risk |
| Moreira,2014 | High Risk | Low Risk | Low Risk | Low Risk | Low risk | High Risk |
| Qian,2022_a+b | Low Risk | Low Risk | Low Risk | Low Risk | Low risk | Low risk |
| RUGGENENTI,2010 | Low Risk | Low Risk | Low Risk | Low Risk | Low risk | Low risk |
| Sakamoto,2017 | Low Risk | Low Risk | Low Risk | Low Risk | Low risk | Low risk |
| Sasaki,2012 | High Risk | Low Risk | Low Risk | Low Risk | Low risk | High Risk |
| Yokote,2017 | Low Risk | Low Risk | Low Risk | Low Risk | Low risk | Low risk |
| Stein,2008 | Low risk | Low risk | Low risk | Low risk | Low risk | Low risk |
| Torimoto,2013 | Low risk | Low Risk | Low risk | Low risk | Low risk | Low risk |
| Bajaj,2020 | Low risk | Low risk | Low risk | Low risk | Low risk | Low risk |
| ballantyne,2004_b | Low risk | Low risk | Low risk | Low risk | Low risk | Low risk |
| Ballantyne,2014 | Low risk | Low risk | Low risk | Low risk | Low risk | Low risk |
| Efrati,2007 | High risk | Low risk | Low risk | Low risk | Low risk | High risk |
| Luo,2016 | Low risk | Low risk | Low risk | Low risk | Low risk | Low risk |
| Moon,2023 | Low risk | Low risk | Low risk | Low risk | Low risk | Low risk |
| Nicholls,2025 | Low risk | Low risk | Low risk | Low risk | Low risk | Low risk |
| Stein,2007 | High risk | Low risk | Low risk | Low risk | Low risk | High risk |
| Strony,2008 | High risk | Low risk | Low risk | Low risk | Low risk | High risk |
| Thompson,2015 | Low risk | Low risk | Low risk | Low risk | Low risk | Low risk |
| Thompson,2016 | Low risk | Low risk | Low risk | Low risk | Low risk | Low risk |
| Ward,2022 | Low risk | Low risk | Low risk | Low risk | Low risk | Low risk |
| Yu,2012 | Low risk | Low risk | Some concerns | Low risk | Low risk | Some concerns |

**Supplement S7. Publication bias (funnel plot)**

Figure S7.1: Percent LDL-C

Figure S7.2: Absolute LDL-C

Figure S7.3: Percent TC

Figure S7.4: Absolute TC

Figure S7.5: Percent HDL-C

Figure S7.6: Absolute HDL-C

Figure S7.7: Percent Non-HDL-C

Figure S7.8: Absolute Non-HDL-C

Figure S7.9: Percent TG

Figure S7.10: Absolute TG

Figure S7.11: Percent APO B

Figure S7.12: Absolute APO B

Figure S7.13: Percent APO A

Figure S7.14: Serious adverse events

Figure S7.15: Treatment discontinuation

Figure S7.16: Myalgia

Figure S7.17: Headache

Figure S7.18: Elevated liver enzymes

**Supplement S8: League table for each outcome**

|  | Bempedoic Acid 180 mg | Bempedoic Acid 180 mg + Ezetimibe 10 mg | Bempedoic Acid 240 mg | Colesevelam 4.5 g | Ezetimibe 10 mg | Obicetrapib 10 mg | Obicetrapib 10 mg + Ezetimibe 10 mg | Placebo |
| --- | --- | --- | --- | --- | --- | --- | --- | --- |
| Bempedoic Acid 180 mg | Bempedoic Acid 180 mg | 18.22 [ 8.15; 28.29] | . | . | -9.00 [-22.14; 4.14] | . | . | -21.04 [-26.66; -15.42] |
| Bempedoic Acid 180 mg + Ezetimibe 10 mg | 16.01 [ 8.42; 23.61] | Bempedoic Acid 180 mg + Ezetimibe 10 mg | . | . | -19.92 [-29.90; -9.93] | . | . | . |
| Bempedoic Acid 240 mg | 7.81 [ -7.29; 22.91] | -8.20 [-24.45; 8.04] | Bempedoic Acid 240 mg | . | . | . | . | -28.70 [-42.98; -14.42] |
| Colesevelam 4.5 g | -2.89 [-16.82; 11.04] | -18.90 [-34.06; -3.74] | -10.70 [-30.03; 8.63] | Colesevelam 4.5 g | . | . | . | -18.00 [-31.03; -4.97] |
| Ezetimibe 10 mg | -6.08 [-11.48; -0.67] | -22.09 [-29.67; -14.51] | -13.89 [-28.52; 0.74] | -3.19 [-16.60; 10.22] | Ezetimibe 10 mg | . | 27.90 [ 12.06; 43.74] | -14.46 [-17.83; -11.10] |
| Obicetrapib 10 mg | 13.36 [ 5.41; 21.31] | -2.65 [-12.57; 7.26] | 5.55 [-10.05; 21.15] | 16.25 [ 1.78; 30.71] | 19.44 [ 12.55; 26.33] | Obicetrapib 10 mg | 18.32 [ 6.79; 29.84] | -35.38 [-42.00; -28.75] |
| Obicetrapib 10 mg + Ezetimibe 10 mg | 28.26 [ 16.97; 39.56] | 12.25 [ -0.41; 24.91] | 20.45 [ 2.87; 38.04] | 31.15 [ 14.57; 47.74] | 34.34 [ 23.99; 44.69] | 14.90 [ 5.28; 24.52] | Obicetrapib 10 mg + Ezetimibe 10 mg | . |
| Placebo | -20.89 [-25.81; -15.97] | -36.90 [-44.65; -29.15] | -28.70 [-42.98; -14.42] | -18.00 [-31.03; -4.97] | -14.81 [-17.99; -11.63] | -34.25 [-40.53; -27.97] | -49.15 [-59.42; -38.89] | Placebo |

Table S8.1: League table for percent LDL-C

Table S8.2: League table for absolute LDL-C

|  | Bempedoic acid 180 mg | Bempedoic Acid 180 mg | Bempedoic Acid 180 mg+Ezetimibe 10 mg | Bempedoic Acid 240 mg | Colesevelam 3.75 g | Colesevelam 4.5 g | Ezetimibe 10 mg | Obicetrapib 10 mg | Placebo |
| --- | --- | --- | --- | --- | --- | --- | --- | --- | --- |
| Bempedoic acid 180 mg | Bempedoic acid 180 mg | . | . | . | . | . | . | . | -14.30 [-42.09; 13.49] |
| Bempedoic Acid 180 mg | 13.53 [-18.39; 45.45] | Bempedoic Acid 180 mg | . | . | . | . | . | . | -27.83 [-43.53; -12.13] |
| Bempedoic Acid 180 mg+Ezetimibe 10 mg | 31.52 [ -7.82; 70.86] | 17.99 [-13.98; 49.96] | Bempedoic Acid 180 mg+Ezetimibe 10 mg | . | . | . | -27.20 [-53.78; -0.62] | . | . |
| Bempedoic Acid 240 mg | 34.10 [ -8.32; 76.52] | 20.57 [-15.12; 56.26] | 2.58 [-39.87; 45.04] | Bempedoic Acid 240 mg | . | . | . | . | -48.40 [-80.45; -16.35] |
| Colesevelam 3.75 g | -5.74 [-45.76; 34.27] | -19.27 [-52.07; 13.52] | -37.26 [-75.55; 1.03] | -39.84 [-82.92; 3.24] | Colesevelam 3.75 g | . | 10.06 [-17.51; 37.63] | . | . |
| Colesevelam 4.5 g | 13.70 [-25.08; 52.48] | 0.17 [-31.10; 31.44] | -17.82 [-56.64; 21.00] | -20.40 [-62.33; 21.53] | 19.44 [-20.06; 58.94] | Colesevelam 4.5 g | . | . | -28.00 [-55.04; -0.96] |
| Ezetimibe 10 mg | 4.32 [-24.69; 33.33] | -9.21 [-26.98; 8.55] | -27.20 [-53.78; -0.62] | -29.78 [-62.89; 3.33] | 10.06 [-17.51; 37.63] | -9.38 [-37.67; 18.91] | Ezetimibe 10 mg | . | -18.62 [-26.93; -10.31] |
| Obicetrapib 10 mg | 28.53 [ -5.35; 62.40] | 15.00 [ -9.94; 39.93] | -2.99 [-36.91; 30.93] | -5.57 [-43.02; 31.87] | 34.27 [ -0.43; 68.97] | 14.83 [-18.43; 48.09] | 24.21 [ 3.13; 45.28] | Obicetrapib 10 mg | -42.83 [-62.19; -23.46] |
| Placebo | -14.30 [-42.09; 13.49] | -27.83 [-43.53; -12.13] | -45.82 [-73.67; -17.97] | -48.40 [-80.45; -16.35] | -8.56 [-37.35; 20.23] | -28.00 [-55.04; -0.96] | -18.62 [-26.93; -10.31] | -42.83 [-62.19; -23.46] | Placebo |

|  | Bempedoic Acid 180 mg | Bempedoic Acid 180 mg + Ezetimibe 10 mg | Bempedoic Acid 240 mg | Colesevelam 4.5 g | Ezetimibe 10 mg | Obicetrapib 10 mg | Placebo |
| --- | --- | --- | --- | --- | --- | --- | --- |
| Bempedoic Acid 180 mg | Bempedoic Acid 180 mg | 13.93 [ 7.53; 20.34] | . | . | -6.40 [-14.53; 1.73] | . | -12.96 [-16.28; -9.64] |
| Bempedoic Acid 180 mg + Ezetimibe 10 mg | 12.91 [ 8.07; 17.74] | Bempedoic Acid 180 mg + Ezetimibe 10 mg | . | . | -15.35 [-21.71; -8.99] | . | . |
| Bempedoic Acid 240 mg | 5.33 [ -4.76; 15.41] | -7.58 [-18.42; 3.26] | Bempedoic Acid 240 mg | . | . | . | -18.50 [-28.14; -8.86] |
| Colesevelam 4.5 g | -2.17 [-10.93; 6.59] | -15.08 [-24.70; -5.46] | -7.50 [-20.18; 5.18] | Colesevelam 4.5 g | . | . | -11.00 [-19.24; -2.76] |
| Ezetimibe 10 mg | -3.46 [ -6.89; -0.02] | -16.36 [-21.19; -11.54] | -8.79 [-18.72; 1.15] | -1.29 [ -9.87; 7.29] | Ezetimibe 10 mg | . | -9.84 [-12.38; -7.30] |
| Obicetrapib 10 mg | -26.90 [-33.72; -20.08] | -39.81 [-47.71; -31.91] | -32.23 [-43.66; -20.80] | -24.73 [-35.01; -14.45] | -23.45 [-30.04; -16.86] | Obicetrapib 10 mg | 13.73 [ 7.59; 19.88] |
| Placebo | -13.17 [-16.13; -10.21] | -26.08 [-31.04; -21.12] | -18.50 [-28.14; -8.86] | -11.00 [-19.24; -2.76] | -9.71 [-12.10; -7.33] | 13.73 [ 7.59; 19.88] | Placebo |

Table S8.3: League table for percent TC

|  | Bempedoic Acid 180 mg | Bempedoic acid 180 mg + Ezetimibe 10 mg | Bempedoic Acid 240 mg | Colesevelam 4.5 g | Ezetimibe 10 mg | Obicetrapib 10 mg | Placebo |
| --- | --- | --- | --- | --- | --- | --- | --- |
| Bempedoic Acid 180 mg | Bempedoic Acid 180 mg | . | . | . | . | . | -21.10 [-51.51; 9.31] |
| Bempedoic acid 180 mg + Ezetimibe 10 mg | 12.06 [ -32.54; 56.67] | Bempedoic acid 180 mg + Ezetimibe 10 mg | . | . | -15.40 [-45.74; 14.94] | . | . |
| Bempedoic Acid 240 mg | 23.60 [ -30.17; 77.37] | 11.54 [ -43.53; 66.60] | Bempedoic Acid 240 mg | . | . | . | -44.70 [-89.05; -0.35] |
| Colesevelam 4.5 g | 4.90 [ -38.43; 48.23] | -7.16 [ -52.09; 37.76] | -18.70 [ -72.74; 35.34] | Colesevelam 4.5 g | . | . | -26.00 [-56.87; 4.87] |
| Ezetimibe 10 mg | -3.34 [ -36.04; 29.37] | -15.40 [ -45.74; 14.94] | -26.94 [ -72.89; 19.02] | -8.24 [ -41.37; 24.90] | Ezetimibe 10 mg | . | -17.76 [-29.81; -5.72] |
| Obicetrapib 10 mg | -36.57 [ -80.84; 7.70] | -48.63 [ -94.47; -2.80] | -60.17 [-114.97; -5.37] | -41.47 [ -86.06; 3.12] | -33.23 [ -67.59; 1.12] | Obicetrapib 10 mg | 15.47 [-16.71; 47.65] |
| Placebo | -21.10 [ -51.51; 9.31] | -33.16 [ -65.80; -0.53] | -44.70 [ -89.05; -0.35] | -26.00 [ -56.87; 4.87] | -17.76 [ -29.81; -5.72] | 15.47 [ -16.71; 47.65] | Placebo |

Table S8.4: League table for absolute TC

|  | Bempedoic Acid 180 mg | Bempedoic Acid 180 mg + Ezetimibe 10 mg | Bempedoic Acid 240 mg | Colesevelam 4.5 g | Ezetimibe 10 mg | Obicetrapib 10 mg | Obicetrapib 10 mg + Ezetimibe 10 mg | Placebo |
| --- | --- | --- | --- | --- | --- | --- | --- | --- |
| Bempedoic Acid 180 mg | Bempedoic Acid 180 mg | -1.10 [ -12.15; 9.95] | . | . | -9.80 [ -19.54; -0.06] | . | . | -4.16 [ -9.26; 0.94] |
| Bempedoic Acid 180 mg + Ezetimibe 10 mg | 0.04 [ -8.12; 8.21] | Bempedoic Acid 180 mg + Ezetimibe 10 mg | . | . | -8.70 [ -19.75; 2.35] | . | . | . |
| Bempedoic Acid 240 mg | 0.77 [ -12.45; 13.98] | 0.73 [ -14.24; 15.69] | Bempedoic Acid 240 mg | . | . | . | . | -5.80 [ -18.26; 6.66] |
| Colesevelam 4.5 g | -9.03 [ -19.78; 1.72] | -9.07 [ -21.91; 3.76] | -9.80 [ -25.66; 6.06] | Colesevelam 4.5 g | . | . | . | 4.00 [ -5.81; 13.81] |
| Ezetimibe 10 mg | -7.52 [ -12.26; -2.77] | -7.56 [ -15.72; 0.61] | -8.28 [ -21.02; 4.46] | 1.52 [ -8.64; 11.68] | Ezetimibe 10 mg | . | . | 2.23 [ -0.52; 4.98] |
| Obicetrapib 10 mg | -152.86 [-161.08; -144.64] | -152.90 [-163.71; -142.10] | -153.63 [-167.89; -139.37] | -143.83 [-155.84; -131.81] | -145.35 [-152.77; -137.92] | Obicetrapib 10 mg | 7.00 [ -16.19; 30.19] | 147.83 [ 140.89; 154.77] |
| Obicetrapib 10 mg + Ezetimibe 10 mg | -145.86 [-170.47; -121.25] | -145.90 [-171.49; -120.32] | -146.63 [-173.86; -119.40] | -136.83 [-162.95; -110.71] | -138.35 [-162.70; -113.99] | 7.00 [ -16.19; 30.19] | Obicetrapib 10 mg + Ezetimibe 10 mg | . |
| Placebo | -5.03 [ -9.43; -0.63] | -5.07 [ -13.35; 3.21] | -5.80 [ -18.26; 6.66] | 4.00 [ -5.81; 13.81] | 2.48 [ -0.16; 5.13] | 147.83 [ 140.89; 154.77] | 140.83 [ 116.62; 165.04] | Placebo |

Table S8.5: League table for percent HDL-C

Table S8.6: League table for absolute HDL-C

|  | Bempedoic acid 180 mg | Bempedoic Acid 240 mg | Colesevelam 3.75 g | Colesevelam 4.5 g | Ezetimibe 10 mg | Obicetrapib 10 mg | Placebo |
| --- | --- | --- | --- | --- | --- | --- | --- |
| Bempedoic acid 180 mg | Bempedoic acid 180 mg | . | . | . | . | . | 0.40 [ -1.77; 2.57] |
| Bempedoic Acid 240 mg | 1.40 [ -8.08; 10.88] | Bempedoic Acid 240 mg | . | . | . | . | -1.00 [-10.23; 8.23] |
| Colesevelam 3.75 g | -1.15 [ -4.56; 2.26] | -2.55 [-12.15; 7.05] | Colesevelam 3.75 g | . | . | . | 1.55 [ -1.08; 4.18] |
| Colesevelam 4.5 g | -0.93 [ -4.17; 2.31] | -2.33 [-11.87; 7.21] | 0.22 [ -3.35; 3.79] | Colesevelam 4.5 g | . | . | 1.33 [ -1.08; 3.74] |
| Ezetimibe 10 mg | -0.70 [ -3.37; 1.96] | -2.10 [-11.47; 7.26] | 0.45 [ -2.61; 3.50] | 0.23 [ -2.64; 3.09] | Ezetimibe 10 mg | . | 1.10 [ -0.45; 2.66] |
| Obicetrapib 10 mg | -84.67 [-92.87; -76.47] | -86.07 [-98.22; -73.92] | -83.52 [-91.85; -75.19] | -83.74 [-92.00; -75.48] | -83.97 [-92.02; -75.91] | Obicetrapib 10 mg | 85.07 [ 77.17; 92.97] |
| Placebo | 0.40 [ -1.77; 2.57] | -1.00 [-10.23; 8.23] | 1.55 [ -1.08; 4.18] | 1.33 [ -1.08; 3.74] | 1.10 [ -0.45; 2.66] | 85.07 [ 77.17; 92.97] | Placebo |

Table S8.7: League table for percent Non-HDL-C

|  | Bempedoic Acid 180 mg | Bempedoic Acid 180 mg + Ezetimibe 10 mg | Bempedoic Acid 240 mg | Ezetimibe 10 mg | Obicetrapib 10 mg | Obicetrapib 10 mg + Ezetimibe 10 mg | Placebo |
| --- | --- | --- | --- | --- | --- | --- | --- |
| Bempedoic Acid 180 mg | Bempedoic Acid 180 mg | 17.44 [ 9.18; 25.70] | . | -6.60 [-17.31; 4.11] | . | . | -15.95 [-20.32; -11.58] |
| Bempedoic Acid 180 mg + Ezetimibe 10 mg | 16.78 [ 10.49; 23.07] | Bempedoic Acid 180 mg + Ezetimibe 10 mg | . | -18.02 [-26.27; -9.77] | . | . | . |
| Bempedoic Acid 240 mg | 4.45 [ -8.36; 17.26] | -12.33 [-26.16; 1.50] | Bempedoic Acid 240 mg | . | . | . | -21.00 [-33.20; -8.80] |
| Ezetimibe 10 mg | -1.89 [ -6.58; 2.80] | -18.67 [-24.96; -12.39] | -6.34 [-19.05; 6.36] | Ezetimibe 10 mg | . | 25.40 [ 12.27; 38.53] | -14.87 [-18.82; -10.92] |
| Obicetrapib 10 mg | 10.65 [ 3.59; 17.71] | -6.13 [-14.84; 2.57] | 6.20 [ -7.37; 19.77] | 12.54 [ 5.86; 19.21] | Obicetrapib 10 mg | 17.56 [ 8.00; 27.13] | -27.92 [-34.28; -21.56] |
| Obicetrapib 10 mg + Ezetimibe 10 mg | 26.58 [ 17.10; 36.06] | 9.80 [ -0.83; 20.42] | 22.13 [ 7.10; 37.16] | 28.47 [ 19.60; 37.35] | 15.93 [ 7.86; 24.00] | Obicetrapib 10 mg + Ezetimibe 10 mg | . |
| Placebo | -16.55 [-20.46; -12.64] | -33.33 [-39.85; -26.81] | -21.00 [-33.20; -8.80] | -14.66 [-18.20; -11.11] | -27.20 [-33.14; -21.25] | -43.13 [-51.91; -34.35] | Placebo |

|  | Bempedoic Acid 180 mg | Bempedoic acid 180 mg + Ezetimibe 10 mg | Bempedoic Acid 240 mg | Colesevelam 3.75 g | Ezetimibe 10 mg | Placebo |
| --- | --- | --- | --- | --- | --- | --- |
| Bempedoic Acid 180 mg | Bempedoic Acid 180 mg | . | . | . | . | -18.40 [-33.82; -2.98] |
| Bempedoic acid 180 mg + Ezetimibe 10 mg | -1.60 [-23.35; 20.15] | Bempedoic acid 180 mg + Ezetimibe 10 mg | . | . | . | -16.80 [-32.14; -1.46] |
| Bempedoic Acid 240 mg | 25.50 [ -4.76; 55.76] | 27.10 [ -3.11; 57.31] | Bempedoic Acid 240 mg | . | . | -43.90 [-69.93; -17.87] |
| Colesevelam 3.75 g | -4.82 [-30.54; 20.91] | -3.22 [-28.89; 22.46] | -30.32 [-63.51; 2.88] | Colesevelam 3.75 g | 11.60 [ -6.12; 29.32] | . |
| Ezetimibe 10 mg | 6.78 [-11.87; 25.43] | 8.38 [-10.20; 26.97] | -18.72 [-46.78; 9.35] | 11.60 [ -6.12; 29.32] | Ezetimibe 10 mg | -25.18 [-35.68; -14.69] |
| Placebo | -18.40 [-33.82; -2.98] | -16.80 [-32.14; -1.46] | -43.90 [-69.93; -17.87] | -13.58 [-34.18; 7.01] | -25.18 [-35.68; -14.69] | Placebo |

Table S8.8: League table for absolute Non-HDL-C

Table S8.9: League table for percent TG

|  | Bempedoic Acid 180 mg | Bempedoic Acid 180 mg + Ezetimibe 10 mg | Bempedoic Acid 240 mg | Colesevelam 4.5 g | Ezetimibe 10 mg | Obicetrapib 10 mg | Obicetrapib 10 mg + Ezetimibe 10 mg | Placebo |
| --- | --- | --- | --- | --- | --- | --- | --- | --- |
| Bempedoic Acid 180 mg | Bempedoic Acid 180 mg | 9.50 [-24.49; 43.49] | . | . | 4.30 [-21.78; 30.38] | . | . | 0.26 [-11.60; 12.12] |
| Bempedoic Acid 180 mg + Ezetimibe 10 mg | 10.68 [-13.67; 35.02] | Bempedoic Acid 180 mg + Ezetimibe 10 mg | . | . | -5.20 [-38.02; 27.62] | . | . | . |
| Bempedoic Acid 240 mg | -18.95 [-50.39; 12.48] | -29.63 [-68.00; 8.74] | Bempedoic Acid 240 mg | . | . | . | . | 18.60 [-10.99; 48.19] |
| Colesevelam 4.5 g | -4.35 [-28.62; 19.91] | -15.03 [-47.78; 17.72] | 14.60 [-22.17; 51.37] | Colesevelam 4.5 g | . | . | . | 4.00 [-17.83; 25.83] |
| Ezetimibe 10 mg | 6.57 [ -4.91; 18.06] | -4.10 [-28.36; 20.15] | 25.53 [ -4.64; 55.69] | 10.93 [-11.67; 33.52] | Ezetimibe 10 mg | . | . | -7.08 [-13.09; -1.08] |
| Obicetrapib 10 mg | -0.07 [-16.47; 16.33] | -10.75 [-38.19; 16.70] | 18.88 [-13.25; 51.02] | 4.28 [-20.88; 29.45] | -6.64 [-20.46; 7.17] | Obicetrapib 10 mg | 8.49 [-19.60; 36.58] | -0.28 [-12.80; 12.24] |
| Obicetrapib 10 mg + Ezetimibe 10 mg | 8.42 [-24.11; 40.94] | -2.26 [-41.53; 37.01] | 27.37 [-15.31; 70.05] | 12.77 [-24.94; 50.48] | 1.85 [-29.46; 33.15] | 8.49 [-19.60; 36.58] | Obicetrapib 10 mg + Ezetimibe 10 mg | . |
| Placebo | -0.35 [-10.95; 10.24] | -11.03 [-35.45; 13.39] | 18.60 [-10.99; 48.19] | 4.00 [-17.83; 25.83] | -6.93 [-12.78; -1.08] | -0.28 [-12.80; 12.24] | -8.77 [-39.52; 21.98] | Placebo |

|  | Bempedoic acid 180 mg + Ezetimibe 10 mg | Bempedoic Acid 240 mg | Colesevelam 3.75 g | Colesevelam 4.5 g | Ezetimibe 10 mg | Obicetrapib 10 mg | Placebo |
| --- | --- | --- | --- | --- | --- | --- | --- |
| Bempedoic acid 180 mg + Ezetimibe 10 mg | Bempedoic acid 180 mg + Ezetimibe 10 mg | . | . | . | -2.30 [ -3.50; -1.10] | . | . |
| Bempedoic Acid 240 mg | -27.12 [-87.54; 33.29] | Bempedoic Acid 240 mg | . | . | . | . | 16.40 [-43.87; 76.67] |
| Colesevelam 3.75 g | -14.69 [-35.54; 6.16] | 12.43 [-51.45; 76.32] | Colesevelam 3.75 g | . | 12.39 [ -8.42; 33.20] | . | . |
| Colesevelam 4.5 g | -17.72 [-30.22; -5.23] | 9.40 [-52.01; 70.81] | -3.03 [-27.28; 21.21] | Colesevelam 4.5 g | . | . | 7.00 [ -4.78; 18.78] |
| Ezetimibe 10 mg | -2.30 [ -3.50; -1.10] | 24.82 [-35.58; 85.23] | 12.39 [ -8.42; 33.20] | 15.42 [ 2.99; 27.86] | Ezetimibe 10 mg | . | -8.42 [-12.42; -4.43] |
| Obicetrapib 10 mg | -1.92 [-31.68; 27.83] | 25.20 [-41.88; 92.28] | 12.77 [-23.53; 49.06] | 15.80 [-15.93; 47.53] | 0.38 [-29.35; 30.10] | Obicetrapib 10 mg | -8.80 [-38.26; 20.66] |
| Placebo | -10.72 [-14.89; -6.56] | 16.40 [-43.87; 76.67] | 3.97 [-17.23; 25.16] | 7.00 [ -4.78; 18.78] | -8.42 [-12.42; -4.43] | -8.80 [-38.26; 20.66] | Placebo |

Table S8.10: League table for absolute TG

|  | Bempedoic Acid 180 mg | Bempedoic Acid 180 mg + Ezetimibe 10 mg | Bempedoic Acid 240 mg | Colesevelam 4.5 g | Ezetimibe 10 mg | Obicetrapib 10 mg | Obicetrapib 10 mg + Ezetimibe 10 mg | Placebo |
| --- | --- | --- | --- | --- | --- | --- | --- | --- |
| Bempedoic Acid 180 mg | Bempedoic Acid 180 mg | 13.37 [ 4.75; 21.98] | . | . | -6.10 [-17.24; 5.04] | . | . | -14.64 [-19.07; -10.20] |
| Bempedoic Acid 180 mg + Ezetimibe 10 mg | 12.60 [ 6.07; 19.13] | Bempedoic Acid 180 mg + Ezetimibe 10 mg | . | . | -14.76 [-23.32; -6.20] | . | . | . |
| Bempedoic Acid 240 mg | 0.36 [-14.28; 15.00] | -12.24 [-27.85; 3.37] | Bempedoic Acid 240 mg | . | . | . | . | -15.30 [-29.39; -1.21] |
| Colesevelam 4.5 g | -2.94 [-14.78; 8.91] | -15.54 [-28.57; -2.51] | -3.30 [-21.27; 14.67] | Colesevelam 4.5 g | . | . | . | -12.00 [-23.15; -0.85] |
| Ezetimibe 10 mg | -2.91 [ -7.68; 1.86] | -15.51 [-22.03; -9.00] | -3.27 [-17.80; 11.25] | 0.03 [-11.68; 11.73] | Ezetimibe 10 mg | . | 14.20 [ 1.59; 26.81] | -11.80 [-15.74; -7.87] |
| Obicetrapib 10 mg | 7.25 [ 0.79; 13.71] | -5.35 [-13.72; 3.02] | 6.89 [ -8.11; 21.88] | 10.19 [ -2.09; 22.47] | 10.16 [ 4.14; 16.18] | Obicetrapib 10 mg | 11.29 [ 2.12; 20.45] | -23.07 [-28.50; -17.64] |
| Obicetrapib 10 mg + Ezetimibe 10 mg | 16.03 [ 6.98; 25.08] | 3.43 [ -6.93; 13.79] | 15.67 [ -0.66; 32.00] | 18.97 [ 5.09; 32.85] | 18.94 [ 10.55; 27.34] | 8.78 [ 1.08; 16.48] | Obicetrapib 10 mg + Ezetimibe 10 mg | . |
| Placebo | -14.94 [-18.92; -10.95] | -27.54 [-34.28; -20.80] | -15.30 [-29.39; -1.21] | -12.00 [-23.15; -0.85] | -12.03 [-15.57; -8.48] | -22.19 [-27.33; -17.05] | -30.97 [-39.24; -22.70] | Placebo |

Table S8.11: League table for percent APO B

|  | Bempedoic Acid 180 mg | Bempedoic Acid 240 mg | Colesevelam 4.5 g | Ezetimibe 10 mg | Obicetrapib 10 mg | Placebo |
| --- | --- | --- | --- | --- | --- | --- |
| Bempedoic Acid 180 mg | Bempedoic Acid 180 mg | . | . | . | . | -11.40 [-19.20; -3.60] |
| Bempedoic Acid 240 mg | 6.40 [-11.48; 24.28] | Bempedoic Acid 240 mg | . | . | . | -17.80 [-33.89; -1.71] |
| Colesevelam 4.5 g | 7.60 [ -4.60; 19.80] | 1.20 [-17.42; 19.82] | Colesevelam 4.5 g | . | . | -19.00 [-28.38; -9.62] |
| Ezetimibe 10 mg | -0.02 [ -8.70; 8.66] | -6.42 [-22.95; 10.11] | -7.62 [-17.74; 2.50] | Ezetimibe 10 mg | . | -11.38 [-15.19; -7.57] |
| Obicetrapib 10 mg | 28.60 [ 15.87; 41.33] | 22.20 [ 3.22; 41.18] | 21.00 [ 7.24; 34.76] | 28.62 [ 17.86; 39.38] | Obicetrapib 10 mg | -40.00 [-50.07; -29.93] |
| Placebo | -11.40 [-19.20; -3.60] | -17.80 [-33.89; -1.71] | -19.00 [-28.38; -9.62] | -11.38 [-15.19; -7.57] | -40.00 [-50.07; -29.93] | Placebo |

Table S8.12: League table for absolute APO B

|  | Bempedoic Acid 180 mg | Bempedoic Acid 180 mg + Ezetimibe 10 mg | Bempedoic Acid 240 mg | Colesevelam 4.5 g | Ezetimibe 10 mg | Obicetrapib 10 mg | Placebo |
| --- | --- | --- | --- | --- | --- | --- | --- |
| Bempedoic Acid 180 mg | Bempedoic Acid 180 mg | 4.20 [ -6.25; 14.65] | . | . | -1.90 [-11.75; 7.95] | . | 3.60 [ -7.54; 14.74] |
| Bempedoic Acid 180 mg + Ezetimibe 10 mg | 4.99 [ -3.16; 13.15] | Bempedoic Acid 180 mg + Ezetimibe 10 mg | . | . | -6.10 [-16.77; 4.57] | . | . |
| Bempedoic Acid 240 mg | 4.92 [ -8.90; 18.75] | -0.07 [-14.81; 14.67] | Bempedoic Acid 240 mg | . | . | . | -4.30 [-16.24; 7.64] |
| Colesevelam 4.5 g | -3.38 [-15.45; 8.69] | -8.37 [-21.48; 4.74] | -8.30 [-23.78; 7.18] | Colesevelam 4.5 g | . | . | 4.00 [ -5.85; 13.85] |
| Ezetimibe 10 mg | -0.28 [ -7.00; 6.44] | -5.27 [-13.49; 2.94] | -5.20 [-17.60; 7.20] | 3.10 [ -7.32; 13.51] | Ezetimibe 10 mg | . | 0.61 [ -2.85; 4.08] |
| Obicetrapib 10 mg | -50.93 [-60.72; -41.14] | -55.92 [-66.97; -44.87] | -55.85 [-69.63; -42.07] | -47.55 [-59.57; -35.53] | -50.65 [-58.31; -42.99] | Obicetrapib 10 mg | 51.55 [ 44.67; 58.43] |
| Placebo | 0.62 [ -6.34; 7.59] | -4.37 [-13.02; 4.27] | -4.30 [-16.24; 7.64] | 4.00 [ -5.85; 13.85] | 0.90 [ -2.46; 4.27] | 51.55 [ 44.67; 58.43] | Placebo |

Table S8.13: League table for percent APO A

|  | Bempedoic Acid 180 mg | Bempedoic Acid 180 mg + Ezetimibe 10 mg | Bempedoic Acid 240 mg | Colesevelam 3.75 g | Colesevelam 4.5 g | Ezetimibe 10 mg | Obicetrapib 10 mg | Obicetrapib 10 mg + Ezetimibe 10 mg | Placebo |
| --- | --- | --- | --- | --- | --- | --- | --- | --- | --- |
| Bempedoic Acid 180 mg | Bempedoic Acid 180 mg | 0.78 [0.32; 1.91] | . | . | . | 0.99 [0.06; 15.61] | . | . | 1.06 [0.90; 1.26] |
| Bempedoic Acid 180 mg + Ezetimibe 10 mg | 0.78 [0.41; 1.49] | Bempedoic Acid 180 mg + Ezetimibe 10 mg | . | . | . | 1.00 [0.43; 2.33] | . | . | . |
| Bempedoic Acid 240 mg | 1.04 [0.10; 10.79] | 1.33 [0.12; 15.02] | Bempedoic Acid 240 mg | . | . | . | . | . | 1.03 [0.10; 10.62] |
| Colesevelam 3.75 g | 0.67 [0.04; 10.89] | 0.86 [0.05; 14.64] | 0.65 [0.02; 24.49] | Colesevelam 3.75 g | . | 1.16 [0.07; 18.23] | . | . | . |
| Colesevelam 4.5 g | 1.12 [0.07; 17.78] | 1.44 [0.08; 24.45] | 1.08 [0.03; 40.14] | 1.67 [0.03; 83.44] | Colesevelam 4.5 g | . | . | . | 0.95 [0.06; 14.96] |
| Ezetimibe 10 mg | 0.78 [0.53; 1.14] | 1.00 [0.53; 1.90] | 0.75 [0.07; 7.99] | 1.16 [0.07; 18.23] | 0.70 [0.04; 11.21] | Ezetimibe 10 mg | . | 2.36 [0.63; 8.86] | 1.38 [0.95; 2.01] |
| Obicetrapib 10 mg | 1.16 [0.89; 1.51] | 1.49 [0.75; 2.93] | 1.12 [0.11; 11.66] | 1.72 [0.11; 27.88] | 1.03 [0.07; 16.41] | 1.49 [0.99; 2.22] | Obicetrapib 10 mg | 2.00 [0.51; 7.78] | 0.92 [0.75; 1.13] |
| Obicetrapib 10 mg + Ezetimibe 10 mg | 2.06 [0.77; 5.51] | 2.64 [0.85; 8.24] | 1.99 [0.16; 24.91] | 3.06 [0.16; 56.73] | 1.84 [0.10; 34.14] | 2.64 [1.00; 6.95] | 1.78 [0.67; 4.69] | Obicetrapib 10 mg + Ezetimibe 10 mg | . |
| Placebo | 1.07 [0.90; 1.26] | 1.37 [0.71; 2.61] | 1.03 [0.10; 10.62] | 1.58 [0.10; 25.44] | 0.95 [0.06; 14.96] | 1.36 [0.96; 1.94] | 0.92 [0.75; 1.13] | 0.52 [0.20; 1.37] | Placebo |

Table S8.14: League table for serious adverse events

|  | Bempedoic Acid 180 mg | Bempedoic Acid 180 mg + Ezetimibe 10 mg | Bempedoic Acid 240 mg | Colesevelam 3.75 g | Colesevelam 4.5 g | Ezetimibe 10 mg | Obicetrapib 10 mg | Obicetrapib 10 mg + Ezetimibe 10 mg | Placebo |
| --- | --- | --- | --- | --- | --- | --- | --- | --- | --- |
| Bempedoic Acid 180 mg | Bempedoic Acid 180 mg | 1.27 [0.54; 3.00] | . | . | . | 0.74 [0.27; 2.06] | . | . | 1.44 [1.15; 1.79] |
| Bempedoic Acid 180 mg + Ezetimibe 10 mg | 1.48 [0.79; 2.78] | Bempedoic Acid 180 mg + Ezetimibe 10 mg | . | . | . | 0.67 [0.29; 1.55] | . | . | . |
| Bempedoic Acid 240 mg | 1.63 [0.43; 6.20] | 1.10 [0.25; 4.74] | Bempedoic Acid 240 mg | . | . | . | . | . | 0.86 [0.23; 3.20] |
| Colesevelam 3.75 g | 0.31 [0.11; 0.85] | 0.21 [0.07; 0.65] | 0.19 [0.04; 0.99] | Colesevelam 3.75 g | . | 3.75 [1.45; 9.70] | . | . | . |
| Colesevelam 4.5 g | 0.98 [0.35; 2.70] | 0.66 [0.20; 2.14] | 0.60 [0.12; 3.14] | 3.18 [0.77; 13.08] | Colesevelam 4.5 g | . | . | . | 1.42 [0.53; 3.85] |
| Ezetimibe 10 mg | 1.15 [0.80; 1.67] | 0.78 [0.42; 1.45] | 0.71 [0.18; 2.76] | 3.75 [1.45; 9.70] | 1.18 [0.41; 3.35] | Ezetimibe 10 mg | . | 1.41 [0.46; 4.31] | 1.14 [0.79; 1.65] |
| Obicetrapib 10 mg | 1.53 [1.04; 2.26] | 1.03 [0.51; 2.09] | 0.94 [0.24; 3.66] | 4.97 [1.74; 14.26] | 1.56 [0.55; 4.45] | 1.33 [0.85; 2.08] | Obicetrapib 10 mg | 1.67 [0.62; 4.49] | 0.89 [0.64; 1.24] |
| Obicetrapib 10 mg + Ezetimibe 10 mg | 2.10 [0.94; 4.67] | 1.41 [0.53; 3.74] | 1.29 [0.28; 5.96] | 6.82 [1.99; 23.36] | 2.15 [0.61; 7.57] | 1.82 [0.83; 3.98] | 1.37 [0.64; 2.95] | Obicetrapib 10 mg + Ezetimibe 10 mg | . |
| Placebo | 1.39 [1.13; 1.72] | 0.94 [0.50; 1.77] | 0.86 [0.23; 3.20] | 4.53 [1.66; 12.39] | 1.42 [0.53; 3.85] | 1.21 [0.87; 1.68] | 0.91 [0.66; 1.26] | 0.66 [0.31; 1.44] | Placebo |

Table S8.15: League table for treatment discontinuation

|  | Bempedoic Acid 180 mg | Bempedoic Acid 180 mg + Ezetimibe 10 mg | Bempedoic Acid 240 mg | Colesevelam 3.75 g | Ezetimibe 10 mg | Obicetrapib 10 mg | Obicetrapib 10 mg + Ezetimibe 10 mg | placebo | Placebo |
| --- | --- | --- | --- | --- | --- | --- | --- | --- | --- |
| Bempedoic Acid 180 mg | Bempedoic Acid 180 mg | 1.33 [0.33; 5.34] | . | . | 0.17 [0.02; 1.35] | . | . | . | 0.94 [0.70; 1.25] |
| Bempedoic Acid 180 mg + Ezetimibe 10 mg | 1.22 [0.44; 3.37] | Bempedoic Acid 180 mg + Ezetimibe 10 mg | . | . | 0.84 [0.21; 3.47] | . | . | . | . |
| Bempedoic Acid 240 mg | 1.77 [0.12; 27.17] | 1.45 [0.08; 26.47] | Bempedoic Acid 240 mg | . | . | . | . | . | 0.51 [0.03; 7.76] |
| Colesevelam 3.75 g | 0.21 [0.02; 1.98] | 0.18 [0.02; 1.95] | 0.12 [0.00; 4.02] | Colesevelam 3.75 g | 4.36 [0.50; 38.27] | . | . | . | . |
| Ezetimibe 10 mg | 0.94 [0.59; 1.49] | 0.77 [0.28; 2.13] | 0.53 [0.03; 8.22] | 4.36 [0.50; 38.27] | Ezetimibe 10 mg | . | . | . | 0.92 [0.62; 1.36] |
| Obicetrapib 10 mg | 2.66 [0.28; 24.93] | 2.18 [0.19; 25.14] | 1.50 [0.05; 50.13] | 12.37 [0.54; 282.78] | 2.84 [0.30; 26.99] | Obicetrapib 10 mg | 1.03 [0.07; 15.83] | 0.50 [0.05; 5.30] | 0.34 [0.04; 3.15] |
| Obicetrapib 10 mg + Ezetimibe 10 mg | 2.73 [0.08; 93.55] | 2.24 [0.06; 87.80] | 1.54 [0.02; 131.77] | 12.69 [0.20; 810.56] | 2.91 [0.08; 100.74] | 1.03 [0.07; 15.83] | Obicetrapib 10 mg + Ezetimibe 10 mg | . | . |
| placebo | 1.33 [0.05; 34.38] | 1.09 [0.04; 32.63] | 0.75 [0.01; 51.50] | 6.19 [0.12; 311.61] | 1.42 [0.05; 37.05] | 0.50 [0.05; 5.30] | 0.49 [0.01; 18.09] | placebo | . |
| Placebo | 0.91 [0.68; 1.21] | 0.75 [0.27; 2.07] | 0.51 [0.03; 7.76] | 4.23 [0.47; 38.39] | 0.97 [0.66; 1.42] | 0.34 [0.04; 3.15] | 0.33 [0.01; 11.30] | 0.68 [0.03; 17.46] | Placebo |

Table S8.16: League table for myalgia

|  | Bempedoic Acid 180 mg | Bempedoic Acid 180 mg + Ezetimibe 10 mg | Bempedoic Acid 240 mg | Colesevelam 3.75 g | Colesevelam 4.5 g | Ezetimibe 10 mg | Obicetrapib 10 mg | Obicetrapib 10 mg + Ezetimibe 10 mg | Placebo |
| --- | --- | --- | --- | --- | --- | --- | --- | --- | --- |
| Bempedoic Acid 180 mg | Bempedoic Acid 180 mg | 1.00 [0.24; 4.14] | . | . | . | 0.97 [0.09; 10.07] | . | . | 0.91 [0.33; 2.52] |
| Bempedoic Acid 180 mg + Ezetimibe 10 mg | 1.20 [0.38; 3.77] | Bempedoic Acid 180 mg + Ezetimibe 10 mg | . | . | . | 0.60 [0.12; 3.07] | . | . | . |
| Bempedoic Acid 240 mg | 1.73 [0.14; 21.35] | 1.44 [0.10; 20.41] | Bempedoic Acid 240 mg | . | . | . | . | . | 0.48 [0.05; 5.13] |
| Colesevelam 3.75 g | 3.03 [0.27; 34.49] | 2.51 [0.20; 31.95] | 1.75 [0.06; 47.57] | Colesevelam 3.75 g | . | 0.31 [0.03; 2.89] | . | . | . |
| Colesevelam 4.5 g | 0.80 [0.22; 2.86] | 0.66 [0.14; 3.07] | 0.46 [0.04; 5.83] | 0.26 [0.02; 3.18] | Colesevelam 4.5 g | . | . | . | 1.05 [0.41; 2.69] |
| Ezetimibe 10 mg | 0.93 [0.36; 2.37] | 0.77 [0.23; 2.54] | 0.54 [0.05; 6.03] | 0.31 [0.03; 2.89] | 1.16 [0.39; 3.45] | Ezetimibe 10 mg | . | 0.99 [0.06; 15.62] | 0.85 [0.47; 1.52] |
| Obicetrapib 10 mg | 1.10 [0.41; 2.91] | 0.91 [0.25; 3.32] | 0.64 [0.06; 7.01] | 0.36 [0.03; 3.81] | 1.38 [0.49; 3.90] | 1.19 [0.59; 2.38] | Obicetrapib 10 mg | 0.27 [0.06; 1.28] | 0.78 [0.50; 1.22] |
| Obicetrapib 10 mg + Ezetimibe 10 mg | 0.39 [0.08; 2.01] | 0.32 [0.05; 2.03] | 0.22 [0.01; 3.51] | 0.13 [0.01; 1.87] | 0.49 [0.09; 2.66] | 0.42 [0.10; 1.81] | 0.35 [0.09; 1.40] | Obicetrapib 10 mg + Ezetimibe 10 mg | . |
| Placebo | 0.84 [0.35; 2.00] | 0.70 [0.21; 2.35] | 0.48 [0.05; 5.13] | 0.28 [0.03; 2.79] | 1.05 [0.41; 2.69] | 0.91 [0.52; 1.56] | 0.76 [0.49; 1.19] | 2.16 [0.53; 8.84] | Placebo |

Table S8.17: League table for headache

Table S8.18: League table for elevated liver enzymes

|  | Bempedoic Acid 180 mg | Bempedoic Acid 180 mg + Ezetimibe 10 mg | Ezetimibe 10 mg | Obicetrapib 10 mg | Obicetrapib 10 mg + Ezetimibe 10 mg | Placebo |
| --- | --- | --- | --- | --- | --- | --- |
| Bempedoic Acid 180 mg | Bempedoic Acid 180 mg | 0.36 [0.06; 2.15] | 0.50 [0.05; 5.37] | . | . | 1.27 [0.59; 2.75] |
| Bempedoic Acid 180 mg + Ezetimibe 10 mg | 0.48 [0.13; 1.76] | Bempedoic Acid 180 mg + Ezetimibe 10 mg | 2.04 [0.38; 10.92] | . | . | . |
| Ezetimibe 10 mg | 1.27 [0.56; 2.89] | 2.64 [0.73; 9.54] | Ezetimibe 10 mg | . | 2.02 [0.19; 21.93] | 0.81 [0.47; 1.39] |
| Obicetrapib 10 mg | 1.35 [0.45; 4.00] | 2.81 [0.60; 13.11] | 1.06 [0.41; 2.77] | Obicetrapib 10 mg | 3.00 [0.32; 28.36] | 0.79 [0.33; 1.85] |
| Obicetrapib 10 mg + Ezetimibe 10 mg | 3.26 [0.52; 20.55] | 6.79 [0.82; 56.37] | 2.57 [0.46; 14.25] | 2.42 [0.44; 13.20] | Obicetrapib 10 mg + Ezetimibe 10 mg | . |
| Placebo | 1.09 [0.54; 2.22] | 2.27 [0.62; 8.41] | 0.86 [0.51; 1.45] | 0.81 [0.35; 1.86] | 0.33 [0.06; 1.86] | Placebo |

**Supplement S9: Treatment ranking for each outcome**

Table S9.1: Treatment ranking for percent LDL-C

| **Rank** | **Intervention** | **P-score** |
| --- | --- | --- |
| First | Obicetrapib 10 mg + Ezetimibe 10 mg | 0.9941 |
| Second | Bempedoic Acid 180 mg + Ezetimibe 10 mg | 0.7943 |
| Third | Obicetrapib 10 mg | 0.7206 |
| Forth | Bempedoic Acid 240 mg | 0.5842 |
| Fifth | Bempedoic Acid 180 mg | 0.4000 |
| Sixth | Colesevelam 4.5 g | 0.3112 |
| Seventh | Ezetimibe 10 mg | 0.1951 |
| Eighth | Placebo | 0.0005 |

Table S9.2: Treatment ranking for absolute LDL-C

| **Rank** | **Intervention** | **P-score** |
| --- | --- | --- |
| First | Bempedoic Acid 240 mg | 0.8412 |
| Second | Bempedoic Acid 180 mg + Ezetimibe 10 mg | 0.8241 |
| Third | Obicetrapib 10 mg | 0.8023 |
| Forth | Bempedoic Acid 180 mg | 0.5496 |
| Fifth | Colesevelam 4.5 g | 0.5449 |
| Sixth | Ezetimibe 10 mg | 0.3580 |
| Seventh | Bempedoic acid 180 mg | 0.3065 |
| Eighth | Colesevelam 3.75 g | 0.2160 |
| Ninth | Placebo | 0.0575 |

Table S9.3: Treatment ranking for percent TC

| **Rank** | **Intervention** | **P-score** |
| --- | --- | --- |
| First | Bempedoic Acid 180 mg + Ezetimibe 10 mg | 0.9856 |
| Second | Bempedoic Acid 240 mg | 0.7951 |
| Third | Bempedoic Acid 180 mg | 0.6354 |
| Forth | Colesevelam 4.5 g | 0.5081 |
| Fifth | Ezetimibe 10 mg | 0.4084 |
| Sixth | Placebo | 0.1674 |
| Seventh | Obicetrapib 10 mg | 0.0000 |

Table S9.4: Treatment ranking for absolute TC

| **Rank** | **Intervention** | **P-score** |
| --- | --- | --- |
| First | Bempedoic Acid 240 mg | 0.8418 |
| Second | Bempedoic Acid 180 mg + Ezetimibe 10 mg | 0.7439 |
| Third | Colesevelam 4.5 g | 0.6362 |
| Forth | Bempedoic Acid 180 mg | 0.5575 |
| Fifth | Ezetimibe 10 mg | 0.4980 |
| Sixth | Placebo | 0.1687 |
| Seventh | Obicetrapib 10 mg | 0.0539 |

Table S9.5: Treatment ranking for percent HDL-C

| **Rank** | **Intervention** | **P-score** |
| --- | --- | --- |
| First | Bempedoic Acid 180 mg | 0.8410 |
| Second | Bempedoic Acid 180 mg + Ezetimibe 10 mg | 0.8191 |
| Third | Bempedoic Acid 240 mg | 0.8126 |
| Forth | Placebo | 0.5804 |
| Fifth | Colesevelam 4.5 g | 0.4061 |
| Sixth | Ezetimibe 10 mg | 0.3979 |
| Seventh | Obicetrapib 10 mg + Ezetimibe 10 mg | 0.1033 |
| Eighth | Obicetrapib 10 mg | 0.0396 |

Table S9.6: Treatment ranking for absolute HDL-C

| **Rank** | **Intervention** | **P-score** |
| --- | --- | --- |
| First | Placebo | 0.7853 |
| Second | Bempedoic Acid 240 mg | 0.7085 |
| Third | Bempedoic Acid 180 mg | 0.6502 |
| Forth | Ezetimibe 10 mg | 0.4812 |
| Fifth | Colesevelam 4.5 g | 0.4550 |
| Sixth | Colesevelam 3.5 g | 0.4199 |
| Seventh | Obicetrapib 10 mg | 0.0000 |

Table S9.7: Treatment ranking for percent Non-HDL-C

| **Rank** | **Intervention** | **P-score** |
| --- | --- | --- |
| First | Obicetrapib 10 mg + Ezetimibe 10 mg | 0.9938 |
| Second | Bempedoic Acid 180 mg + Ezetimibe 10 mg | 0.8186 |
| Third | Obicetrapib 10 mg | 0.6494 |
| Forth | Bempedoic Acid 240 mg | 0.4692 |
| Fifth | Bempedoic Acid 180 mg | 0.3392 |
| Sixth | Ezetimibe 10 mg | 0.2297 |
| Seventh | Placebo | 0.0001 |

Table S9.8: Treatment ranking for absolute Non-HDL-C

| **Rank** | **Intervention** | **P-score** |
| --- | --- | --- |
| First | Bempedoic Acid 240 mg | 0.9557 |
| Second | Ezetimibe 10 mg | 0.7139 |
| Third | Bempedoic Acid 180 mg | 0.4956 |
| Forth | Bempedoic Acid 180 mg + Ezetimibe 10 mg | 0.4503 |
| Fifth | Colesevelam 3.75 g | 0.3597 |
| Sixth | Placebo | 0.0248 |

Table S9.9: Treatment ranking for percent TG

| **Rank** | **Intervention** | **P-score** |
| --- | --- | --- |
| First | Bempedoic Acid 180 mg + Ezetimibe 10 mg | 0.7602 |
| Second | Ezetimibe 10 mg | 0.7557 |
| Third | Obicetrapib 10 mg + Ezetimibe 10 mg | 0.6818 |
| Forth | Obicetrapib 10 mg | 0.4559 |
| Fifth | Bempedoic Acid 180 mg | 0.4543 |
| Sixth | Placebo | 0.4248 |
| Seventh | Colesevelam 4.5 g | 0.3546 |
| Eighth | Bempedoic Acid 240 mg | 0.1127 |

Table S9.10: Treatment ranking for absolute TG

| **Rank** | **Intervention** | **P-score** |
| --- | --- | --- |
| First | Bempedoic Acid 180 mg + Ezetimibe 10 mg | 0.8791 |
| Second | Ezetimibe 10 mg | 0.6918 |
| Third | Obicetrapib 10 mg | 0.6733 |
| Forth | Placebo | 0.4172 |
| Fifth | Colesevelam 3.75 g | 0.3422 |
| Sixth | Bempedoic Acid 240 mg | 0.2768 |
| Seventh | Colesevelam 4.5 g | 0.2196 |

Table S9.11: Treatment ranking for percent APO B

| **Rank** | **Intervention** | **P-score** |
| --- | --- | --- |
| First | Obicetrapib 10 mg + Ezetimibe 10 mg | 0.9565 |
| Second | Bempedoic Acid 180 mg + Ezetimibe 10 mg | 0.8687 |
| Third | Obicetrapib 10 mg | 0.6954 |
| Forth | Bempedoic Acid 240 mg | 0.4414 |
| Fifth | Bempedoic Acid 180 mg | 0.4380 |
| Sixth | Colesevelam 4.5 g | 0.3170 |
| Seventh | Ezetimibe 10 mg | 0.2782 |
| Eighth | Placebo | 0.0049 |

Table S9.12: Treatment ranking for absolute APO B

| **Rank** | **Intervention** | **P-score** |
| --- | --- | --- |
| First | Obicetrapib 10 mg | 0.9975 |
| Second | Colesevelam 4.5 g | 0.6741 |
| Third | Bempedoic Acid 240 mg | 0.5962 |
| Forth | Bempedoic Acid 180 mg | 0.3704 |
| Fifth | Ezetimibe 10 mg | 0.3583 |
| Sixth | Placebo | 0.0034 |

Table S9.13: Treatment ranking for percent APO A

| **Rank** | **Intervention** | **P-score** |
| --- | --- | --- |
| First | Bempedoic Acid 180 mg + Ezetimibe 10 mg | 0.8363 |
| Second | Bempedoic Acid 240 mg | 0.7769 |
| Third | Placebo | 0.5763 |
| Forth | Bempedoic Acid 180 mg | 0.5048 |
| Fifth | Ezetimibe 10 mg | 0.4662 |
| Sixth | Colesevelam 4.5 g | 0.3395 |
| Seventh | Obicetrapib 10 mg | 0.0000 |

Table S9.14: Treatment ranking for serious adverse events

| **Rank** | **Intervention** | **P-score** |
| --- | --- | --- |
| First | Obicetrapib 10 mg + Ezetimibe 10 mg | 0.8465 |
| Second | Obicetrapib 10 mg | 0.6647 |
| Third | Placebo | 0.5596 |
| Forth | Colesevelam 4.5 g | 0.5248 |
| Fifth | Bempedoic Acid 240 mg | 0.5029 |
| Sixth | Bempedoic Acid 180 mg | 0.4602 |
| Seventh | Colesevelam 3.5 g | 0.3834 |
| Eighth | Bempedoic Acid 180 mg + Ezetimibe 10 mg | 0.3024 |
| Ninth | Ezetimibe 10 mg | 0.2555 |

Table S9.15: Treatment ranking for Treatment discontinuation

| **Rank** | **Intervention** | **P-score** |
| --- | --- | --- |
| First | Obicetrapib 10 mg + Ezetimibe 10 mg | 0.8506 |
| Second | Obicetrapib 10 mg | 0.6988 |
| Third | Bempedoic Acid 240 mg | 0.6505 |
| Forth | Bempedoic Acid 180 mg + Ezetimibe 10 mg | 0.6462 |
| Fifth | Placebo | 0.6119 |
| Sixth | Ezetimibe 10 mg | 0.4020 |
| Seventh | Colesevelam 4.5 g | 0.3607 |
| Eighth | Bempedoic Acid 180 mg | 0.2665 |
| Ninth | Colesevelam 3.5 g | 0.0127 |

Table S9.16: Treatment ranking for Myalgia

| **Rank** | **Intervention** | **P-score** |
| --- | --- | --- |
| First | Obicetrapib 10 mg | 0.7410 |
| Second | Obicetrapib 10 mg + Ezetimibe 10 mg | 0.6811 |
| Third | Bempedoic Acid 240 mg | 0.6110 |
| Forth | Bempedoic Acid 180 mg + Ezetimibe 10 mg | 0.5566 |
| Fifth | Placebo | 0.5200 |
| Sixth | Bempedoic Acid 180 mg | 0.4843 |
| Seventh | Ezetimibe 10 mg | 0.4212 |
| Eighth | Placebo | 0.3811 |
| Ninth | Colesevelam 3.5 g | 0.1038 |

Table S9.17: Treatment ranking for Headache

| **Rank** | **Intervention** | **P-score** |
| --- | --- | --- |
| First | Colesevelam 3.5 g | 0.8131 |
| Second | Bempedoic Acid 240 mg | 0.6607 |
| Third | Obicetrapib 10 mg | 0.6006 |
| Forth | Bempedoic Acid 180 mg + Ezetimibe 10 mg | 0.5985 |
| Fifth | Bempedoic Acid 180 mg | 0.5060 |
| Sixth | Ezetimibe 10 mg | 0.4582 |
| Seventh | Colesevelam 4.5 g | 0.3750 |
| Eighth | Placebo | 0.3640 |
| Ninth | Obicetrapib 10 mg + Ezetimibe 10 mg | 0.1238 |

Table S9.18: Treatment ranking for elevated liver enzymes

| **Rank** | **Intervention** | **P-score** |
| --- | --- | --- |
| First | Obicetrapib 10 mg + Ezetimibe 10 mg | 0.8918 |
| Second | Obicetrapib 10 mg | 0.6004 |
| Third | Ezetimibe 10 mg | 0.5898 |
| Forth | Placebo | 0.4374 |
| Fifth | Bempedoic Acid 180 mg | 0.3915 |
| Sixth | Bempedoic Acid 180 mg + Ezetimibe 10 mg | 0.0891 |

**Supplement S10: Certainty of evidence for each outcome**

Table S10.1: Percent LDL-C

| Comparison | Within_Study_Bias | Reporting_Bias | Indirectness | Imprecision | Heterogeneity | Incoherence | Overall_Confidence | Reason_for_downgrade |
| --- | --- | --- | --- | --- | --- | --- | --- | --- |
| Obicetrapib 10 mg:Placebo | Some concerns | No concerns | No concerns | Some concerns | Major concerns | No concerns | Low | Within-study bias, Imprecision, Heterogeneity |
| Obicetrapib 10 mg + Ezetimibe 10 mg:Placebo | Some concerns | No concerns | No concerns | Some concerns | Major concerns | No concerns | Low | Within-study bias, Imprecision, Heterogeneity |
| Bempedoic Acid 180 mg:Placebo | Major concerns | No concerns | Some concerns | Some concerns | Major concerns | No concerns | Very low | Within-study bias, Indirectness, Imprecision, Heterogeneity |
| Bempedoic Acid 180 mg + Ezetimibe 10 mg:Placebo | Major concerns | No concerns | Some concerns | Some concerns | Major concerns | No concerns | Very low | Within-study bias, Indirectness, Imprecision, Heterogeneity |
| Ezetimibe 10 mg:Placebo | Some concerns | No concerns | No concerns | Some concerns | Major concerns | No concerns | Low | Within-study bias, Imprecision, Heterogeneity |
| Colesevelam 4.5 g:Placebo | Major concerns | Some concerns | No concerns | Some concerns | Major concerns | No concerns | Very low | Within-study bias, Reporting bias, Imprecision, Heterogeneity |
| Bempedoic Acid 240 mg:Placebo | Major concerns | Some concerns | No concerns | Some concerns | Major concerns | No concerns | Very low | Within-study bias, Reporting bias, Imprecision, Heterogeneity |
| Obicetrapib 10 mg:Obicetrapib 10 mg + Ezetimibe 10 mg | Some concerns | No concerns | No concerns | Some concerns | Major concerns | No concerns | Low | Within-study bias, Imprecision, Heterogeneity |
| Bempedoic Acid 180 mg:Obicetrapib 10 mg | Some concerns | No concerns | Some concerns | Some concerns | Major concerns | No concerns | Low | Within-study bias, Indirectness, Imprecision, Heterogeneity |
| Bempedoic Acid 180 mg + Ezetimibe 10 mg:Obicetrapib 10 mg | Some concerns | No concerns | Some concerns | Major concerns | Major concerns | No concerns | Very low | Within-study bias, Indirectness, Imprecision, Heterogeneity |
| Ezetimibe 10 mg:Obicetrapib 10 mg | Some concerns | No concerns | No concerns | Some concerns | Major concerns | No concerns | Low | Within-study bias, Imprecision, Heterogeneity |
| Colesevelam 4.5 g:Obicetrapib 10 mg | Major concerns | No concerns | No concerns | Some concerns | Major concerns | No concerns | Very low | Within-study bias, Imprecision, Heterogeneity |
| Bempedoic Acid 240 mg:Obicetrapib 10 mg | Major concerns | No concerns | No concerns | Major concerns | Major concerns | No concerns | Very low | Within-study bias, Imprecision, Heterogeneity |
| Bempedoic Acid 180 mg:Obicetrapib 10 mg + Ezetimibe 10 mg | Some concerns | No concerns | Some concerns | Some concerns | Major concerns | No concerns | Low | Within-study bias, Indirectness, Imprecision, Heterogeneity |
| Bempedoic Acid 180 mg + Ezetimibe 10 mg:Obicetrapib 10 mg + Ezetimibe 10 mg | Some concerns | No concerns | Some concerns | Major concerns | Major concerns | No concerns | Very low | Within-study bias, Indirectness, Imprecision, Heterogeneity |
| Ezetimibe 10 mg:Obicetrapib 10 mg + Ezetimibe 10 mg | Some concerns | No concerns | Some concerns | Some concerns | Major concerns | No concerns | Low | Within-study bias, Indirectness, Imprecision, Heterogeneity |
| Colesevelam 4.5 g:Obicetrapib 10 mg + Ezetimibe 10 mg | Some concerns | No concerns | No concerns | Some concerns | Major concerns | No concerns | Low | Within-study bias, Imprecision, Heterogeneity |
| Bempedoic Acid 240 mg:Obicetrapib 10 mg + Ezetimibe 10 mg | Some concerns | No concerns | No concerns | Some concerns | Major concerns | No concerns | Low | Within-study bias, Imprecision, Heterogeneity |
| Bempedoic Acid 180 mg:Bempedoic Acid 180 mg + Ezetimibe 10 mg | Major concerns | No concerns | No concerns | Some concerns | Major concerns | No concerns | Very low | Within-study bias, Imprecision, Heterogeneity |
| Bempedoic Acid 180 mg:Ezetimibe 10 mg | Major concerns | No concerns | No concerns | Some concerns | Major concerns | No concerns | Very low | Within-study bias, Imprecision, Heterogeneity |
| Bempedoic Acid 180 mg:Colesevelam 4.5 g | Some concerns | No concerns | No concerns | Major concerns | Major concerns | No concerns | Very low | Within-study bias, Imprecision, Heterogeneity |
| Bempedoic Acid 180 mg:Bempedoic Acid 240 mg | Some concerns | No concerns | No concerns | Major concerns | Major concerns | No concerns | Very low | Within-study bias, Imprecision, Heterogeneity |
| Bempedoic Acid 180 mg + Ezetimibe 10 mg:Ezetimibe 10 mg | Major concerns | No concerns | No concerns | Some concerns | Major concerns | No concerns | Very low | Within-study bias, Imprecision, Heterogeneity |
| Bempedoic Acid 180 mg + Ezetimibe 10 mg:Colesevelam 4.5 g | Some concerns | No concerns | No concerns | Some concerns | Major concerns | No concerns | Low | Within-study bias, Imprecision, Heterogeneity |
| Bempedoic Acid 180 mg + Ezetimibe 10 mg:Bempedoic Acid 240 mg | Some concerns | No concerns | No concerns | Major concerns | Major concerns | No concerns | Very low | Within-study bias, Imprecision, Heterogeneity |
| Colesevelam 4.5 g:Ezetimibe 10 mg | Some concerns | No concerns | No concerns | Major concerns | Major concerns | No concerns | Very low | Within-study bias, Imprecision, Heterogeneity |
| Bempedoic Acid 240 mg:Ezetimibe 10 mg | Some concerns | No concerns | No concerns | Major concerns | Major concerns | No concerns | Very low | Within-study bias, Imprecision, Heterogeneity |
| Bempedoic Acid 240 mg:Colesevelam 4.5 g | Major concerns | Some concerns | No concerns | Major concerns | Major concerns | No concerns | Very low | Within-study bias, Reporting bias, Imprecision, Heterogeneity |

Table S10.2: Absolute LDL-C

| Comparison | Within_Study_Bias | Reporting_Bias | Indirectness | Imprecision | Heterogeneity | Incoherence | Overall_Confidence | Reason_for_downgrade |
| --- | --- | --- | --- | --- | --- | --- | --- | --- |
| Colesevelam 3.75 g:Placebo | Some concerns | No concerns | Some concerns | Major concerns | Major concerns | No concerns | Very low | Within-study bias, Indirectness, Imprecision, Heterogeneity |
| Bempedoic Acid 180 mg:Placebo | Some concerns | Some concerns | No concerns | Some concerns | Major concerns | No concerns | Low | Within-study bias, Reporting bias, Imprecision, Heterogeneity |
| Ezetimibe 10 mg:Placebo | Some concerns | No concerns | Some concerns | Some concerns | Major concerns | No concerns | Low | Within-study bias, Indirectness, Imprecision, Heterogeneity |
| Bempedoic acid 180 mg:Placebo | No concerns | Some concerns | Some concerns | Major concerns | Major concerns | No concerns | Very low | Reporting bias, Indirectness, Imprecision, Heterogeneity |
| Obicetrapib 10 mg:Placebo | Some concerns | Some concerns | No concerns | Some concerns | Major concerns | No concerns | Low | Within-study bias, Reporting bias, Imprecision, Heterogeneity |
| Colesevelam 4.5 g:Placebo | Major concerns | Some concerns | No concerns | Some concerns | Major concerns | No concerns | Very low | Within-study bias, Reporting bias, Imprecision, Heterogeneity |
| Bempedoic Acid 180 mg+Ezetimibe 10 mg:Placebo | Some concerns | No concerns | Some concerns | Some concerns | Major concerns | No concerns | Low | Within-study bias, Indirectness, Imprecision, Heterogeneity |
| Bempedoic Acid 240 mg:Placebo | Major concerns | Some concerns | No concerns | Some concerns | Major concerns | No concerns | Very low | Within-study bias, Reporting bias, Imprecision, Heterogeneity |
| Bempedoic Acid 180 mg:Colesevelam 3.75 g | Some concerns | No concerns | Some concerns | Major concerns | Major concerns | No concerns | Very low | Within-study bias, Indirectness, Imprecision, Heterogeneity |
| Colesevelam 3.75 g:Ezetimibe 10 mg | Major concerns | Some concerns | Some concerns | Major concerns | Major concerns | No concerns | Very low | Within-study bias, Reporting bias, Indirectness, Imprecision, Heterogeneity |
| Bempedoic acid 180 mg:Colesevelam 3.75 g | Some concerns | No concerns | Some concerns | Major concerns | Major concerns | No concerns | Very low | Within-study bias, Indirectness, Imprecision, Heterogeneity |
| Colesevelam 3.75 g:Obicetrapib 10 mg | Some concerns | No concerns | Some concerns | Major concerns | Major concerns | No concerns | Very low | Within-study bias, Indirectness, Imprecision, Heterogeneity |
| Colesevelam 3.75 g:Colesevelam 4.5 g | Major concerns | No concerns | Some concerns | Major concerns | Major concerns | No concerns | Very low | Within-study bias, Indirectness, Imprecision, Heterogeneity |
| Bempedoic Acid 180 mg+Ezetimibe 10 mg:Colesevelam 3.75 g | Major concerns | Some concerns | Some concerns | Major concerns | Major concerns | No concerns | Very low | Within-study bias, Reporting bias, Indirectness, Imprecision, Heterogeneity |
| Bempedoic Acid 240 mg:Colesevelam 3.75 g | Major concerns | No concerns | Some concerns | Major concerns | Major concerns | No concerns | Very low | Within-study bias, Indirectness, Imprecision, Heterogeneity |
| Bempedoic Acid 180 mg:Ezetimibe 10 mg | Some concerns | No concerns | Some concerns | Major concerns | Major concerns | No concerns | Very low | Within-study bias, Indirectness, Imprecision, Heterogeneity |
| Bempedoic acid 180 mg:Bempedoic Acid 180 mg | Some concerns | Some concerns | No concerns | Major concerns | Major concerns | No concerns | Very low | Within-study bias, Reporting bias, Imprecision, Heterogeneity |
| Bempedoic Acid 180 mg:Obicetrapib 10 mg | Some concerns | Some concerns | No concerns | Major concerns | Major concerns | No concerns | Very low | Within-study bias, Reporting bias, Imprecision, Heterogeneity |
| Bempedoic Acid 180 mg:Colesevelam 4.5 g | Some concerns | Some concerns | No concerns | Major concerns | Major concerns | No concerns | Very low | Within-study bias, Reporting bias, Imprecision, Heterogeneity |
| Bempedoic Acid 180 mg:Bempedoic Acid 180 mg+Ezetimibe 10 mg | Some concerns | No concerns | Some concerns | Major concerns | Major concerns | No concerns | Very low | Within-study bias, Indirectness, Imprecision, Heterogeneity |
| Bempedoic Acid 180 mg:Bempedoic Acid 240 mg | Some concerns | Some concerns | No concerns | Major concerns | Major concerns | No concerns | Very low | Within-study bias, Reporting bias, Imprecision, Heterogeneity |
| Bempedoic acid 180 mg:Ezetimibe 10 mg | Some concerns | No concerns | Some concerns | Major concerns | Major concerns | No concerns | Very low | Within-study bias, Indirectness, Imprecision, Heterogeneity |
| Ezetimibe 10 mg:Obicetrapib 10 mg | Some concerns | No concerns | Some concerns | Some concerns | Major concerns | No concerns | Low | Within-study bias, Indirectness, Imprecision, Heterogeneity |
| Colesevelam 4.5 g:Ezetimibe 10 mg | Some concerns | No concerns | Some concerns | Major concerns | Major concerns | No concerns | Very low | Within-study bias, Indirectness, Imprecision, Heterogeneity |
| Bempedoic Acid 180 mg+Ezetimibe 10 mg:Ezetimibe 10 mg | Major concerns | Some concerns | Some concerns | Some concerns | Major concerns | No concerns | Very low | Within-study bias, Reporting bias, Indirectness, Imprecision, Heterogeneity |
| Bempedoic Acid 240 mg:Ezetimibe 10 mg | Some concerns | No concerns | Some concerns | Major concerns | Major concerns | No concerns | Very low | Within-study bias, Indirectness, Imprecision, Heterogeneity |
| Bempedoic acid 180 mg:Obicetrapib 10 mg | Some concerns | Some concerns | Some concerns | Major concerns | Major concerns | No concerns | Very low | Within-study bias, Reporting bias, Indirectness, Imprecision, Heterogeneity |
| Bempedoic acid 180 mg:Colesevelam 4.5 g | Some concerns | Some concerns | No concerns | Major concerns | Major concerns | No concerns | Very low | Within-study bias, Reporting bias, Imprecision, Heterogeneity |
| Bempedoic acid 180 mg:Bempedoic Acid 180 mg+Ezetimibe 10 mg | Some concerns | No concerns | Some concerns | Major concerns | Major concerns | No concerns | Very low | Within-study bias, Indirectness, Imprecision, Heterogeneity |
| Bempedoic acid 180 mg:Bempedoic Acid 240 mg | Some concerns | Some concerns | No concerns | Major concerns | Major concerns | No concerns | Very low | Within-study bias, Reporting bias, Imprecision, Heterogeneity |
| Colesevelam 4.5 g:Obicetrapib 10 mg | Major concerns | Some concerns | No concerns | Major concerns | Major concerns | No concerns | Very low | Within-study bias, Reporting bias, Imprecision, Heterogeneity |
| Bempedoic Acid 180 mg+Ezetimibe 10 mg:Obicetrapib 10 mg | Some concerns | No concerns | Some concerns | Major concerns | Major concerns | No concerns | Very low | Within-study bias, Indirectness, Imprecision, Heterogeneity |
| Bempedoic Acid 240 mg:Obicetrapib 10 mg | Major concerns | Some concerns | No concerns | Major concerns | Major concerns | No concerns | Very low | Within-study bias, Reporting bias, Imprecision, Heterogeneity |
| Bempedoic Acid 180 mg+Ezetimibe 10 mg:Colesevelam 4.5 g | Major concerns | No concerns | Some concerns | Major concerns | Major concerns | No concerns | Very low | Within-study bias, Indirectness, Imprecision, Heterogeneity |
| Bempedoic Acid 240 mg:Colesevelam 4.5 g | Major concerns | Some concerns | No concerns | Major concerns | Major concerns | No concerns | Very low | Within-study bias, Reporting bias, Imprecision, Heterogeneity |
| Bempedoic Acid 180 mg+Ezetimibe 10 mg:Bempedoic Acid 240 mg | Major concerns | No concerns | Some concerns | Major concerns | Major concerns | No concerns | Very low | Within-study bias, Indirectness, Imprecision, Heterogeneity |

Table S10.3: Percent TC

| Comparison | Within_Study_Bias | Reporting_Bias | Indirectness | Imprecision | Heterogeneity | Incoherence | Overall_Confidence | Reason_for_downgrade |
| --- | --- | --- | --- | --- | --- | --- | --- | --- |
| Bempedoic Acid 180 mg:Placebo | Some concerns | No concerns | Some concerns | Some concerns | Major concerns | No concerns | Low | Within-study bias, Indirectness, Imprecision, Heterogeneity |
| Bempedoic Acid 180 mg + Ezetimibe 10 mg:Placebo | Some concerns | No concerns | Some concerns | Some concerns | Major concerns | No concerns | Low | Within-study bias, Indirectness, Imprecision, Heterogeneity |
| Obicetrapib 10 mg:Placebo | Some concerns | Some concerns | No concerns | Some concerns | Major concerns | No concerns | Low | Within-study bias, Reporting bias, Imprecision, Heterogeneity |
| Ezetimibe 10 mg:Placebo | Some concerns | No concerns | No concerns | Some concerns | Major concerns | No concerns | Low | Within-study bias, Imprecision, Heterogeneity |
| Colesevelam 4.5 g:Placebo | Major concerns | Some concerns | No concerns | Some concerns | Major concerns | No concerns | Very low | Within-study bias, Reporting bias, Imprecision, Heterogeneity |
| Bempedoic Acid 240 mg:Placebo | Major concerns | Some concerns | No concerns | Some concerns | Major concerns | No concerns | Very low | Within-study bias, Reporting bias, Imprecision, Heterogeneity |
| Bempedoic Acid 180 mg:Bempedoic Acid 180 mg + Ezetimibe 10 mg | Major concerns | No concerns | No concerns | Some concerns | Major concerns | No concerns | Very low | Within-study bias, Imprecision, Heterogeneity |
| Bempedoic Acid 180 mg:Obicetrapib 10 mg | Some concerns | No concerns | Some concerns | Some concerns | Major concerns | No concerns | Low | Within-study bias, Indirectness, Imprecision, Heterogeneity |
| Bempedoic Acid 180 mg:Ezetimibe 10 mg | Major concerns | No concerns | No concerns | Some concerns | Major concerns | No concerns | Very low | Within-study bias, Imprecision, Heterogeneity |
| Bempedoic Acid 180 mg:Colesevelam 4.5 g | Some concerns | No concerns | No concerns | Major concerns | Major concerns | No concerns | Very low | Within-study bias, Imprecision, Heterogeneity |
| Bempedoic Acid 180 mg:Bempedoic Acid 240 mg | Some concerns | No concerns | No concerns | Major concerns | Major concerns | No concerns | Very low | Within-study bias, Imprecision, Heterogeneity |
| Bempedoic Acid 180 mg + Ezetimibe 10 mg:Obicetrapib 10 mg | Some concerns | No concerns | Some concerns | Some concerns | Major concerns | No concerns | Low | Within-study bias, Indirectness, Imprecision, Heterogeneity |
| Bempedoic Acid 180 mg + Ezetimibe 10 mg:Ezetimibe 10 mg | Major concerns | No concerns | No concerns | Some concerns | Major concerns | No concerns | Very low | Within-study bias, Imprecision, Heterogeneity |
| Bempedoic Acid 180 mg + Ezetimibe 10 mg:Colesevelam 4.5 g | Major concerns | No concerns | Some concerns | Some concerns | Major concerns | No concerns | Very low | Within-study bias, Indirectness, Imprecision, Heterogeneity |
| Bempedoic Acid 180 mg + Ezetimibe 10 mg:Bempedoic Acid 240 mg | Major concerns | No concerns | Some concerns | Major concerns | Major concerns | No concerns | Very low | Within-study bias, Indirectness, Imprecision, Heterogeneity |
| Ezetimibe 10 mg:Obicetrapib 10 mg | Some concerns | No concerns | No concerns | Some concerns | Major concerns | No concerns | Low | Within-study bias, Imprecision, Heterogeneity |
| Colesevelam 4.5 g:Obicetrapib 10 mg | Major concerns | Some concerns | No concerns | Some concerns | Major concerns | No concerns | Very low | Within-study bias, Reporting bias, Imprecision, Heterogeneity |
| Bempedoic Acid 240 mg:Obicetrapib 10 mg | Major concerns | Some concerns | No concerns | Some concerns | Major concerns | No concerns | Very low | Within-study bias, Reporting bias, Imprecision, Heterogeneity |
| Colesevelam 4.5 g:Ezetimibe 10 mg | Major concerns | No concerns | Some concerns | Major concerns | Major concerns | No concerns | Very low | Within-study bias, Indirectness, Imprecision, Heterogeneity |
| Bempedoic Acid 240 mg:Ezetimibe 10 mg | Major concerns | No concerns | Some concerns | Major concerns | Major concerns | No concerns | Very low | Within-study bias, Indirectness, Imprecision, Heterogeneity |
| Bempedoic Acid 240 mg:Colesevelam 4.5 g | Major concerns | Some concerns | No concerns | Major concerns | Major concerns | No concerns | Very low | Within-study bias, Reporting bias, Imprecision, Heterogeneity |

Table S10.4: Absolute TC

| Comparison | Within_Study_Bias | Reporting_Bias | Indirectness | Imprecision | Heterogeneity | Incoherence | Overall_Confidence | Reason_for_downgrade |
| --- | --- | --- | --- | --- | --- | --- | --- | --- |
| Ezetimibe 10 mg:Placebo | Some concerns | Some concerns | Some concerns | Some concerns | Major concerns | No concerns | Low | Within-study bias, Reporting bias, Indirectness, Imprecision, Heterogeneity |
| Bempedoic acid 180 mg + Ezetimibe 10 mg:Placebo | Some concerns | Some concerns | Some concerns | Some concerns | Major concerns | No concerns | Low | Within-study bias, Reporting bias, Indirectness, Imprecision, Heterogeneity |
| Bempedoic Acid 180 mg:Placebo | Some concerns | Some concerns | Some concerns | Major concerns | Major concerns | No concerns | Very low | Within-study bias, Reporting bias, Indirectness, Imprecision, Heterogeneity |
| Obicetrapib 10 mg:Placebo | Major concerns | Some concerns | Some concerns | Major concerns | Major concerns | No concerns | Very low | Within-study bias, Reporting bias, Indirectness, Imprecision, Heterogeneity |
| Colesevelam 4.5 g:Placebo | Major concerns | Some concerns | No concerns | Major concerns | Major concerns | No concerns | Very low | Within-study bias, Reporting bias, Imprecision, Heterogeneity |
| Bempedoic Acid 240 mg:Placebo | Major concerns | Some concerns | No concerns | Some concerns | Major concerns | No concerns | Very low | Within-study bias, Reporting bias, Imprecision, Heterogeneity |
| Bempedoic acid 180 mg + Ezetimibe 10 mg:Ezetimibe 10 mg | Major concerns | Some concerns | Some concerns | Major concerns | Major concerns | No concerns | Very low | Within-study bias, Reporting bias, Indirectness, Imprecision, Heterogeneity |
| Bempedoic Acid 180 mg:Ezetimibe 10 mg | Some concerns | Some concerns | Some concerns | Major concerns | Major concerns | No concerns | Very low | Within-study bias, Reporting bias, Indirectness, Imprecision, Heterogeneity |
| Ezetimibe 10 mg:Obicetrapib 10 mg | Some concerns | Some concerns | Some concerns | Major concerns | Major concerns | No concerns | Very low | Within-study bias, Reporting bias, Indirectness, Imprecision, Heterogeneity |
| Colesevelam 4.5 g:Ezetimibe 10 mg | Some concerns | Some concerns | Some concerns | Major concerns | Major concerns | No concerns | Very low | Within-study bias, Reporting bias, Indirectness, Imprecision, Heterogeneity |
| Bempedoic Acid 240 mg:Ezetimibe 10 mg | Some concerns | Some concerns | Some concerns | Major concerns | Major concerns | No concerns | Very low | Within-study bias, Reporting bias, Indirectness, Imprecision, Heterogeneity |
| Bempedoic Acid 180 mg:Bempedoic acid 180 mg + Ezetimibe 10 mg | Some concerns | No concerns | Some concerns | Major concerns | Major concerns | No concerns | Very low | Within-study bias, Indirectness, Imprecision, Heterogeneity |
| Bempedoic acid 180 mg + Ezetimibe 10 mg:Obicetrapib 10 mg | Some concerns | No concerns | Some concerns | Some concerns | Major concerns | No concerns | Low | Within-study bias, Indirectness, Imprecision, Heterogeneity |
| Bempedoic acid 180 mg + Ezetimibe 10 mg:Colesevelam 4.5 g | Some concerns | No concerns | Some concerns | Major concerns | Major concerns | No concerns | Very low | Within-study bias, Indirectness, Imprecision, Heterogeneity |
| Bempedoic acid 180 mg + Ezetimibe 10 mg:Bempedoic Acid 240 mg | Some concerns | No concerns | Some concerns | Major concerns | Major concerns | No concerns | Very low | Within-study bias, Indirectness, Imprecision, Heterogeneity |
| Bempedoic Acid 180 mg:Obicetrapib 10 mg | Some concerns | Some concerns | Some concerns | Major concerns | Major concerns | No concerns | Very low | Within-study bias, Reporting bias, Indirectness, Imprecision, Heterogeneity |
| Bempedoic Acid 180 mg:Colesevelam 4.5 g | Some concerns | Some concerns | No concerns | Major concerns | Major concerns | No concerns | Very low | Within-study bias, Reporting bias, Imprecision, Heterogeneity |
| Bempedoic Acid 180 mg:Bempedoic Acid 240 mg | Some concerns | Some concerns | No concerns | Major concerns | Major concerns | No concerns | Very low | Within-study bias, Reporting bias, Imprecision, Heterogeneity |
| Colesevelam 4.5 g:Obicetrapib 10 mg | Major concerns | Some concerns | No concerns | Major concerns | Major concerns | No concerns | Very low | Within-study bias, Reporting bias, Imprecision, Heterogeneity |
| Bempedoic Acid 240 mg:Obicetrapib 10 mg | Major concerns | Some concerns | No concerns | Some concerns | Major concerns | No concerns | Very low | Within-study bias, Reporting bias, Imprecision, Heterogeneity |
| Bempedoic Acid 240 mg:Colesevelam 4.5 g | Major concerns | Some concerns | No concerns | Major concerns | Major concerns | No concerns | Very low | Within-study bias, Reporting bias, Imprecision, Heterogeneity |

Table S10.5: Percent HDL-C

| Comparison | Within_Study_Bias | Reporting_Bias | Indirectness | Imprecision | Heterogeneity | Incoherence | Overall_Confidence | Reason_for_downgrade |
| --- | --- | --- | --- | --- | --- | --- | --- | --- |
| Bempedoic Acid 180 mg:Placebo | Some concerns | No concerns | Some concerns | Some concerns | Major concerns | No concerns | Low | Within-study bias, Indirectness, Imprecision, Heterogeneity |
| Obicetrapib 10 mg:Placebo | Major concerns | Some concerns | No concerns | Some concerns | Major concerns | No concerns | Very low | Within-study bias, Reporting bias, Imprecision, Heterogeneity |
| Obicetrapib 10 mg + Ezetimibe 10 mg:Placebo | Major concerns | Some concerns | No concerns | Some concerns | Major concerns | No concerns | Very low | Within-study bias, Reporting bias, Imprecision, Heterogeneity |
| Ezetimibe 10 mg:Placebo | Some concerns | No concerns | No concerns | Major concerns | Major concerns | No concerns | Very low | Within-study bias, Imprecision, Heterogeneity |
| Colesevelam 4.5 g:Placebo | Major concerns | Some concerns | No concerns | Major concerns | Major concerns | No concerns | Very low | Within-study bias, Reporting bias, Imprecision, Heterogeneity |
| Bempedoic Acid 180 mg + Ezetimibe 10 mg:Placebo | Some concerns | No concerns | No concerns | Major concerns | Major concerns | No concerns | Very low | Within-study bias, Imprecision, Heterogeneity |
| Bempedoic Acid 240 mg:Placebo | Major concerns | Some concerns | No concerns | Major concerns | Major concerns | No concerns | Very low | Within-study bias, Reporting bias, Imprecision, Heterogeneity |
| Bempedoic Acid 180 mg:Obicetrapib 10 mg | Major concerns | No concerns | No concerns | Some concerns | Major concerns | No concerns | Very low | Within-study bias, Imprecision, Heterogeneity |
| Bempedoic Acid 180 mg:Obicetrapib 10 mg + Ezetimibe 10 mg | Major concerns | No concerns | No concerns | Some concerns | Major concerns | No concerns | Very low | Within-study bias, Imprecision, Heterogeneity |
| Bempedoic Acid 180 mg:Ezetimibe 10 mg | Major concerns | No concerns | No concerns | Some concerns | Major concerns | No concerns | Very low | Within-study bias, Imprecision, Heterogeneity |
| Bempedoic Acid 180 mg:Colesevelam 4.5 g | Some concerns | No concerns | No concerns | Major concerns | Major concerns | No concerns | Very low | Within-study bias, Imprecision, Heterogeneity |
| Bempedoic Acid 180 mg:Bempedoic Acid 180 mg + Ezetimibe 10 mg | Major concerns | No concerns | No concerns | Major concerns | Major concerns | No concerns | Very low | Within-study bias, Imprecision, Heterogeneity |
| Bempedoic Acid 180 mg:Bempedoic Acid 240 mg | Some concerns | No concerns | No concerns | Major concerns | Major concerns | No concerns | Very low | Within-study bias, Imprecision, Heterogeneity |
| Obicetrapib 10 mg:Obicetrapib 10 mg + Ezetimibe 10 mg | Major concerns | Some concerns | No concerns | Major concerns | Major concerns | No concerns | Very low | Within-study bias, Reporting bias, Imprecision, Heterogeneity |
| Ezetimibe 10 mg:Obicetrapib 10 mg | Major concerns | No concerns | No concerns | Some concerns | Major concerns | No concerns | Very low | Within-study bias, Imprecision, Heterogeneity |
| Colesevelam 4.5 g:Obicetrapib 10 mg | Major concerns | Some concerns | No concerns | Some concerns | Major concerns | No concerns | Very low | Within-study bias, Reporting bias, Imprecision, Heterogeneity |
| Bempedoic Acid 180 mg + Ezetimibe 10 mg:Obicetrapib 10 mg | Major concerns | No concerns | No concerns | Some concerns | Major concerns | No concerns | Very low | Within-study bias, Imprecision, Heterogeneity |
| Bempedoic Acid 240 mg:Obicetrapib 10 mg | Major concerns | Some concerns | No concerns | Some concerns | Major concerns | No concerns | Very low | Within-study bias, Reporting bias, Imprecision, Heterogeneity |
| Ezetimibe 10 mg:Obicetrapib 10 mg + Ezetimibe 10 mg | Major concerns | No concerns | No concerns | Some concerns | Major concerns | No concerns | Very low | Within-study bias, Imprecision, Heterogeneity |
| Colesevelam 4.5 g:Obicetrapib 10 mg + Ezetimibe 10 mg | Major concerns | Some concerns | No concerns | Some concerns | Major concerns | No concerns | Very low | Within-study bias, Reporting bias, Imprecision, Heterogeneity |
| Bempedoic Acid 180 mg + Ezetimibe 10 mg:Obicetrapib 10 mg + Ezetimibe 10 mg | Major concerns | No concerns | No concerns | Some concerns | Major concerns | No concerns | Very low | Within-study bias, Imprecision, Heterogeneity |
| Bempedoic Acid 240 mg:Obicetrapib 10 mg + Ezetimibe 10 mg | Major concerns | Some concerns | No concerns | Some concerns | Major concerns | No concerns | Very low | Within-study bias, Reporting bias, Imprecision, Heterogeneity |
| Colesevelam 4.5 g:Ezetimibe 10 mg | Major concerns | Some concerns | No concerns | Major concerns | Major concerns | No concerns | Very low | Within-study bias, Reporting bias, Imprecision, Heterogeneity |
| Bempedoic Acid 180 mg + Ezetimibe 10 mg:Ezetimibe 10 mg | Major concerns | No concerns | No concerns | Major concerns | Major concerns | No concerns | Very low | Within-study bias, Imprecision, Heterogeneity |
| Bempedoic Acid 240 mg:Ezetimibe 10 mg | Major concerns | Some concerns | No concerns | Major concerns | Major concerns | No concerns | Very low | Within-study bias, Reporting bias, Imprecision, Heterogeneity |
| Bempedoic Acid 180 mg + Ezetimibe 10 mg:Colesevelam 4.5 g | Major concerns | No concerns | No concerns | Major concerns | Major concerns | No concerns | Very low | Within-study bias, Imprecision, Heterogeneity |
| Bempedoic Acid 240 mg:Colesevelam 4.5 g | Major concerns | Some concerns | No concerns | Major concerns | Major concerns | No concerns | Very low | Within-study bias, Reporting bias, Imprecision, Heterogeneity |
| Bempedoic Acid 180 mg + Ezetimibe 10 mg:Bempedoic Acid 240 mg | Major concerns | Some concerns | No concerns | Major concerns | Major concerns | No concerns | Very low | Within-study bias, Reporting bias, Imprecision, Heterogeneity |

Table S10.6: Absolute HDL-C

| Comparison | Within_Study_Bias | Reporting_Bias | Indirectness | Imprecision | Heterogeneity | Incoherence | Overall_Confidence | Reason_for_downgrade |
| --- | --- | --- | --- | --- | --- | --- | --- | --- |
| Ezetimibe 10 mg:Placebo | Major concerns | No concerns | Some concerns | Major concerns | Major concerns | No concerns | Very low | Within-study bias, Indirectness, Imprecision, Heterogeneity |
| Bempedoic acid 180 mg:Placebo | Major concerns | Some concerns | Some concerns | Major concerns | Major concerns | No concerns | Very low | Within-study bias, Reporting bias, Indirectness, Imprecision, Heterogeneity |
| Obicetrapib 10 mg:Placebo | Major concerns | Some concerns | Some concerns | Some concerns | Major concerns | No concerns | Very low | Within-study bias, Reporting bias, Indirectness, Imprecision, Heterogeneity |
| Colesevelam 4.5 g:Placebo | Major concerns | Some concerns | No concerns | Major concerns | Major concerns | No concerns | Very low | Within-study bias, Reporting bias, Imprecision, Heterogeneity |
| Colesevelam 3.75 g:Placebo | Major concerns | Some concerns | Some concerns | Major concerns | Major concerns | No concerns | Very low | Within-study bias, Reporting bias, Indirectness, Imprecision, Heterogeneity |
| Bempedoic Acid 240 mg:Placebo | Major concerns | Some concerns | No concerns | Major concerns | Major concerns | No concerns | Very low | Within-study bias, Reporting bias, Imprecision, Heterogeneity |
| Bempedoic acid 180 mg:Ezetimibe 10 mg | Major concerns | No concerns | Some concerns | Major concerns | Major concerns | No concerns | Very low | Within-study bias, Indirectness, Imprecision, Heterogeneity |
| Ezetimibe 10 mg:Obicetrapib 10 mg | Major concerns | No concerns | Some concerns | Some concerns | Major concerns | No concerns | Very low | Within-study bias, Indirectness, Imprecision, Heterogeneity |
| Colesevelam 4.5 g:Ezetimibe 10 mg | Major concerns | No concerns | Some concerns | Major concerns | Major concerns | No concerns | Very low | Within-study bias, Indirectness, Imprecision, Heterogeneity |
| Colesevelam 3.75 g:Ezetimibe 10 mg | Major concerns | No concerns | Some concerns | Major concerns | Major concerns | No concerns | Very low | Within-study bias, Indirectness, Imprecision, Heterogeneity |
| Bempedoic Acid 240 mg:Ezetimibe 10 mg | Major concerns | No concerns | Some concerns | Major concerns | Major concerns | No concerns | Very low | Within-study bias, Indirectness, Imprecision, Heterogeneity |
| Bempedoic acid 180 mg:Obicetrapib 10 mg | Major concerns | Some concerns | Some concerns | Some concerns | Major concerns | No concerns | Very low | Within-study bias, Reporting bias, Indirectness, Imprecision, Heterogeneity |
| Bempedoic acid 180 mg:Colesevelam 4.5 g | Major concerns | Some concerns | No concerns | Major concerns | Major concerns | No concerns | Very low | Within-study bias, Reporting bias, Imprecision, Heterogeneity |
| Bempedoic acid 180 mg:Colesevelam 3.75 g | Major concerns | Some concerns | Some concerns | Major concerns | Major concerns | No concerns | Very low | Within-study bias, Reporting bias, Indirectness, Imprecision, Heterogeneity |
| Bempedoic acid 180 mg:Bempedoic Acid 240 mg | Major concerns | Some concerns | No concerns | Major concerns | Major concerns | No concerns | Very low | Within-study bias, Reporting bias, Imprecision, Heterogeneity |
| Colesevelam 4.5 g:Obicetrapib 10 mg | Major concerns | Some concerns | No concerns | Some concerns | Major concerns | No concerns | Very low | Within-study bias, Reporting bias, Imprecision, Heterogeneity |
| Colesevelam 3.75 g:Obicetrapib 10 mg | Major concerns | Some concerns | Some concerns | Some concerns | Major concerns | No concerns | Very low | Within-study bias, Reporting bias, Indirectness, Imprecision, Heterogeneity |
| Bempedoic Acid 240 mg:Obicetrapib 10 mg | Major concerns | Some concerns | No concerns | Some concerns | Major concerns | No concerns | Very low | Within-study bias, Reporting bias, Imprecision, Heterogeneity |
| Colesevelam 3.75 g:Colesevelam 4.5 g | Major concerns | Some concerns | No concerns | Major concerns | Major concerns | No concerns | Very low | Within-study bias, Reporting bias, Imprecision, Heterogeneity |
| Bempedoic Acid 240 mg:Colesevelam 4.5 g | Major concerns | Some concerns | No concerns | Major concerns | Major concerns | No concerns | Very low | Within-study bias, Reporting bias, Imprecision, Heterogeneity |
| Bempedoic Acid 240 mg:Colesevelam 3.75 g | Major concerns | Some concerns | No concerns | Major concerns | Major concerns | No concerns | Very low | Within-study bias, Reporting bias, Imprecision, Heterogeneity |

Table S10.7: Percent Non-HDL-C

| Comparison | Within_Study_Bias | Reporting_Bias | Indirectness | Imprecision | Heterogeneity | Incoherence | Overall_Confidence | Reason_for_downgrade |
| --- | --- | --- | --- | --- | --- | --- | --- | --- |
| Bempedoic Acid 180 mg:Placebo | Some concerns | No concerns | Some concerns | Some concerns | Major concerns | No concerns | Low | Within-study bias, Indirectness, Imprecision, Heterogeneity |
| Obicetrapib 10 mg:Placebo | Some concerns | No concerns | No concerns | Some concerns | Major concerns | No concerns | Low | Within-study bias, Imprecision, Heterogeneity |
| Obicetrapib 10 mg + Ezetimibe 10 mg:Placebo | Some concerns | No concerns | No concerns | Some concerns | Major concerns | No concerns | Low | Within-study bias, Imprecision, Heterogeneity |
| Bempedoic Acid 180 mg + Ezetimibe 10 mg:Placebo | Some concerns | No concerns | Some concerns | Some concerns | Major concerns | No concerns | Low | Within-study bias, Indirectness, Imprecision, Heterogeneity |
| Ezetimibe 10 mg:Placebo | Some concerns | No concerns | No concerns | Some concerns | Major concerns | No concerns | Low | Within-study bias, Imprecision, Heterogeneity |
| Bempedoic Acid 240 mg:Placebo | Major concerns | Some concerns | No concerns | Some concerns | Major concerns | No concerns | Very low | Within-study bias, Reporting bias, Imprecision, Heterogeneity |
| Bempedoic Acid 180 mg:Obicetrapib 10 mg | Some concerns | No concerns | No concerns | Some concerns | Major concerns | No concerns | Low | Within-study bias, Imprecision, Heterogeneity |
| Bempedoic Acid 180 mg:Obicetrapib 10 mg + Ezetimibe 10 mg | Some concerns | No concerns | No concerns | Some concerns | Major concerns | No concerns | Low | Within-study bias, Imprecision, Heterogeneity |
| Bempedoic Acid 180 mg:Bempedoic Acid 180 mg + Ezetimibe 10 mg | Major concerns | No concerns | No concerns | Some concerns | Major concerns | No concerns | Very low | Within-study bias, Imprecision, Heterogeneity |
| Bempedoic Acid 180 mg:Ezetimibe 10 mg | Major concerns | No concerns | No concerns | Major concerns | Major concerns | No concerns | Very low | Within-study bias, Imprecision, Heterogeneity |
| Bempedoic Acid 180 mg:Bempedoic Acid 240 mg | Some concerns | No concerns | Some concerns | Major concerns | Major concerns | No concerns | Very low | Within-study bias, Indirectness, Imprecision, Heterogeneity |
| Obicetrapib 10 mg:Obicetrapib 10 mg + Ezetimibe 10 mg | Some concerns | No concerns | No concerns | Some concerns | Major concerns | No concerns | Low | Within-study bias, Imprecision, Heterogeneity |
| Bempedoic Acid 180 mg + Ezetimibe 10 mg:Obicetrapib 10 mg | Some concerns | No concerns | Some concerns | Major concerns | Major concerns | No concerns | Very low | Within-study bias, Indirectness, Imprecision, Heterogeneity |
| Ezetimibe 10 mg:Obicetrapib 10 mg | Some concerns | No concerns | No concerns | Some concerns | Major concerns | No concerns | Low | Within-study bias, Imprecision, Heterogeneity |
| Bempedoic Acid 240 mg:Obicetrapib 10 mg | Some concerns | No concerns | Some concerns | Major concerns | Major concerns | No concerns | Very low | Within-study bias, Indirectness, Imprecision, Heterogeneity |
| Bempedoic Acid 180 mg + Ezetimibe 10 mg:Obicetrapib 10 mg + Ezetimibe 10 mg | Some concerns | No concerns | Some concerns | Major concerns | Major concerns | No concerns | Very low | Within-study bias, Indirectness, Imprecision, Heterogeneity |
| Ezetimibe 10 mg:Obicetrapib 10 mg + Ezetimibe 10 mg | Some concerns | No concerns | Some concerns | Some concerns | Major concerns | No concerns | Low | Within-study bias, Indirectness, Imprecision, Heterogeneity |
| Bempedoic Acid 240 mg:Obicetrapib 10 mg + Ezetimibe 10 mg | Some concerns | No concerns | Some concerns | Some concerns | Major concerns | No concerns | Low | Within-study bias, Indirectness, Imprecision, Heterogeneity |
| Bempedoic Acid 180 mg + Ezetimibe 10 mg:Ezetimibe 10 mg | Major concerns | No concerns | No concerns | Some concerns | Major concerns | No concerns | Very low | Within-study bias, Imprecision, Heterogeneity |
| Bempedoic Acid 180 mg + Ezetimibe 10 mg:Bempedoic Acid 240 mg | Major concerns | No concerns | Some concerns | Major concerns | Major concerns | No concerns | Very low | Within-study bias, Indirectness, Imprecision, Heterogeneity |
| Bempedoic Acid 240 mg:Ezetimibe 10 mg | Major concerns | No concerns | Some concerns | Major concerns | Major concerns | No concerns | Very low | Within-study bias, Indirectness, Imprecision, Heterogeneity |

Table S10.8: Absolute Non-HDL-C

| Comparison | Within_Study_Bias | Reporting_Bias | Indirectness | Imprecision | Heterogeneity | Incoherence | Overall_Confidence | Reason_for_downgrade |
| --- | --- | --- | --- | --- | --- | --- | --- | --- |
| Bempedoic acid 180 mg + Ezetimibe 10 mg:Placebo | Major concerns | Some concerns | Some concerns | Some concerns | Major concerns | No concerns | Very low | Within-study bias, Reporting bias, Indirectness, Imprecision, Heterogeneity |
| Ezetimibe 10 mg:Placebo | Major concerns | Some concerns | Some concerns | Some concerns | Major concerns | No concerns | Very low | Within-study bias, Reporting bias, Indirectness, Imprecision, Heterogeneity |
| Bempedoic Acid 180 mg:Placebo | Some concerns | Some concerns | Some concerns | Some concerns | Major concerns | No concerns | Low | Within-study bias, Reporting bias, Indirectness, Imprecision, Heterogeneity |
| Colesevelam 3.75 g:Placebo | Major concerns | Some concerns | Some concerns | Major concerns | Major concerns | No concerns | Very low | Within-study bias, Reporting bias, Indirectness, Imprecision, Heterogeneity |
| Bempedoic Acid 240 mg:Placebo | Major concerns | Some concerns | No concerns | Some concerns | Major concerns | No concerns | Very low | Within-study bias, Reporting bias, Imprecision, Heterogeneity |
| Bempedoic acid 180 mg + Ezetimibe 10 mg:Ezetimibe 10 mg | Major concerns | Some concerns | Some concerns | Major concerns | Major concerns | No concerns | Very low | Within-study bias, Reporting bias, Indirectness, Imprecision, Heterogeneity |
| Bempedoic Acid 180 mg:Bempedoic acid 180 mg + Ezetimibe 10 mg | Some concerns | Some concerns | Some concerns | Major concerns | Major concerns | No concerns | Very low | Within-study bias, Reporting bias, Indirectness, Imprecision, Heterogeneity |
| Bempedoic acid 180 mg + Ezetimibe 10 mg:Colesevelam 3.75 g | Major concerns | Some concerns | Some concerns | Major concerns | Major concerns | No concerns | Very low | Within-study bias, Reporting bias, Indirectness, Imprecision, Heterogeneity |
| Bempedoic acid 180 mg + Ezetimibe 10 mg:Bempedoic Acid 240 mg | Major concerns | Some concerns | No concerns | Major concerns | Major concerns | No concerns | Very low | Within-study bias, Reporting bias, Imprecision, Heterogeneity |
| Bempedoic Acid 180 mg:Ezetimibe 10 mg | Major concerns | Some concerns | Some concerns | Major concerns | Major concerns | No concerns | Very low | Within-study bias, Reporting bias, Indirectness, Imprecision, Heterogeneity |
| Colesevelam 3.75 g:Ezetimibe 10 mg | Major concerns | Some concerns | Some concerns | Major concerns | Major concerns | No concerns | Very low | Within-study bias, Reporting bias, Indirectness, Imprecision, Heterogeneity |
| Bempedoic Acid 240 mg:Ezetimibe 10 mg | Major concerns | Some concerns | Some concerns | Major concerns | Major concerns | No concerns | Very low | Within-study bias, Reporting bias, Indirectness, Imprecision, Heterogeneity |
| Bempedoic Acid 180 mg:Colesevelam 3.75 g | Major concerns | Some concerns | Some concerns | Major concerns | Major concerns | No concerns | Very low | Within-study bias, Reporting bias, Indirectness, Imprecision, Heterogeneity |
| Bempedoic Acid 180 mg:Bempedoic Acid 240 mg | Some concerns | Some concerns | No concerns | Major concerns | Major concerns | No concerns | Very low | Within-study bias, Reporting bias, Imprecision, Heterogeneity |
| Bempedoic Acid 240 mg:Colesevelam 3.75 g | Major concerns | Some concerns | Some concerns | Major concerns | Major concerns | No concerns | Very low | Within-study bias, Reporting bias, Indirectness, Imprecision, Heterogeneity |

Table S10.9: Percent TG

| Comparison | Within_Study_Bias | Reporting_Bias | Indirectness | Imprecision | Heterogeneity | Incoherence | Overall_Confidence | Reason_for_downgrade |
| --- | --- | --- | --- | --- | --- | --- | --- | --- |
| Bempedoic Acid 180 mg:Placebo | Some concerns | No concerns | Some concerns | Major concerns | Major concerns | No concerns | Very low | Within-study bias, Indirectness, Imprecision, Heterogeneity |
| Obicetrapib 10 mg:Placebo | Major concerns | Some concerns | No concerns | Major concerns | Major concerns | No concerns | Very low | Within-study bias, Reporting bias, Imprecision, Heterogeneity |
| Obicetrapib 10 mg + Ezetimibe 10 mg:Placebo | Major concerns | Some concerns | No concerns | Major concerns | Major concerns | No concerns | Very low | Within-study bias, Reporting bias, Imprecision, Heterogeneity |
| Ezetimibe 10 mg:Placebo | Some concerns | No concerns | No concerns | Some concerns | Major concerns | No concerns | Low | Within-study bias, Imprecision, Heterogeneity |
| Colesevelam 4.5 g:Placebo | Major concerns | Some concerns | No concerns | Major concerns | Major concerns | No concerns | Very low | Within-study bias, Reporting bias, Imprecision, Heterogeneity |
| Bempedoic Acid 180 mg + Ezetimibe 10 mg:Placebo | Some concerns | No concerns | No concerns | Major concerns | Major concerns | No concerns | Very low | Within-study bias, Imprecision, Heterogeneity |
| Bempedoic Acid 240 mg:Placebo | Major concerns | Some concerns | No concerns | Major concerns | Major concerns | No concerns | Very low | Within-study bias, Reporting bias, Imprecision, Heterogeneity |
| Bempedoic Acid 180 mg:Obicetrapib 10 mg | Major concerns | No concerns | No concerns | Major concerns | Major concerns | No concerns | Very low | Within-study bias, Imprecision, Heterogeneity |
| Bempedoic Acid 180 mg:Obicetrapib 10 mg + Ezetimibe 10 mg | Major concerns | No concerns | No concerns | Major concerns | Major concerns | No concerns | Very low | Within-study bias, Imprecision, Heterogeneity |
| Bempedoic Acid 180 mg:Ezetimibe 10 mg | Major concerns | No concerns | No concerns | Major concerns | Major concerns | No concerns | Very low | Within-study bias, Imprecision, Heterogeneity |
| Bempedoic Acid 180 mg:Colesevelam 4.5 g | Some concerns | No concerns | No concerns | Major concerns | Major concerns | No concerns | Very low | Within-study bias, Imprecision, Heterogeneity |
| Bempedoic Acid 180 mg:Bempedoic Acid 180 mg + Ezetimibe 10 mg | Major concerns | No concerns | No concerns | Major concerns | Major concerns | No concerns | Very low | Within-study bias, Imprecision, Heterogeneity |
| Bempedoic Acid 180 mg:Bempedoic Acid 240 mg | Some concerns | No concerns | No concerns | Major concerns | Major concerns | No concerns | Very low | Within-study bias, Imprecision, Heterogeneity |
| Obicetrapib 10 mg:Obicetrapib 10 mg + Ezetimibe 10 mg | Major concerns | Some concerns | No concerns | Major concerns | Major concerns | No concerns | Very low | Within-study bias, Reporting bias, Imprecision, Heterogeneity |
| Ezetimibe 10 mg:Obicetrapib 10 mg | Major concerns | No concerns | No concerns | Major concerns | Major concerns | No concerns | Very low | Within-study bias, Imprecision, Heterogeneity |
| Colesevelam 4.5 g:Obicetrapib 10 mg | Major concerns | Some concerns | No concerns | Major concerns | Major concerns | No concerns | Very low | Within-study bias, Reporting bias, Imprecision, Heterogeneity |
| Bempedoic Acid 180 mg + Ezetimibe 10 mg:Obicetrapib 10 mg | Major concerns | No concerns | No concerns | Major concerns | Major concerns | No concerns | Very low | Within-study bias, Imprecision, Heterogeneity |
| Bempedoic Acid 240 mg:Obicetrapib 10 mg | Major concerns | Some concerns | No concerns | Major concerns | Major concerns | No concerns | Very low | Within-study bias, Reporting bias, Imprecision, Heterogeneity |
| Ezetimibe 10 mg:Obicetrapib 10 mg + Ezetimibe 10 mg | Major concerns | No concerns | No concerns | Major concerns | Major concerns | No concerns | Very low | Within-study bias, Imprecision, Heterogeneity |
| Colesevelam 4.5 g:Obicetrapib 10 mg + Ezetimibe 10 mg | Major concerns | Some concerns | No concerns | Major concerns | Major concerns | No concerns | Very low | Within-study bias, Reporting bias, Imprecision, Heterogeneity |
| Bempedoic Acid 180 mg + Ezetimibe 10 mg:Obicetrapib 10 mg + Ezetimibe 10 mg | Major concerns | No concerns | No concerns | Major concerns | Major concerns | No concerns | Very low | Within-study bias, Imprecision, Heterogeneity |
| Bempedoic Acid 240 mg:Obicetrapib 10 mg + Ezetimibe 10 mg | Major concerns | Some concerns | No concerns | Major concerns | Major concerns | No concerns | Very low | Within-study bias, Reporting bias, Imprecision, Heterogeneity |
| Colesevelam 4.5 g:Ezetimibe 10 mg | Major concerns | Some concerns | No concerns | Major concerns | Major concerns | No concerns | Very low | Within-study bias, Reporting bias, Imprecision, Heterogeneity |
| Bempedoic Acid 180 mg + Ezetimibe 10 mg:Ezetimibe 10 mg | Major concerns | No concerns | No concerns | Major concerns | Major concerns | No concerns | Very low | Within-study bias, Imprecision, Heterogeneity |
| Bempedoic Acid 240 mg:Ezetimibe 10 mg | Major concerns | Some concerns | No concerns | Major concerns | Major concerns | No concerns | Very low | Within-study bias, Reporting bias, Imprecision, Heterogeneity |
| Bempedoic Acid 180 mg + Ezetimibe 10 mg:Colesevelam 4.5 g | Major concerns | No concerns | No concerns | Major concerns | Major concerns | No concerns | Very low | Within-study bias, Imprecision, Heterogeneity |
| Bempedoic Acid 240 mg:Colesevelam 4.5 g | Major concerns | Some concerns | No concerns | Major concerns | Major concerns | No concerns | Very low | Within-study bias, Reporting bias, Imprecision, Heterogeneity |
| Bempedoic Acid 180 mg + Ezetimibe 10 mg:Bempedoic Acid 240 mg | Major concerns | Some concerns | No concerns | Major concerns | Major concerns | No concerns | Very low | Within-study bias, Reporting bias, Imprecision, Heterogeneity |

Table S10.10: Absolute TG

| Comparison | Within_Study_Bias | Reporting_Bias | Indirectness | Imprecision | Heterogeneity | Incoherence | Overall_Confidence | Reason_for_downgrade |
| --- | --- | --- | --- | --- | --- | --- | --- | --- |
| Ezetimibe 10 mg:Placebo | Some concerns | No concerns | Some concerns | Some concerns | No concerns | No concerns | Low | Within-study bias, Indirectness, Imprecision |
| Bempedoic acid 180 mg + Ezetimibe 10 mg:Placebo | Some concerns | No concerns | Some concerns | Some concerns | No concerns | No concerns | Low | Within-study bias, Indirectness, Imprecision |
| Obicetrapib 10 mg:Placebo | Major concerns | Some concerns | Some concerns | Major concerns | No concerns | No concerns | Very low | Within-study bias, Reporting bias, Indirectness, Imprecision |
| Colesevelam 4.5 g:Placebo | Major concerns | Some concerns | No concerns | Major concerns | No concerns | No concerns | Very low | Within-study bias, Reporting bias, Imprecision |
| Colesevelam 3.75 g:Placebo | Some concerns | No concerns | Some concerns | Major concerns | No concerns | No concerns | Low | Within-study bias, Indirectness, Imprecision |
| Bempedoic Acid 240 mg:Placebo | Major concerns | Some concerns | No concerns | Major concerns | No concerns | No concerns | Very low | Within-study bias, Reporting bias, Imprecision |
| Bempedoic acid 180 mg + Ezetimibe 10 mg:Ezetimibe 10 mg | Major concerns | Some concerns | Some concerns | Some concerns | No concerns | No concerns | Low | Within-study bias, Reporting bias, Indirectness, Imprecision |
| Ezetimibe 10 mg:Obicetrapib 10 mg | Some concerns | No concerns | Some concerns | Major concerns | No concerns | No concerns | Low | Within-study bias, Indirectness, Imprecision |
| Colesevelam 4.5 g:Ezetimibe 10 mg | Some concerns | No concerns | Some concerns | Some concerns | No concerns | No concerns | Low | Within-study bias, Indirectness, Imprecision |
| Colesevelam 3.75 g:Ezetimibe 10 mg | Major concerns | Some concerns | Some concerns | Major concerns | No concerns | No concerns | Very low | Within-study bias, Reporting bias, Indirectness, Imprecision |
| Bempedoic Acid 240 mg:Ezetimibe 10 mg | Some concerns | No concerns | Some concerns | Major concerns | No concerns | No concerns | Low | Within-study bias, Indirectness, Imprecision |
| Bempedoic acid 180 mg + Ezetimibe 10 mg:Obicetrapib 10 mg | Some concerns | No concerns | Some concerns | Major concerns | No concerns | No concerns | Low | Within-study bias, Indirectness, Imprecision |
| Bempedoic acid 180 mg + Ezetimibe 10 mg:Colesevelam 4.5 g | Some concerns | No concerns | Some concerns | Some concerns | No concerns | No concerns | Low | Within-study bias, Indirectness, Imprecision |
| Bempedoic acid 180 mg + Ezetimibe 10 mg:Colesevelam 3.75 g | Major concerns | Some concerns | Some concerns | Major concerns | No concerns | No concerns | Very low | Within-study bias, Reporting bias, Indirectness, Imprecision |
| Bempedoic acid 180 mg + Ezetimibe 10 mg:Bempedoic Acid 240 mg | Some concerns | No concerns | Some concerns | Major concerns | No concerns | No concerns | Low | Within-study bias, Indirectness, Imprecision |
| Colesevelam 4.5 g:Obicetrapib 10 mg | Major concerns | Some concerns | No concerns | Major concerns | No concerns | No concerns | Very low | Within-study bias, Reporting bias, Imprecision |
| Colesevelam 3.75 g:Obicetrapib 10 mg | Some concerns | No concerns | Some concerns | Major concerns | No concerns | No concerns | Low | Within-study bias, Indirectness, Imprecision |
| Bempedoic Acid 240 mg:Obicetrapib 10 mg | Major concerns | Some concerns | No concerns | Major concerns | No concerns | No concerns | Very low | Within-study bias, Reporting bias, Imprecision |
| Colesevelam 3.75 g:Colesevelam 4.5 g | Some concerns | No concerns | Some concerns | Major concerns | No concerns | No concerns | Low | Within-study bias, Indirectness, Imprecision |
| Bempedoic Acid 240 mg:Colesevelam 4.5 g | Major concerns | Some concerns | No concerns | Major concerns | No concerns | No concerns | Very low | Within-study bias, Reporting bias, Imprecision |
| Bempedoic Acid 240 mg:Colesevelam 3.75 g | Some concerns | No concerns | Some concerns | Major concerns | No concerns | No concerns | Low | Within-study bias, Indirectness, Imprecision |

Table S10.11: Percent APO B

| Comparison | Within_Study_Bias | Reporting_Bias | Indirectness | Imprecision | Heterogeneity | Incoherence | Overall_Confidence | Reason_for_downgrade |
| --- | --- | --- | --- | --- | --- | --- | --- | --- |
| Bempedoic Acid 180 mg:Placebo | Some concerns | No concerns | Some concerns | Some concerns | Major concerns | No concerns | Low | Within-study bias, Indirectness, Imprecision, Heterogeneity |
| Obicetrapib 10 mg:Placebo | Some concerns | No concerns | No concerns | Some concerns | Major concerns | No concerns | Low | Within-study bias, Imprecision, Heterogeneity |
| Obicetrapib 10 mg + Ezetimibe 10 mg:Placebo | Some concerns | No concerns | No concerns | Some concerns | Major concerns | No concerns | Low | Within-study bias, Imprecision, Heterogeneity |
| Bempedoic Acid 180 mg + Ezetimibe 10 mg:Placebo | Some concerns | No concerns | Some concerns | Some concerns | Major concerns | No concerns | Low | Within-study bias, Indirectness, Imprecision, Heterogeneity |
| Ezetimibe 10 mg:Placebo | Some concerns | No concerns | No concerns | Some concerns | Major concerns | No concerns | Low | Within-study bias, Imprecision, Heterogeneity |
| Colesevelam 4.5 g:Placebo | Major concerns | Some concerns | No concerns | Some concerns | Major concerns | No concerns | Very low | Within-study bias, Reporting bias, Imprecision, Heterogeneity |
| Bempedoic Acid 240 mg:Placebo | Major concerns | Some concerns | No concerns | Some concerns | Major concerns | No concerns | Very low | Within-study bias, Reporting bias, Imprecision, Heterogeneity |
| Bempedoic Acid 180 mg:Obicetrapib 10 mg | Some concerns | No concerns | No concerns | Some concerns | Major concerns | No concerns | Low | Within-study bias, Imprecision, Heterogeneity |
| Bempedoic Acid 180 mg:Obicetrapib 10 mg + Ezetimibe 10 mg | Some concerns | No concerns | No concerns | Some concerns | Major concerns | No concerns | Low | Within-study bias, Imprecision, Heterogeneity |
| Bempedoic Acid 180 mg:Bempedoic Acid 180 mg + Ezetimibe 10 mg | Major concerns | No concerns | No concerns | Some concerns | Major concerns | No concerns | Very low | Within-study bias, Imprecision, Heterogeneity |
| Bempedoic Acid 180 mg:Ezetimibe 10 mg | Major concerns | No concerns | No concerns | Major concerns | Major concerns | No concerns | Very low | Within-study bias, Imprecision, Heterogeneity |
| Bempedoic Acid 180 mg:Colesevelam 4.5 g | Some concerns | No concerns | Some concerns | Major concerns | Major concerns | No concerns | Very low | Within-study bias, Indirectness, Imprecision, Heterogeneity |
| Bempedoic Acid 180 mg:Bempedoic Acid 240 mg | Some concerns | No concerns | Some concerns | Major concerns | Major concerns | No concerns | Very low | Within-study bias, Indirectness, Imprecision, Heterogeneity |
| Obicetrapib 10 mg:Obicetrapib 10 mg + Ezetimibe 10 mg | Some concerns | No concerns | No concerns | Some concerns | Major concerns | No concerns | Low | Within-study bias, Imprecision, Heterogeneity |
| Bempedoic Acid 180 mg + Ezetimibe 10 mg:Obicetrapib 10 mg | Some concerns | No concerns | Some concerns | Major concerns | Major concerns | No concerns | Very low | Within-study bias, Indirectness, Imprecision, Heterogeneity |
| Ezetimibe 10 mg:Obicetrapib 10 mg | Some concerns | No concerns | No concerns | Some concerns | Major concerns | No concerns | Low | Within-study bias, Imprecision, Heterogeneity |
| Colesevelam 4.5 g:Obicetrapib 10 mg | Some concerns | No concerns | Some concerns | Major concerns | Major concerns | No concerns | Very low | Within-study bias, Indirectness, Imprecision, Heterogeneity |
| Bempedoic Acid 240 mg:Obicetrapib 10 mg | Some concerns | No concerns | Some concerns | Major concerns | Major concerns | No concerns | Very low | Within-study bias, Indirectness, Imprecision, Heterogeneity |
| Bempedoic Acid 180 mg + Ezetimibe 10 mg:Obicetrapib 10 mg + Ezetimibe 10 mg | Some concerns | No concerns | Some concerns | Major concerns | Major concerns | No concerns | Very low | Within-study bias, Indirectness, Imprecision, Heterogeneity |
| Ezetimibe 10 mg:Obicetrapib 10 mg + Ezetimibe 10 mg | Some concerns | No concerns | Some concerns | Some concerns | Major concerns | No concerns | Low | Within-study bias, Indirectness, Imprecision, Heterogeneity |
| Colesevelam 4.5 g:Obicetrapib 10 mg + Ezetimibe 10 mg | Some concerns | No concerns | Some concerns | Some concerns | Major concerns | No concerns | Low | Within-study bias, Indirectness, Imprecision, Heterogeneity |
| Bempedoic Acid 240 mg:Obicetrapib 10 mg + Ezetimibe 10 mg | Some concerns | No concerns | Some concerns | Major concerns | Major concerns | No concerns | Very low | Within-study bias, Indirectness, Imprecision, Heterogeneity |
| Bempedoic Acid 180 mg + Ezetimibe 10 mg:Ezetimibe 10 mg | Major concerns | No concerns | No concerns | Some concerns | Major concerns | No concerns | Very low | Within-study bias, Imprecision, Heterogeneity |
| Bempedoic Acid 180 mg + Ezetimibe 10 mg:Colesevelam 4.5 g | Major concerns | No concerns | Some concerns | Some concerns | Major concerns | No concerns | Very low | Within-study bias, Indirectness, Imprecision, Heterogeneity |
| Bempedoic Acid 180 mg + Ezetimibe 10 mg:Bempedoic Acid 240 mg | Major concerns | No concerns | Some concerns | Major concerns | Major concerns | No concerns | Very low | Within-study bias, Indirectness, Imprecision, Heterogeneity |
| Colesevelam 4.5 g:Ezetimibe 10 mg | Major concerns | No concerns | Some concerns | Major concerns | Major concerns | No concerns | Very low | Within-study bias, Indirectness, Imprecision, Heterogeneity |
| Bempedoic Acid 240 mg:Ezetimibe 10 mg | Major concerns | No concerns | Some concerns | Major concerns | Major concerns | No concerns | Very low | Within-study bias, Indirectness, Imprecision, Heterogeneity |
| Bempedoic Acid 240 mg:Colesevelam 4.5 g | Major concerns | Some concerns | No concerns | Major concerns | Major concerns | No concerns | Very low | Within-study bias, Reporting bias, Imprecision, Heterogeneity |

Table S10.12: Absolute APO B

| Comparison | Within_Study_Bias | Reporting_Bias | Indirectness | Imprecision | Heterogeneity | Incoherence | Overall_Confidence | Reason_for_downgrade |
| --- | --- | --- | --- | --- | --- | --- | --- | --- |
| Ezetimibe 10 mg:Placebo | Some concerns | Some concerns | Some concerns | Some concerns | Major concerns | No concerns | Low | Within-study bias, Reporting bias, Indirectness, Imprecision, Heterogeneity |
| Bempedoic Acid 180 mg:Placebo | Some concerns | Some concerns | Some concerns | Some concerns | Major concerns | No concerns | Low | Within-study bias, Reporting bias, Indirectness, Imprecision, Heterogeneity |
| Obicetrapib 10 mg:Placebo | Major concerns | Some concerns | Some concerns | Some concerns | Major concerns | No concerns | Very low | Within-study bias, Reporting bias, Indirectness, Imprecision, Heterogeneity |
| Colesevelam 4.5 g:Placebo | Major concerns | Some concerns | No concerns | Some concerns | Major concerns | No concerns | Very low | Within-study bias, Reporting bias, Imprecision, Heterogeneity |
| Bempedoic Acid 240 mg:Placebo | Major concerns | Some concerns | No concerns | Some concerns | Major concerns | No concerns | Very low | Within-study bias, Reporting bias, Imprecision, Heterogeneity |
| Bempedoic Acid 180 mg:Ezetimibe 10 mg | Some concerns | Some concerns | Some concerns | Major concerns | Major concerns | No concerns | Very low | Within-study bias, Reporting bias, Indirectness, Imprecision, Heterogeneity |
| Ezetimibe 10 mg:Obicetrapib 10 mg | Major concerns | Some concerns | Some concerns | Some concerns | Major concerns | No concerns | Very low | Within-study bias, Reporting bias, Indirectness, Imprecision, Heterogeneity |
| Colesevelam 4.5 g:Ezetimibe 10 mg | Major concerns | Some concerns | Some concerns | Major concerns | Major concerns | No concerns | Very low | Within-study bias, Reporting bias, Indirectness, Imprecision, Heterogeneity |
| Bempedoic Acid 240 mg:Ezetimibe 10 mg | Major concerns | Some concerns | Some concerns | Major concerns | Major concerns | No concerns | Very low | Within-study bias, Reporting bias, Indirectness, Imprecision, Heterogeneity |
| Bempedoic Acid 180 mg:Obicetrapib 10 mg | Some concerns | Some concerns | Some concerns | Some concerns | Major concerns | No concerns | Low | Within-study bias, Reporting bias, Indirectness, Imprecision, Heterogeneity |
| Bempedoic Acid 180 mg:Colesevelam 4.5 g | Some concerns | Some concerns | No concerns | Major concerns | Major concerns | No concerns | Very low | Within-study bias, Reporting bias, Imprecision, Heterogeneity |
| Bempedoic Acid 180 mg:Bempedoic Acid 240 mg | Some concerns | Some concerns | No concerns | Major concerns | Major concerns | No concerns | Very low | Within-study bias, Reporting bias, Imprecision, Heterogeneity |
| Colesevelam 4.5 g:Obicetrapib 10 mg | Major concerns | Some concerns | No concerns | Some concerns | Major concerns | No concerns | Very low | Within-study bias, Reporting bias, Imprecision, Heterogeneity |
| Bempedoic Acid 240 mg:Obicetrapib 10 mg | Major concerns | Some concerns | No concerns | Some concerns | Major concerns | No concerns | Very low | Within-study bias, Reporting bias, Imprecision, Heterogeneity |
| Bempedoic Acid 240 mg:Colesevelam 4.5 g | Major concerns | Some concerns | No concerns | Major concerns | Major concerns | No concerns | Very low | Within-study bias, Reporting bias, Imprecision, Heterogeneity |

Table S10.13: Percent APO A

| Comparison | Within_Study_Bias | Reporting_Bias | Indirectness | Imprecision | Heterogeneity | Incoherence | Overall_Confidence | Reason_for_downgrade |
| --- | --- | --- | --- | --- | --- | --- | --- | --- |
| Bempedoic Acid 180 mg:Placebo | Major concerns | No concerns | Some concerns | Major concerns | Major concerns | No concerns | Very low | Within-study bias, Indirectness, Imprecision, Heterogeneity |
| Obicetrapib 10 mg:Placebo | Some concerns | Some concerns | No concerns | Some concerns | Major concerns | No concerns | Low | Within-study bias, Reporting bias, Imprecision, Heterogeneity |
| Ezetimibe 10 mg:Placebo | Some concerns | No concerns | No concerns | Major concerns | Major concerns | No concerns | Very low | Within-study bias, Imprecision, Heterogeneity |
| Colesevelam 4.5 g:Placebo | Major concerns | Some concerns | No concerns | Major concerns | Major concerns | No concerns | Very low | Within-study bias, Reporting bias, Imprecision, Heterogeneity |
| Bempedoic Acid 180 mg + Ezetimibe 10 mg:Placebo | Major concerns | No concerns | No concerns | Major concerns | Major concerns | No concerns | Very low | Within-study bias, Imprecision, Heterogeneity |
| Bempedoic Acid 240 mg:Placebo | Major concerns | Some concerns | No concerns | Major concerns | Major concerns | No concerns | Very low | Within-study bias, Reporting bias, Imprecision, Heterogeneity |
| Bempedoic Acid 180 mg:Obicetrapib 10 mg | Some concerns | No concerns | No concerns | Some concerns | Major concerns | No concerns | Low | Within-study bias, Imprecision, Heterogeneity |
| Bempedoic Acid 180 mg:Ezetimibe 10 mg | Major concerns | No concerns | No concerns | Major concerns | Major concerns | No concerns | Very low | Within-study bias, Imprecision, Heterogeneity |
| Bempedoic Acid 180 mg:Colesevelam 4.5 g | Major concerns | No concerns | No concerns | Major concerns | Major concerns | No concerns | Very low | Within-study bias, Imprecision, Heterogeneity |
| Bempedoic Acid 180 mg:Bempedoic Acid 180 mg + Ezetimibe 10 mg | Major concerns | No concerns | No concerns | Major concerns | Major concerns | No concerns | Very low | Within-study bias, Imprecision, Heterogeneity |
| Bempedoic Acid 180 mg:Bempedoic Acid 240 mg | Major concerns | No concerns | No concerns | Major concerns | Major concerns | No concerns | Very low | Within-study bias, Imprecision, Heterogeneity |
| Ezetimibe 10 mg:Obicetrapib 10 mg | Some concerns | No concerns | No concerns | Some concerns | Major concerns | No concerns | Low | Within-study bias, Imprecision, Heterogeneity |
| Colesevelam 4.5 g:Obicetrapib 10 mg | Major concerns | Some concerns | No concerns | Some concerns | Major concerns | No concerns | Very low | Within-study bias, Reporting bias, Imprecision, Heterogeneity |
| Bempedoic Acid 180 mg + Ezetimibe 10 mg:Obicetrapib 10 mg | Major concerns | No concerns | No concerns | Some concerns | Major concerns | No concerns | Very low | Within-study bias, Imprecision, Heterogeneity |
| Bempedoic Acid 240 mg:Obicetrapib 10 mg | Major concerns | Some concerns | No concerns | Some concerns | Major concerns | No concerns | Very low | Within-study bias, Reporting bias, Imprecision, Heterogeneity |
| Colesevelam 4.5 g:Ezetimibe 10 mg | Major concerns | Some concerns | No concerns | Major concerns | Major concerns | No concerns | Very low | Within-study bias, Reporting bias, Imprecision, Heterogeneity |
| Bempedoic Acid 180 mg + Ezetimibe 10 mg:Ezetimibe 10 mg | Major concerns | No concerns | No concerns | Major concerns | Major concerns | No concerns | Very low | Within-study bias, Imprecision, Heterogeneity |
| Bempedoic Acid 240 mg:Ezetimibe 10 mg | Major concerns | Some concerns | No concerns | Major concerns | Major concerns | No concerns | Very low | Within-study bias, Reporting bias, Imprecision, Heterogeneity |
| Bempedoic Acid 180 mg + Ezetimibe 10 mg:Colesevelam 4.5 g | Major concerns | No concerns | No concerns | Major concerns | Major concerns | No concerns | Very low | Within-study bias, Imprecision, Heterogeneity |
| Bempedoic Acid 240 mg:Colesevelam 4.5 g | Major concerns | Some concerns | No concerns | Major concerns | Major concerns | No concerns | Very low | Within-study bias, Reporting bias, Imprecision, Heterogeneity |
| Bempedoic Acid 180 mg + Ezetimibe 10 mg:Bempedoic Acid 240 mg | Major concerns | Some concerns | No concerns | Major concerns | Major concerns | No concerns | Very low | Within-study bias, Reporting bias, Imprecision, Heterogeneity |

Table S10.14: Serious adverse events

| Comparison | Within_Study_Bias | Reporting_Bias | Indirectness | Imprecision | Heterogeneity | Incoherence | Overall_Confidence | Reason_for_downgrade |
| --- | --- | --- | --- | --- | --- | --- | --- | --- |
| Colesevelam 3.75 g:Placebo | Some concerns | No concerns | Some concerns | Major concerns | No concerns | No concerns | Low | Within-study bias, Indirectness, Imprecision |
| Bempedoic Acid 180 mg:Placebo | Some concerns | No concerns | Some concerns | No concerns | No concerns | No concerns | Moderate | Within-study bias, Indirectness |
| Bempedoic Acid 180 mg + Ezetimibe 10 mg:Placebo | Some concerns | No concerns | Some concerns | No concerns | No concerns | No concerns | Moderate | Within-study bias, Indirectness |
| Ezetimibe 10 mg:Placebo | Some concerns | No concerns | Some concerns | No concerns | No concerns | No concerns | Moderate | Within-study bias, Indirectness |
| Obicetrapib 10 mg:Placebo | Some concerns | No concerns | No concerns | No concerns | No concerns | No concerns | Moderate | Within-study bias |
| Colesevelam 4.5 g:Placebo | Major concerns | Some concerns | No concerns | Major concerns | No concerns | No concerns | Very low | Within-study bias, Reporting bias, Imprecision |
| Obicetrapib 10 mg + Ezetimibe 10 mg:Placebo | Some concerns | No concerns | Some concerns | Major concerns | No concerns | No concerns | Low | Within-study bias, Indirectness, Imprecision |
| Bempedoic Acid 240 mg:Placebo | Major concerns | Some concerns | No concerns | Major concerns | No concerns | No concerns | Very low | Within-study bias, Reporting bias, Imprecision |
| Bempedoic Acid 180 mg:Colesevelam 3.75 g | Some concerns | No concerns | Some concerns | Major concerns | No concerns | No concerns | Low | Within-study bias, Indirectness, Imprecision |
| Bempedoic Acid 180 mg + Ezetimibe 10 mg:Colesevelam 3.75 g | Some concerns | No concerns | Some concerns | Major concerns | No concerns | No concerns | Low | Within-study bias, Indirectness, Imprecision |
| Colesevelam 3.75 g:Ezetimibe 10 mg | Major concerns | Some concerns | Some concerns | Major concerns | No concerns | No concerns | Very low | Within-study bias, Reporting bias, Indirectness, Imprecision |
| Colesevelam 3.75 g:Obicetrapib 10 mg | Some concerns | No concerns | Some concerns | Major concerns | No concerns | No concerns | Low | Within-study bias, Indirectness, Imprecision |
| Colesevelam 3.75 g:Colesevelam 4.5 g | Major concerns | No concerns | Some concerns | Major concerns | No concerns | No concerns | Very low | Within-study bias, Indirectness, Imprecision |
| Colesevelam 3.75 g:Obicetrapib 10 mg + Ezetimibe 10 mg | Some concerns | No concerns | Some concerns | Major concerns | No concerns | No concerns | Low | Within-study bias, Indirectness, Imprecision |
| Bempedoic Acid 240 mg:Colesevelam 3.75 g | Major concerns | No concerns | Some concerns | Major concerns | No concerns | No concerns | Very low | Within-study bias, Indirectness, Imprecision |
| Bempedoic Acid 180 mg:Bempedoic Acid 180 mg + Ezetimibe 10 mg | Major concerns | No concerns | Some concerns | No concerns | No concerns | No concerns | Low | Within-study bias, Indirectness |
| Bempedoic Acid 180 mg:Ezetimibe 10 mg | Major concerns | No concerns | No concerns | No concerns | No concerns | No concerns | Low | Within-study bias |
| Bempedoic Acid 180 mg:Obicetrapib 10 mg | Some concerns | No concerns | Some concerns | No concerns | No concerns | No concerns | Moderate | Within-study bias, Indirectness |
| Bempedoic Acid 180 mg:Colesevelam 4.5 g | Some concerns | No concerns | Some concerns | Major concerns | No concerns | No concerns | Low | Within-study bias, Indirectness, Imprecision |
| Bempedoic Acid 180 mg:Obicetrapib 10 mg + Ezetimibe 10 mg | Some concerns | No concerns | Some concerns | No concerns | No concerns | No concerns | Moderate | Within-study bias, Indirectness |
| Bempedoic Acid 180 mg:Bempedoic Acid 240 mg | Some concerns | No concerns | Some concerns | Major concerns | No concerns | No concerns | Low | Within-study bias, Indirectness, Imprecision |
| Bempedoic Acid 180 mg + Ezetimibe 10 mg:Ezetimibe 10 mg | Major concerns | No concerns | Some concerns | No concerns | No concerns | No concerns | Low | Within-study bias, Indirectness |
| Bempedoic Acid 180 mg + Ezetimibe 10 mg:Obicetrapib 10 mg | Some concerns | No concerns | Some concerns | No concerns | No concerns | No concerns | Moderate | Within-study bias, Indirectness |
| Bempedoic Acid 180 mg + Ezetimibe 10 mg:Colesevelam 4.5 g | Major concerns | No concerns | Some concerns | Major concerns | No concerns | No concerns | Very low | Within-study bias, Indirectness, Imprecision |
| Bempedoic Acid 180 mg + Ezetimibe 10 mg:Obicetrapib 10 mg + Ezetimibe 10 mg | Some concerns | No concerns | Some concerns | Some concerns | No concerns | No concerns | Low | Within-study bias, Indirectness, Imprecision |
| Bempedoic Acid 180 mg + Ezetimibe 10 mg:Bempedoic Acid 240 mg | Major concerns | No concerns | Some concerns | Major concerns | No concerns | No concerns | Very low | Within-study bias, Indirectness, Imprecision |
| Ezetimibe 10 mg:Obicetrapib 10 mg | Some concerns | No concerns | Some concerns | No concerns | No concerns | No concerns | Moderate | Within-study bias, Indirectness |
| Colesevelam 4.5 g:Ezetimibe 10 mg | Major concerns | No concerns | Some concerns | Major concerns | No concerns | No concerns | Very low | Within-study bias, Indirectness, Imprecision |
| Ezetimibe 10 mg:Obicetrapib 10 mg + Ezetimibe 10 mg | No concerns | No concerns | Some concerns | No concerns | No concerns | No concerns | Moderate | Indirectness |
| Bempedoic Acid 240 mg:Ezetimibe 10 mg | Major concerns | No concerns | Some concerns | Major concerns | No concerns | No concerns | Very low | Within-study bias, Indirectness, Imprecision |
| Colesevelam 4.5 g:Obicetrapib 10 mg | Some concerns | No concerns | Some concerns | Major concerns | No concerns | No concerns | Low | Within-study bias, Indirectness, Imprecision |
| Obicetrapib 10 mg:Obicetrapib 10 mg + Ezetimibe 10 mg | Some concerns | No concerns | Some concerns | No concerns | No concerns | No concerns | Moderate | Within-study bias, Indirectness |
| Bempedoic Acid 240 mg:Obicetrapib 10 mg | Some concerns | No concerns | Some concerns | Major concerns | No concerns | No concerns | Low | Within-study bias, Indirectness, Imprecision |
| Colesevelam 4.5 g:Obicetrapib 10 mg + Ezetimibe 10 mg | Some concerns | No concerns | Some concerns | Major concerns | No concerns | No concerns | Low | Within-study bias, Indirectness, Imprecision |
| Bempedoic Acid 240 mg:Colesevelam 4.5 g | Major concerns | Some concerns | No concerns | Major concerns | No concerns | No concerns | Very low | Within-study bias, Reporting bias, Imprecision |
| Bempedoic Acid 240 mg:Obicetrapib 10 mg + Ezetimibe 10 mg | Some concerns | No concerns | Some concerns | Major concerns | No concerns | No concerns | Low | Within-study bias, Indirectness, Imprecision |

Table S10.15: Treatment discontinuation due to adverse events

| Comparison | Within_Study_Bias | Reporting_Bias | Indirectness | Imprecision | Heterogeneity | Incoherence | Overall_Confidence | Reason_for_downgrade |
| --- | --- | --- | --- | --- | --- | --- | --- | --- |
| Colesevelam 3.75 g:Placebo | Some concerns | No concerns | No concerns | Some concerns | No concerns | No concerns | Moderate | Within-study bias, Imprecision |
| Ezetimibe 10 mg:Placebo | Some concerns | No concerns | No concerns | No concerns | No concerns | No concerns | Moderate | Within-study bias |
| Bempedoic Acid 180 mg:Placebo | Some concerns | No concerns | Some concerns | No concerns | No concerns | No concerns | Moderate | Within-study bias, Indirectness |
| Bempedoic Acid 180 mg + Ezetimibe 10 mg:Placebo | Some concerns | No concerns | No concerns | No concerns | No concerns | No concerns | Moderate | Within-study bias |
| Obicetrapib 10 mg:Placebo | Some concerns | No concerns | No concerns | No concerns | No concerns | No concerns | Moderate | Within-study bias |
| Obicetrapib 10 mg + Ezetimibe 10 mg:Placebo | Some concerns | No concerns | No concerns | Major concerns | No concerns | No concerns | Low | Within-study bias, Imprecision |
| Colesevelam 4.5 g:Placebo | Major concerns | Some concerns | No concerns | No concerns | No concerns | No concerns | Low | Within-study bias, Reporting bias |
| Bempedoic Acid 240 mg:Placebo | Major concerns | Some concerns | No concerns | Major concerns | No concerns | No concerns | Very low | Within-study bias, Reporting bias, Imprecision |
| Colesevelam 3.75 g:Ezetimibe 10 mg | Major concerns | Some concerns | Some concerns | No concerns | No concerns | No concerns | Low | Within-study bias, Reporting bias, Indirectness |
| Bempedoic Acid 180 mg:Colesevelam 3.75 g | Some concerns | No concerns | Some concerns | Major concerns | No concerns | No concerns | Low | Within-study bias, Indirectness, Imprecision |
| Bempedoic Acid 180 mg + Ezetimibe 10 mg:Colesevelam 3.75 g | Major concerns | No concerns | Some concerns | Major concerns | No concerns | No concerns | Very low | Within-study bias, Indirectness, Imprecision |
| Colesevelam 3.75 g:Obicetrapib 10 mg | Some concerns | No concerns | No concerns | Some concerns | No concerns | No concerns | Moderate | Within-study bias, Imprecision |
| Colesevelam 3.75 g:Obicetrapib 10 mg + Ezetimibe 10 mg | Some concerns | No concerns | Some concerns | Some concerns | No concerns | No concerns | Low | Within-study bias, Indirectness, Imprecision |
| Colesevelam 3.75 g:Colesevelam 4.5 g | Some concerns | No concerns | No concerns | Some concerns | No concerns | No concerns | Moderate | Within-study bias, Imprecision |
| Bempedoic Acid 240 mg:Colesevelam 3.75 g | Some concerns | No concerns | No concerns | Major concerns | No concerns | No concerns | Low | Within-study bias, Imprecision |
| Bempedoic Acid 180 mg:Ezetimibe 10 mg | Some concerns | No concerns | No concerns | No concerns | No concerns | No concerns | Moderate | Within-study bias |
| Bempedoic Acid 180 mg + Ezetimibe 10 mg:Ezetimibe 10 mg | Major concerns | No concerns | Some concerns | No concerns | No concerns | No concerns | Low | Within-study bias, Indirectness |
| Ezetimibe 10 mg:Obicetrapib 10 mg | Some concerns | No concerns | No concerns | No concerns | No concerns | No concerns | Moderate | Within-study bias |
| Ezetimibe 10 mg:Obicetrapib 10 mg + Ezetimibe 10 mg | Some concerns | No concerns | Some concerns | No concerns | No concerns | No concerns | Moderate | Within-study bias, Indirectness |
| Colesevelam 4.5 g:Ezetimibe 10 mg | Some concerns | No concerns | No concerns | Some concerns | No concerns | No concerns | Moderate | Within-study bias, Imprecision |
| Bempedoic Acid 240 mg:Ezetimibe 10 mg | Some concerns | No concerns | No concerns | Major concerns | No concerns | No concerns | Low | Within-study bias, Imprecision |
| Bempedoic Acid 180 mg:Bempedoic Acid 180 mg + Ezetimibe 10 mg | Major concerns | No concerns | No concerns | No concerns | No concerns | No concerns | Low | Within-study bias |
| Bempedoic Acid 180 mg:Obicetrapib 10 mg | Some concerns | No concerns | No concerns | No concerns | No concerns | No concerns | Moderate | Within-study bias |
| Bempedoic Acid 180 mg:Obicetrapib 10 mg + Ezetimibe 10 mg | Some concerns | No concerns | Some concerns | No concerns | No concerns | No concerns | Moderate | Within-study bias, Indirectness |
| Bempedoic Acid 180 mg:Colesevelam 4.5 g | Some concerns | No concerns | No concerns | Major concerns | No concerns | No concerns | Low | Within-study bias, Imprecision |
| Bempedoic Acid 180 mg:Bempedoic Acid 240 mg | Some concerns | No concerns | No concerns | Some concerns | No concerns | No concerns | Moderate | Within-study bias, Imprecision |
| Bempedoic Acid 180 mg + Ezetimibe 10 mg:Obicetrapib 10 mg | Some concerns | No concerns | No concerns | No concerns | No concerns | No concerns | Moderate | Within-study bias |
| Bempedoic Acid 180 mg + Ezetimibe 10 mg:Obicetrapib 10 mg + Ezetimibe 10 mg | Some concerns | No concerns | Some concerns | No concerns | No concerns | No concerns | Moderate | Within-study bias, Indirectness |
| Bempedoic Acid 180 mg + Ezetimibe 10 mg:Colesevelam 4.5 g | Some concerns | No concerns | No concerns | Major concerns | No concerns | No concerns | Low | Within-study bias, Imprecision |
| Bempedoic Acid 180 mg + Ezetimibe 10 mg:Bempedoic Acid 240 mg | Some concerns | No concerns | No concerns | Major concerns | No concerns | No concerns | Low | Within-study bias, Imprecision |
| Obicetrapib 10 mg:Obicetrapib 10 mg + Ezetimibe 10 mg | Some concerns | No concerns | No concerns | No concerns | No concerns | No concerns | Moderate | Within-study bias |
| Colesevelam 4.5 g:Obicetrapib 10 mg | Some concerns | No concerns | No concerns | Some concerns | No concerns | No concerns | Moderate | Within-study bias, Imprecision |
| Bempedoic Acid 240 mg:Obicetrapib 10 mg | Some concerns | No concerns | No concerns | Major concerns | No concerns | No concerns | Low | Within-study bias, Imprecision |
| Colesevelam 4.5 g:Obicetrapib 10 mg + Ezetimibe 10 mg | Some concerns | No concerns | No concerns | Some concerns | No concerns | No concerns | Moderate | Within-study bias, Imprecision |
| Bempedoic Acid 240 mg:Obicetrapib 10 mg + Ezetimibe 10 mg | Some concerns | No concerns | No concerns | Major concerns | No concerns | No concerns | Low | Within-study bias, Imprecision |
| Bempedoic Acid 240 mg:Colesevelam 4.5 g | Major concerns | Some concerns | No concerns | Major concerns | No concerns | No concerns | Very low | Within-study bias, Reporting bias, Imprecision |

Table S10.16: Myalgia

| Comparison | Within_Study_Bias | Reporting_Bias | Indirectness | Imprecision | Heterogeneity | Incoherence | Overall_Confidence | Reason_for_downgrade |
| --- | --- | --- | --- | --- | --- | --- | --- | --- |
| Colesevelam 3.75 g:Placebo | Some concerns | No concerns | No concerns | Some concerns | No concerns | No concerns | Moderate | Within-study bias, Imprecision |
| Obicetrapib 10 mg:Placebo | Major concerns | Some concerns | No concerns | Major concerns | No concerns | No concerns | Very low | Within-study bias, Reporting bias, Imprecision |
| Obicetrapib 10 mg + Ezetimibe 10 mg:Placebo | Major concerns | Some concerns | No concerns | Major concerns | No concerns | No concerns | Very low | Within-study bias, Reporting bias, Imprecision |
| Bempedoic Acid 180 mg:Placebo | Some concerns | No concerns | Some concerns | No concerns | No concerns | No concerns | Moderate | Within-study bias, Indirectness |
| Bempedoic Acid 180 mg + Ezetimibe 10 mg:Placebo | Some concerns | No concerns | Some concerns | Major concerns | No concerns | No concerns | Low | Within-study bias, Indirectness, Imprecision |
| Ezetimibe 10 mg:Placebo | Some concerns | No concerns | No concerns | No concerns | No concerns | No concerns | Moderate | Within-study bias |
| Bempedoic Acid 240 mg:Placebo | Major concerns | Some concerns | No concerns | Major concerns | No concerns | No concerns | Very low | Within-study bias, Reporting bias, Imprecision |
| placebo:Placebo | Major concerns | Some concerns | No concerns | Major concerns | No concerns | No concerns | Very low | Within-study bias, Reporting bias, Imprecision |
| Colesevelam 3.75 g:Obicetrapib 10 mg | Major concerns | No concerns | Some concerns | Some concerns | No concerns | No concerns | Low | Within-study bias, Indirectness, Imprecision |
| Colesevelam 3.75 g:Obicetrapib 10 mg + Ezetimibe 10 mg | Major concerns | No concerns | Some concerns | Major concerns | No concerns | No concerns | Very low | Within-study bias, Indirectness, Imprecision |
| Bempedoic Acid 180 mg:Colesevelam 3.75 g | Some concerns | No concerns | No concerns | Major concerns | No concerns | No concerns | Low | Within-study bias, Imprecision |
| Bempedoic Acid 180 mg + Ezetimibe 10 mg:Colesevelam 3.75 g | Some concerns | No concerns | No concerns | Major concerns | No concerns | No concerns | Low | Within-study bias, Imprecision |
| Colesevelam 3.75 g:Ezetimibe 10 mg | Major concerns | Some concerns | Some concerns | Some concerns | No concerns | No concerns | Low | Within-study bias, Reporting bias, Indirectness, Imprecision |
| Bempedoic Acid 240 mg:Colesevelam 3.75 g | Major concerns | No concerns | Some concerns | Major concerns | No concerns | No concerns | Very low | Within-study bias, Indirectness, Imprecision |
| Colesevelam 3.75 g:placebo | Major concerns | No concerns | Some concerns | Major concerns | No concerns | No concerns | Very low | Within-study bias, Indirectness, Imprecision |
| Obicetrapib 10 mg:Obicetrapib 10 mg + Ezetimibe 10 mg | Major concerns | Some concerns | No concerns | Major concerns | No concerns | No concerns | Very low | Within-study bias, Reporting bias, Imprecision |
| Bempedoic Acid 180 mg:Obicetrapib 10 mg | Some concerns | No concerns | Some concerns | Major concerns | No concerns | No concerns | Low | Within-study bias, Indirectness, Imprecision |
| Bempedoic Acid 180 mg + Ezetimibe 10 mg:Obicetrapib 10 mg | Some concerns | No concerns | No concerns | Major concerns | No concerns | No concerns | Low | Within-study bias, Imprecision |
| Ezetimibe 10 mg:Obicetrapib 10 mg | Some concerns | No concerns | No concerns | Major concerns | No concerns | No concerns | Low | Within-study bias, Imprecision |
| Bempedoic Acid 240 mg:Obicetrapib 10 mg | Major concerns | Some concerns | No concerns | Major concerns | No concerns | No concerns | Very low | Within-study bias, Reporting bias, Imprecision |
| Obicetrapib 10 mg:placebo | Major concerns | Some concerns | Some concerns | Major concerns | No concerns | No concerns | Very low | Within-study bias, Reporting bias, Indirectness, Imprecision |
| Bempedoic Acid 180 mg:Obicetrapib 10 mg + Ezetimibe 10 mg | Some concerns | No concerns | No concerns | Major concerns | No concerns | No concerns | Low | Within-study bias, Imprecision |
| Bempedoic Acid 180 mg + Ezetimibe 10 mg:Obicetrapib 10 mg + Ezetimibe 10 mg | Major concerns | No concerns | No concerns | Major concerns | No concerns | No concerns | Very low | Within-study bias, Imprecision |
| Ezetimibe 10 mg:Obicetrapib 10 mg + Ezetimibe 10 mg | Some concerns | No concerns | No concerns | Major concerns | No concerns | No concerns | Low | Within-study bias, Imprecision |
| Bempedoic Acid 240 mg:Obicetrapib 10 mg + Ezetimibe 10 mg | Major concerns | Some concerns | No concerns | Major concerns | No concerns | No concerns | Very low | Within-study bias, Reporting bias, Imprecision |
| Obicetrapib 10 mg + Ezetimibe 10 mg:placebo | Major concerns | Some concerns | No concerns | Major concerns | No concerns | No concerns | Very low | Within-study bias, Reporting bias, Imprecision |
| Bempedoic Acid 180 mg:Bempedoic Acid 180 mg + Ezetimibe 10 mg | Major concerns | No concerns | No concerns | Some concerns | No concerns | No concerns | Low | Within-study bias, Imprecision |
| Bempedoic Acid 180 mg:Ezetimibe 10 mg | Major concerns | No concerns | No concerns | No concerns | No concerns | No concerns | Low | Within-study bias |
| Bempedoic Acid 180 mg:Bempedoic Acid 240 mg | Some concerns | No concerns | No concerns | Major concerns | No concerns | No concerns | Low | Within-study bias, Imprecision |
| Bempedoic Acid 180 mg:placebo | Some concerns | No concerns | No concerns | Major concerns | No concerns | No concerns | Low | Within-study bias, Imprecision |
| Bempedoic Acid 180 mg + Ezetimibe 10 mg:Ezetimibe 10 mg | Major concerns | No concerns | No concerns | Major concerns | No concerns | No concerns | Very low | Within-study bias, Imprecision |
| Bempedoic Acid 180 mg + Ezetimibe 10 mg:Bempedoic Acid 240 mg | Major concerns | No concerns | Some concerns | Major concerns | No concerns | No concerns | Very low | Within-study bias, Indirectness, Imprecision |
| Bempedoic Acid 180 mg + Ezetimibe 10 mg:placebo | Major concerns | No concerns | Some concerns | Major concerns | No concerns | No concerns | Very low | Within-study bias, Indirectness, Imprecision |
| Bempedoic Acid 240 mg:Ezetimibe 10 mg | Major concerns | No concerns | Some concerns | Major concerns | No concerns | No concerns | Very low | Within-study bias, Indirectness, Imprecision |
| Ezetimibe 10 mg:placebo | Major concerns | No concerns | Some concerns | Major concerns | No concerns | No concerns | Very low | Within-study bias, Indirectness, Imprecision |
| Bempedoic Acid 240 mg:placebo | Major concerns | Some concerns | No concerns | Major concerns | No concerns | No concerns | Very low | Within-study bias, Reporting bias, Imprecision |

Table S10.17: Headache

| Comparison | Within_Study_Bias | Reporting_Bias | Indirectness | Imprecision | Heterogeneity | Incoherence | Overall_Confidence | Reason_for_downgrade |
| --- | --- | --- | --- | --- | --- | --- | --- | --- |
| Ezetimibe 10 mg:Placebo | Some concerns | No concerns | Some concerns | No concerns | No concerns | No concerns | Moderate | Within-study bias, Indirectness |
| Bempedoic Acid 180 mg:Placebo | Major concerns | No concerns | Some concerns | Major concerns | No concerns | No concerns | Very low | Within-study bias, Indirectness, Imprecision |
| Obicetrapib 10 mg:Placebo | Some concerns | No concerns | Some concerns | No concerns | No concerns | No concerns | Moderate | Within-study bias, Indirectness |
| Colesevelam 3.75 g:Placebo | Some concerns | Some concerns | Some concerns | Major concerns | No concerns | No concerns | Low | Within-study bias, Reporting bias, Indirectness, Imprecision |
| Bempedoic Acid 180 mg + Ezetimibe 10 mg:Placebo | Major concerns | Some concerns | Some concerns | Major concerns | No concerns | No concerns | Very low | Within-study bias, Reporting bias, Indirectness, Imprecision |
| Obicetrapib 10 mg + Ezetimibe 10 mg:Placebo | Some concerns | Some concerns | Some concerns | Some concerns | No concerns | No concerns | Low | Within-study bias, Reporting bias, Indirectness, Imprecision |
| Colesevelam 4.5 g:Placebo | Major concerns | Some concerns | No concerns | No concerns | No concerns | No concerns | Low | Within-study bias, Reporting bias |
| Bempedoic Acid 240 mg:Placebo | Major concerns | Some concerns | No concerns | Major concerns | No concerns | No concerns | Very low | Within-study bias, Reporting bias, Imprecision |
| Bempedoic Acid 180 mg:Ezetimibe 10 mg | Major concerns | No concerns | No concerns | Major concerns | No concerns | No concerns | Very low | Within-study bias, Imprecision |
| Ezetimibe 10 mg:Obicetrapib 10 mg | Some concerns | No concerns | Some concerns | No concerns | No concerns | No concerns | Moderate | Within-study bias, Indirectness |
| Colesevelam 3.75 g:Ezetimibe 10 mg | Major concerns | Some concerns | Some concerns | Major concerns | No concerns | No concerns | Very low | Within-study bias, Reporting bias, Indirectness, Imprecision |
| Bempedoic Acid 180 mg + Ezetimibe 10 mg:Ezetimibe 10 mg | Major concerns | No concerns | Some concerns | Major concerns | No concerns | No concerns | Very low | Within-study bias, Indirectness, Imprecision |
| Ezetimibe 10 mg:Obicetrapib 10 mg + Ezetimibe 10 mg | No concerns | No concerns | Some concerns | Major concerns | No concerns | No concerns | Low | Indirectness, Imprecision |
| Colesevelam 4.5 g:Ezetimibe 10 mg | Some concerns | Some concerns | No concerns | Some concerns | No concerns | No concerns | Low | Within-study bias, Reporting bias, Imprecision |
| Bempedoic Acid 240 mg:Ezetimibe 10 mg | Some concerns | Some concerns | No concerns | Major concerns | No concerns | No concerns | Low | Within-study bias, Reporting bias, Imprecision |
| Bempedoic Acid 180 mg:Obicetrapib 10 mg | Some concerns | No concerns | Some concerns | No concerns | No concerns | No concerns | Moderate | Within-study bias, Indirectness |
| Bempedoic Acid 180 mg:Colesevelam 3.75 g | Major concerns | Some concerns | Some concerns | Major concerns | No concerns | No concerns | Very low | Within-study bias, Reporting bias, Indirectness, Imprecision |
| Bempedoic Acid 180 mg:Bempedoic Acid 180 mg + Ezetimibe 10 mg | Major concerns | No concerns | Some concerns | Some concerns | No concerns | No concerns | Low | Within-study bias, Indirectness, Imprecision |
| Bempedoic Acid 180 mg:Obicetrapib 10 mg + Ezetimibe 10 mg | Some concerns | Some concerns | Some concerns | Major concerns | No concerns | No concerns | Low | Within-study bias, Reporting bias, Indirectness, Imprecision |
| Bempedoic Acid 180 mg:Colesevelam 4.5 g | Some concerns | Some concerns | No concerns | Major concerns | No concerns | No concerns | Low | Within-study bias, Reporting bias, Imprecision |
| Bempedoic Acid 180 mg:Bempedoic Acid 240 mg | Some concerns | Some concerns | No concerns | Major concerns | No concerns | No concerns | Low | Within-study bias, Reporting bias, Imprecision |
| Colesevelam 3.75 g:Obicetrapib 10 mg | Some concerns | No concerns | Some concerns | Major concerns | No concerns | No concerns | Low | Within-study bias, Indirectness, Imprecision |
| Bempedoic Acid 180 mg + Ezetimibe 10 mg:Obicetrapib 10 mg | Some concerns | No concerns | Some concerns | Major concerns | No concerns | No concerns | Low | Within-study bias, Indirectness, Imprecision |
| Obicetrapib 10 mg:Obicetrapib 10 mg + Ezetimibe 10 mg | Some concerns | No concerns | Some concerns | Major concerns | No concerns | No concerns | Low | Within-study bias, Indirectness, Imprecision |
| Colesevelam 4.5 g:Obicetrapib 10 mg | Some concerns | No concerns | Some concerns | Some concerns | No concerns | No concerns | Low | Within-study bias, Indirectness, Imprecision |
| Bempedoic Acid 240 mg:Obicetrapib 10 mg | Some concerns | No concerns | Some concerns | Major concerns | No concerns | No concerns | Low | Within-study bias, Indirectness, Imprecision |
| Bempedoic Acid 180 mg + Ezetimibe 10 mg:Colesevelam 3.75 g | Major concerns | No concerns | Some concerns | Major concerns | No concerns | No concerns | Very low | Within-study bias, Indirectness, Imprecision |
| Colesevelam 3.75 g:Obicetrapib 10 mg + Ezetimibe 10 mg | Some concerns | No concerns | Some concerns | Major concerns | No concerns | No concerns | Low | Within-study bias, Indirectness, Imprecision |
| Colesevelam 3.75 g:Colesevelam 4.5 g | Major concerns | Some concerns | Some concerns | Major concerns | No concerns | No concerns | Very low | Within-study bias, Reporting bias, Indirectness, Imprecision |
| Bempedoic Acid 240 mg:Colesevelam 3.75 g | Major concerns | Some concerns | Some concerns | Major concerns | No concerns | No concerns | Very low | Within-study bias, Reporting bias, Indirectness, Imprecision |
| Bempedoic Acid 180 mg + Ezetimibe 10 mg:Obicetrapib 10 mg + Ezetimibe 10 mg | Some concerns | No concerns | Some concerns | Major concerns | No concerns | No concerns | Low | Within-study bias, Indirectness, Imprecision |
| Bempedoic Acid 180 mg + Ezetimibe 10 mg:Colesevelam 4.5 g | Major concerns | Some concerns | No concerns | Major concerns | No concerns | No concerns | Very low | Within-study bias, Reporting bias, Imprecision |
| Bempedoic Acid 180 mg + Ezetimibe 10 mg:Bempedoic Acid 240 mg | Major concerns | Some concerns | No concerns | Major concerns | No concerns | No concerns | Very low | Within-study bias, Reporting bias, Imprecision |
| Colesevelam 4.5 g:Obicetrapib 10 mg + Ezetimibe 10 mg | Some concerns | Some concerns | Some concerns | Major concerns | No concerns | No concerns | Low | Within-study bias, Reporting bias, Indirectness, Imprecision |
| Bempedoic Acid 240 mg:Obicetrapib 10 mg + Ezetimibe 10 mg | Some concerns | Some concerns | Some concerns | Major concerns | No concerns | No concerns | Low | Within-study bias, Reporting bias, Indirectness, Imprecision |
| Bempedoic Acid 240 mg:Colesevelam 4.5 g | Major concerns | Some concerns | No concerns | Major concerns | No concerns | No concerns | Very low | Within-study bias, Reporting bias, Imprecision |

Table S10.18: Elevated liver enzymes

| Comparison | Within_Study_Bias | Reporting_Bias | Indirectness | Imprecision | Heterogeneity | Incoherence | Overall_Confidence | Reason_for_downgrade |
| --- | --- | --- | --- | --- | --- | --- | --- | --- |
| Bempedoic Acid 180 mg:Placebo | Some concerns | No concerns | Some concerns | No concerns | No concerns | No concerns | Moderate | Within-study bias, Indirectness |
| Bempedoic Acid 180 mg + Ezetimibe 10 mg:Placebo | Some concerns | No concerns | No concerns | Some concerns | No concerns | No concerns | Moderate | Within-study bias, Imprecision |
| Ezetimibe 10 mg:Placebo | Some concerns | No concerns | No concerns | No concerns | No concerns | No concerns | Moderate | Within-study bias |
| Obicetrapib 10 mg:Placebo | Some concerns | No concerns | No concerns | Major concerns | No concerns | No concerns | Low | Within-study bias, Imprecision |
| Obicetrapib 10 mg + Ezetimibe 10 mg:Placebo | Some concerns | No concerns | No concerns | Major concerns | No concerns | No concerns | Low | Within-study bias, Imprecision |
| Bempedoic Acid 180 mg:Bempedoic Acid 180 mg + Ezetimibe 10 mg | Major concerns | No concerns | No concerns | Major concerns | No concerns | No concerns | Very low | Within-study bias, Imprecision |
| Bempedoic Acid 180 mg:Ezetimibe 10 mg | Some concerns | No concerns | No concerns | No concerns | No concerns | No concerns | Moderate | Within-study bias |
| Bempedoic Acid 180 mg:Obicetrapib 10 mg | Some concerns | No concerns | No concerns | Some concerns | No concerns | No concerns | Moderate | Within-study bias, Imprecision |
| Bempedoic Acid 180 mg:Obicetrapib 10 mg + Ezetimibe 10 mg | Some concerns | No concerns | No concerns | Some concerns | No concerns | No concerns | Moderate | Within-study bias, Imprecision |
| Bempedoic Acid 180 mg + Ezetimibe 10 mg:Ezetimibe 10 mg | Major concerns | No concerns | No concerns | Some concerns | No concerns | No concerns | Low | Within-study bias, Imprecision |
| Bempedoic Acid 180 mg + Ezetimibe 10 mg:Obicetrapib 10 mg | Some concerns | No concerns | Some concerns | Some concerns | No concerns | No concerns | Low | Within-study bias, Indirectness, Imprecision |
| Bempedoic Acid 180 mg + Ezetimibe 10 mg:Obicetrapib 10 mg + Ezetimibe 10 mg | Some concerns | No concerns | No concerns | Some concerns | No concerns | No concerns | Moderate | Within-study bias, Imprecision |
| Ezetimibe 10 mg:Obicetrapib 10 mg | Some concerns | No concerns | Some concerns | No concerns | No concerns | No concerns | Moderate | Within-study bias, Indirectness |
| Ezetimibe 10 mg:Obicetrapib 10 mg + Ezetimibe 10 mg | No concerns | No concerns | Some concerns | Some concerns | No concerns | No concerns | Moderate | Indirectness, Imprecision |
| Obicetrapib 10 mg:Obicetrapib 10 mg + Ezetimibe 10 mg | No concerns | No concerns | Some concerns | Some concerns | No concerns | No concerns | Moderate | Indirectness, Imprecision |

| Outcome  **Supplement S11:** Sensitivity analysis  Table S11.1 leave one out analysis for Percent LDL-C | Study Omitted | I^2 | tau^2 | Interpretation |
| --- | --- | --- | --- | --- |
| Percent LDL-C | Harada-Shiba,2024 | 90.5% | 41.9791 | Effect stable |
| Percent LDL-C | Ballantyne,2023 | 90.5% | 42.0008 | Effect stable |
| Percent LDL-C | Sarraju,2025 | 90.4% | 41.7763 | Effect stable |
| Percent LDL-C | Laufs,2019 | 90.5% | 43.3490 | Effect stable |
| Percent LDL-C | Ballantyne,2020 | 90.5% | 43.0724 | Effect stable |
| Percent LDL-C | Ballantyne,2016 | 90.5% | 41.9407 | Effect stable |
| Percent LDL-C | Ray,2019 | 90.3% | 44.8687 | Effect stable |
| Percent LDL-C | Hovingh,2015 | 88.2% | 34.0892 | Effect stable |
| Percent LDL-C | Yang.2017 | 90.4% | 41.6290 | Effect stable |
| Percent LDL-C | Nicholls,2017 | 87.6% | 32.9440 | Effect stable |
| Percent LDL-C | Sasaki,2012 | 90.4% | 42.3346 | Effect stable |
| Percent LDL-C | insull,2001 | 90.1% | 41.5633 | Effect stable |
| Percent LDL-C | Sakamoto,2017 | 90.4% | 41.8591 | Effect stable |
| Percent LDL-C | Lee,2021 | 90.5% | 42.5031 | Effect stable |
| Percent LDL-C | Ballantyne,2018 | 90.1% | 41.0371 | Effect stable |
| Percent LDL-C | Yamashita,2025 | 90.4% | 42.8651 | Effect stable |
| Percent LDL-C | Gagne´,2002 | 88% | 37.4922 | Effect stable |
| Percent LDL-C | ballantyne,2004_a | 89.1% | 39.6495 | Effect stable |
| Percent LDL-C | GOLDBERG,2004 | 90.5% | 46.5220 | Effect stable |
| Percent LDL-C | Farnier,2005 | 90.5% | 42.7983 | Effect stable |
| Percent LDL-C | Qian,2022_a | 90.4% | 41.9047 | Effect stable |
| Percent LDL-C | Qian,2022_b | 90.5% | 42.6437 | Effect stable |
| Percent LDL-C | Stein,2008 | 90.5% | 42.7872 | Effect stable |
| Percent LDL-C | Torimoto,2013 | 90.4% | 42.0134 | Effect stable |
| Percent LDL-C | Yu,2012 | 88.7% | 40.6051 | Effect stable |
| Percent LDL-C | ballantyne,2004_b | 90.3% | 42.4596 | Effect stable |
| Percent LDL-C | Ward,2022 | 90.4% | 41.6620 | Effect stable |
| Percent LDL-C | Nicholls,2025 | 88.7% | 35.6290 | Effect stable |
| Percent LDL-C | Moon,2023 | 90.4% | 41.8295 | Effect stable |
| Percent LDL-C | Thompson,2016 | 90.4% | 43.4154 | Effect stable |
| Percent LDL-C | Thompson,2015 | 90.1% | 41.5633 | Effect stable |

Table S11.2 Leave one out analysis for Absolute LDL-C

| Outcome | Study Omitted | I^2 | tau^2 | Interpretation |
| --- | --- | --- | --- | --- |
| Absolute LDL-C | Bajaj,2020 | 92.2% | 182.9115 | Effect stable |
| Absolute LDL-C | Ballantyne,2018 | 91.4% | 196.4681 | Effect stable |
| Absolute LDL-C | Efrati,2007 | 92.6% | 183.3833 | Effect stable |
| Absolute LDL-C | goldberg,2019 | 92.2% | 182.9115 | Effect stable |
| Absolute LDL-C | Hovingh,2015 | 91.3% | 164.0023 | Effect stable |
| Absolute LDL-C | insull,2001 | 92.2% | 182.9115 | Effect stable |
| Absolute LDL-C | Lee,2021 | 88.3% | 117.3416 | Effect stable |
| Absolute LDL-C | Luo,2016 | 92.5% | 204.5801 | Effect stable |
| Absolute LDL-C | Marrazi,2024 | 92.2% | 182.9115 | Effect stable |
| Absolute LDL-C | Moreira,2014 | 92.4% | 181.3716 | Effect stable |
| Absolute LDL-C | Nicholls,2025 | 91.3% | 164.0023 | Effect stable |
| Absolute LDL-C | Qian,2022_a | 92.7% | 202.6425 | Effect stable |
| Absolute LDL-C | Ray,2019 | 91.4% | 200.8674 | Effect stable |
| Absolute LDL-C | RUGGENENTI,2010 | 92.1% | 175.7305 | Effect stable |
| Absolute LDL-C | Stein,2004 | 92.7% | 251.4362 | Effect stable |
| Absolute LDL-C | Stein,2007 | 92.1% | 173.2805 | Effect stable |
| Absolute LDL-C | Stein,2008 | 92.7% | 185.4360 | Effect stable |
| Absolute LDL-C | Teramoto,2012 | 92.6% | 181.5022 | Effect stable |
| Absolute LDL-C | Thompson,2015 | 92.2% | 182.9115 | Effect stable |
| Absolute LDL-C | Torimoto,2013 | 92.6% | 191.4612 | Effect stable |
| Absolute LDL-C | Ward,2022 | 92.6% | 182.0248 | Effect stable |
| Absolute LDL-C | Yamashita,2025 | 92.7% | 198.3779 | Effect stable |
| Absolute LDL-C | Yang,2017 | 92.7% | 189.4333 | Effect stable |

Table S11.3 leave one out analysis for Percent TC

| Outcome | Study Omitted | I^2 | tau^2 | Interpretation |
| --- | --- | --- | --- | --- |
| Percent TC | Ballantyne,2016 | 88.5% | 15.8247 | Effect stable |
| Percent TC | goldberg,2019 | 88% | 15.7482 | Effect stable |
| Percent TC | Laufs,2019 | 88.4% | 16.4691 | Effect stable |
| Percent TC | Ballantyne,2020 | 88.5% | 15.9135 | Effect stable |
| Percent TC | Ray,2019 | 88% | 17.2798 | Effect stable |
| Percent TC | Hovingh,2015 | 87.5% | 14.6514 | Effect stable |
| Percent TC | Yang,2017 | 88.4% | 15.6577 | Effect stable |
| Percent TC | Sasaki,2012 | 88.4% | 16.1091 | Effect stable |
| Percent TC | insull,2001 | 88% | 15.6120 | Effect stable |
| Percent TC | Lee,2021 | 87.2% | 14.2297 | Effect stable |
| Percent TC | Ballantyne,2018 | 88% | 15.3744 | Effect stable |
| Percent TC | Sakamoto,2017 | 88.5% | 15.8736 | Effect stable |
| Percent TC | Yamashita,2025 | 88.5% | 16.1278 | Effect stable |
| Percent TC | Gagne´,2002 | 83.3% | 11.8982 | Effect stable |
| Percent TC | ballantyne,2004_a | 82.2% | 10.8058 | Effect stable |
| Percent TC | GOLDBERG,2004 | 88.3% | 17.3790 | Effect stable |
| Percent TC | Farnier,2005 | 88.4% | 16.0942 | Effect stable |
| Percent TC | Stein,2008 | 88.5% | 16.0593 | Effect stable |
| Percent TC | Yu,2012 | 88.4% | 18.3355 | Effect stable |
| Percent TC | ballantyne,2004_b | 88.5% | 16.1605 | Effect stable |
| Percent TC | Ward,2022 | 88.4% | 15.5935 | Effect stable |
| Percent TC | Nicholls,2025 | 87.5% | 14.6514 | Effect stable |
| Percent TC | Thompson,2016 | 87.9% | 16.0126 | Effect stable |
| Percent TC | Thompson,2015 | 88% | 15.6120 | Effect stable |

Table S11.4 leave one out analysis for Absolute TC

| Outcome | Study Omitted | I^2 | tau^2 | Interpretation |
| --- | --- | --- | --- | --- |
| Absolute TC | Stein,2004 | 87.3% | 399.5026 | Effect stable |
| Absolute TC | Marrazi,2024 | 89% | 238.8442 | Effect stable |
| Absolute TC | Ray,2019 | 89% | 238.8442 | Effect stable |
| Absolute TC | Hovingh,2015 | 89% | 238.8442 | Effect stable |
| Absolute TC | Teramoto,2012 | 90.5% | 272.9728 | Effect stable |
| Absolute TC | Yang,2017 | 90.5% | 277.5942 | Effect stable |
| Absolute TC | insull,2001 | 89% | 238.8442 | Effect stable |
| Absolute TC | RUGGENENTI,2010 | 84.5% | 172.0942 | Effect stable |
| Absolute TC | Lee,2021 | 84.4% | 189.8453 | Effect stable |
| Absolute TC | Moreira,2014 | 89.1% | 240.5315 | Effect stable |
| Absolute TC | Luo,2016 | 90.5% | 264.1623 | Effect stable |
| Absolute TC | Ward,2022 | 89.8% | 230.1280 | Effect stable |
| Absolute TC | Thompson,2015 | 89% | 238.8442 | Effect stable |

Table S11.5 leave one out analysis for Percent HDL-C

| Outcome | Study Omitted | I^2 | tau^2 | Interpretation |
| --- | --- | --- | --- | --- |
| Percent HDL-C | Ballantyne,2016 | 89.2% | 21.1099 | Effect stable |
| Percent HDL-C | goldberg,2019 | 89.2% | 22.4580 | Effect stable |
| Percent HDL-C | Harada-Shiba,2024 | 89.1% | 20.7031 | Effect stable |
| Percent HDL-C | Ballantyne,2023 | 89.2% | 20.8641 | Effect stable |
| Percent HDL-C | Laufs,2019 | 89.2% | 22.3219 | Effect stable |
| Percent HDL-C | Hovingh,2015 | 60.8% | 3.9733 | Effect stable |
| Percent HDL-C | Yang,2017 | 89.2% | 21.0738 | Effect stable |
| Percent HDL-C | Nicholls,2017 | 89.2% | 21.7240 | Effect stable |
| Percent HDL-C | Sasaki,2012 | 89.2% | 21.7840 | Effect stable |
| Percent HDL-C | insull,2001 | 88.6% | 20.8040 | Effect stable |
| Percent HDL-C | Lee,2021 | 89.2% | 21.3814 | Effect stable |
| Percent HDL-C | Yamashita,2025 | 89.2% | 21.2384 | Effect stable |
| Percent HDL-C | Gagne´,2002 | 89.2% | 26.8804 | Effect stable |
| Percent HDL-C | ballantyne,2004_a | 88.6% | 21.7576 | Effect stable |
| Percent HDL-C | GOLDBERG,2004 | 88.9% | 24.4566 | Effect stable |
| Percent HDL-C | Farnier,2005 | 89.2% | 21.4396 | Effect stable |
| Percent HDL-C | Stein,2008 | 88.7% | 22.7630 | Effect stable |
| Percent HDL-C | Torimoto,2013 | 89.2% | 21.3922 | Effect stable |
| Percent HDL-C | Yu,2012 | 88.9% | 22.0098 | Effect stable |
| Percent HDL-C | ballantyne,2004_b | 89.1% | 23.0439 | Effect stable |
| Percent HDL-C | Ward,2022 | 89.2% | 21.0006 | Effect stable |
| Percent HDL-C | Nicholls,2025 | 51.6% | 2.7294 | Effect stable |
| Percent HDL-C | Thompson,2016 | 89.1% | 21.5509 | Effect stable |
| Percent HDL-C | Thompson,2015 | 88.6% | 20.8040 | Effect stable |

Table S11.6 leave one out analysis for Absolute HDL-C

| Outcome | Study Omitted | I^2 | tau^2 | Interpretation |
| --- | --- | --- | --- | --- |
| Absolute HDL-C | Stein,2004 | 2.6% | 0.2285 | Effect stable |
| Absolute HDL-C | Marrazi,2024 | 16.9% | 1.2111 | Effect stable |
| Absolute HDL-C | Hovingh,2015 | 16.9% | 1.2111 | Effect stable |
| Absolute HDL-C | Teramoto,2012 | 22.3% | 1.6208 | Effect stable |
| Absolute HDL-C | insull,2001 | 16.9% | 1.2111 | Effect stable |
| Absolute HDL-C | RUGGENENTI,2010 | 0% | 0 | Effect decreased |
| Absolute HDL-C | Lee,2021 | 23% | 1.8514 | Effect stable |
| Absolute HDL-C | Moreira,2014 | 22.9% | 1.7459 | Effect stable |
| Absolute HDL-C | Torimoto,2013 | 17.2% | 1.2008 | Effect stable |
| Absolute HDL-C | Stein,2007 | 21.6% | 1.7216 | Effect stable |
| Absolute HDL-C | Bajaj,2020 | 16.9% | 1.2111 | Effect stable |
| Absolute HDL-C | Luo,2016 | 0 | 0 | Effect decreased |
| Absolute HDL-C | Ward,2022 | 24.2% | 1.7853 | Effect stable |
| Absolute HDL-C | Efrati,2007 | 18.3% | 1.2420 | Effect stable |
| Absolute HDL-C | Moon,2023 | 24.4% | 1.8152 | Effect stable |
| Absolute HDL-C | Thompson,2015 | 16.9% | 1.2111 | Effect stable |

Table S11.7 leave one out analysis for Percent Non-HDL-C

| Outcome | Study Omitted | I^2 | tau^2 | Interpretation |
| --- | --- | --- | --- | --- |
| Percent Non-HDL-C | Ballantyne,2016 | 89.1% | 27.5770 | Effect stable |
| Percent Non-HDL-C | goldberg,2019 | 88.6% | 27.2779 | Effect stable |
| Percent Non-HDL-C | Harada-Shiba,2024 | 89.1% | 27.5751 | Effect stable |
| Percent Non-HDL-C | Ballantyne,2023 | 89% | 27.1898 | Effect stable |
| Percent Non-HDL-C | Sarraju,2025 | 89.1% | 27.5109 | Effect stable |
| Percent Non-HDL-C | Laufs,2019 | 89.1% | 29.0568 | Effect stable |
| Percent Non-HDL-C | Ballantyne,2020 | 89.1% | 27.7489 | Effect stable |
| Percent Non-HDL-C | Ray,2019 | 88.5% | 29.4035 | Effect stable |
| Percent Non-HDL-C | Nicholls,2017 | 86.8% | 26.5334 | Effect stable |
| Percent Non-HDL-C | Sasaki,2012 | 89% | 27.9282 | Effect stable |
| Percent Non-HDL-C | Lee,2021 | 89.1% | 28.0111 | Effect stable |
| Percent Non-HDL-C | Ballantyne,2018 | 88.6% | 26.6968 | Effect stable |
| Percent Non-HDL-C | Yamashita,2025 | 89.1% | 28.3493 | Effect stable |
| Percent Non-HDL-C | Gagne´,2002 | 86.8% | 26.0451 | Effect stable |
| Percent Non-HDL-C | ballantyne,2004_a | 77.7% | 13.4311 | Effect stable |
| Percent Non-HDL-C | GOLDBERG,2004 | 89.1% | 31.3421 | Effect stable |
| Percent Non-HDL-C | Farnier,2005 | 89.1% | 28.4410 | Effect stable |
| Percent Non-HDL-C | Ward,2022 | 89.1% | 27.2587 | Effect stable |
| Percent Non-HDL-C | Nicholls,2025 | 88.9% | 27.3916 | Effect stable |
| Percent Non-HDL-C | Thompson,2016 | 88.2% | 26.8180 | Effect stable |
| Percent Non-HDL-C | Thompson,2015 | 88.6% | 27.2396 | Effect stable |

Table S11.8 leave one out analysis for Absolute Non-HDL-C

| Outcome | Study Omitted | I^2 | tau^2 | Interpretation |
| --- | --- | --- | --- | --- |
| Absolute Non-HDL-C | Marrazi,2024 | 45.7% | 60.1975 | Effect stable |
| Absolute Non-HDL-C | Lee,2021 | 45.5% | 93.3865 | Effect stable |
| Absolute Non-HDL-C | Ray,2019 | 45.7% | 60.1975 | Effect stable |
| Absolute Non-HDL-C | Teramoto,2012 | 59.2% | 116.3826 | Effect stable |
| Absolute Non-HDL-C | Stein,2007 | 32.9% | 43.6594 | Effect stable |
| Absolute Non-HDL-C | Bajaj,2020 | 45.7% | 60.1975 | Effect stable |
| Absolute Non-HDL-C | Ward,2022 | 44.3% | 45.9938 | Effect stable |
| Absolute Non-HDL-C | Moon,2023 | 39.5% | 38.9826 | Effect stable |
| Absolute Non-HDL-C | Thompson,2015 | 45.7% | 60.1975 | Effect stable |

Table S11.9 leave one out analysis for Percent TG

| Outcome | Study Omitted | I^2 | tau^2 | Interpretation |
| --- | --- | --- | --- | --- |
| Percent TG | Ballantyne,2016 | 89.1% | 101.2276 | Effect stable |
| Percent TG | goldberg,2019 | 89.2% | 105.4846 | Effect stable |
| Percent TG | Harada-Shiba,2024 | 89% | 100.2475 | Effect stable |
| Percent TG | Ballantyne,2023 | 89.1% | 101.7722 | Effect stable |
| Percent TG | Laufs,2019 | 89.2% | 104.9088 | Effect stable |
| Percent TG | Hovingh,2015 | 89.2% | 102.7849 | Effect stable |
| Percent TG | Yang,2017 | 89.1% | 102.1822 | Effect stable |
| Percent TG | Nicholls,2017 | 89.1% | 113.9172 | Effect stable |
| Percent TG | insull,2001 | 88.6% | 100.2997 | Effect stable |
| Percent TG | Sasaki,2012 | 88.4% | 95.6408 | Effect stable |
| Percent TG | Lee,2021 | 89.1% | 101.2822 | Effect stable |
| Percent TG | Yamashita,2025 | 89.2% | 102.5342 | Effect stable |
| Percent TG | Gagne´,2002 | 87.6% | 101.9604 | Effect stable |
| Percent TG | ballantyne,2004_a | 89.1% | 115.6674 | Effect stable |
| Percent TG | GOLDBERG,2004 | 87.5% | 97.8030 | Effect stable |
| Percent TG | Farnier,2005 | 89.2% | 103.4643 | Effect stable |
| Percent TG | Stein,2008 | 89.1% | 119.8167 | Effect stable |
| Percent TG | Torimoto,2013 | 89% | 101.0120 | Effect stable |
| Percent TG | Yu,2012 | 58% | 21.2666 | Effect stable |
| Percent TG | ballantyne,2004_b | 88.6% | 99.5657 | Effect stable |
| Percent TG | Ward,2022 | 88.7% | 97.8548 | Effect stable |
| Percent TG | Nicholls,2025 | 89.1% | 102.4620 | Effect stable |
| Percent TG | Thompson,2016 | 89.2% | 102.1633 | Effect stable |
| Percent TG | Thompson,2015 | 88.6% | 100.2997 | Effect stable |

Table S11.10 leave one out analysis for Absolute TG

| Outcome | Study Omitted | I^2 | tau^2 | Interpretation |
| --- | --- | --- | --- | --- |
| Absolute TG | Stein,2004 | 0% | 0 | Effect stable |
| Absolute TG | Marrazi,2024 | 0% | 0 | Effect stable |
| Absolute TG | Hovingh,2015 | 0% | 0 | Effect stable |
| Absolute TG | Teramoto,2012 | 0% | 0 | Effect stable |
| Absolute TG | Yang,2017 | 0% | 0 | Effect stable |
| Absolute TG | insull,2001 | 0% | 0 | Effect stable |
| Absolute TG | RUGGENENTI,2010 | 0% | 0 | Effect stable |
| Absolute TG | Lee,2021 | 0% | 0 | Effect stable |
| Absolute TG | Moreira,2014 | 0% | 0 | Effect stable |
| Absolute TG | Yokote,2017 | 0% | 0 | Effect stable |
| Absolute TG | Torimoto,2013 | 0% | 0 | Effect stable |
| Absolute TG | Stein,2007 | 0% | 0 | Effect stable |
| Absolute TG | Bajaj,2020 | 0% | 0 | Effect stable |
| Absolute TG | Luo,2016 | 0% | 0 | Effect stable |
| Absolute TG | Ward,2022 | 0% | 0 | Effect stable |
| Absolute TG | Efrati,2007 | 0% | 0 | Effect stable |
| Absolute TG | Moon,2023 | 0% | 0 | Effect stable |
| Absolute TG | Thompson,2015 | 0% | 0 | Effect stable |

Table S11.11 leave one out analysis for Percent APO B

| Outcome | Study Omitted | I^2 | tau^2 | Interpretation |
| --- | --- | --- | --- | --- |
| Percent APO B | Ballantyne,2016 | 89.1% | 29.5872 | Effect stable |
| Percent APO B | goldberg,2019 | 88.8% | 30.2619 | Effect stable |
| Percent APO B | Harada-Shiba,2024 | 89% | 29.4801 | Effect stable |
| Percent APO B | Ballantyne,2023 | 89.1% | 29.6703 | Effect stable |
| Percent APO B | Sarraju,2025 | 89.1% | 29.8335 | Effect stable |
| Percent APO B | Laufs,2019 | 89.1% | 31.2407 | Effect stable |
| Percent APO B | Ballantyne,2020 | 89.1% | 29.6224 | Effect stable |
| Percent APO B | Ray,2019 | 88.7% | 32.6350 | Effect stable |
| Percent APO B | Hovingh,2015 | 86.5% | 23.7018 | Effect stable |
| Percent APO B | Nicholls,2017 | 87.2% | 26.3310 | Effect stable |
| Percent APO B | Sasaki,2012 | 89.1% | 30.5860 | Effect stable |
| Percent APO B | insull,2001 | 88.6% | 29.1848 | Effect stable |
| Percent APO B | Lee,2021 | 89.1% | 30.2548 | Effect stable |
| Percent APO B | Ballantyne,2018 | 88.8% | 29.3927 | Effect stable |
| Percent APO B | Yamashita,2025 | 89% | 29.9048 | Effect stable |
| Percent APO B | Gagne´,2002 | 87.5% | 29.3709 | Effect stable |
| Percent APO B | ballantyne,2004_a | 82.2% | 19.3990 | Effect stable |
| Percent APO B | GOLDBERG,2004 | 88.4% | 28.8217 | Effect stable |
| Percent APO B | Farnier,2005 | 89.1% | 30.6063 | Effect stable |
| Percent APO B | Stein,2008 | 89% | 30.7371 | Effect stable |
| Percent APO B | Nicholls,2025 | 87.4% | 26.0214 | Effect stable |
| Percent APO B | Thompson,2016 | 88.9% | 30.4920 | Effect stable |
| Percent APO B | Thompson,2015 | 88.6% | 29.1848 | Effect stable |

Table S11.12 leave one out analysis for Absolute APO B

| Outcome | Study Omitted | I^2 | tau^2 | Interpretation |
| --- | --- | --- | --- | --- |
| Absolute APO B | Stein,2004 | 66.8% | 31.3341 | Effect stable |
| Absolute APO B | Ray,2019 | 61.4% | 15.2240 | Effect stable |
| Absolute APO B | Hovingh,2015 | 61.4% | 15.2240 | Effect stable |
| Absolute APO B | Sasaki,2012 | 65.9% | 25.5268 | Effect stable |
| Absolute APO B | insull,2001 | 61.4% | 15.2240 | Effect stable |
| Absolute APO B | Sakamoto,2017 | 65.3% | 17.5298 | Effect stable |
| Absolute APO B | RUGGENENTI,2010 | 56.9% | 12.0869 | Effect stable |
| Absolute APO B | Lee,2021 | 66% | 18.3619 | Effect stable |
| Absolute APO B | Moreira,2014 | 52.2% | 9.6206 | Effect stable |
| Absolute APO B | Stein,2007 | 45.2% | 7.2128 | Effect stable |
| Absolute APO B | Moon,2023 | 66.7% | 16.6895 | Effect stable |
| Absolute APO B | Thompson,2015 | 61.4% | 15.2240 | Effect stable |

Table S11.13 leave one out analysis for Percent APO A

| Outcome | Study Omitted | I^2 | tau^2 | Interpretation |
| --- | --- | --- | --- | --- |
| Percent APO A | Ballantyne,2016 | 92.8% | 23.1739 | Effect stable |
| Percent APO A | Hovingh,2015 | 81.8% | 8.7180 | Effect stable |
| Percent APO A | Nicholls,2017 | 92.1% | 26.3497 | Effect stable |
| Percent APO A | Sasaki,2012 | 92.9% | 24.6088 | Effect stable |
| Percent APO A | insull,2001 | 92.1% | 22.6576 | Effect stable |
| Percent APO A | Lee,2021 | 92.8% | 23.9516 | Effect stable |
| Percent APO A | Gagne´,2002 | 92.9% | 28.9834 | Effect stable |
| Percent APO A | ballantyne,2004_a | 90.2% | 19.9408 | Effect stable |
| Percent APO A | GOLDBERG,2004 | 92.5% | 25.4817 | Effect stable |
| Percent APO A | Farnier,2005 | 92.9% | 23.8346 | Effect stable |
| Percent APO A | Stein,2008 | 92.6% | 23.5204 | Effect stable |
| Percent APO A | insull,2001 | 88.6% | 29.1848 | Effect stable |
| Percent APO A | Nicholls,2025 | 81.8% | 8.7180 | Effect stable |
| Percent APO A | Thompson,2016 | 92.8% | 23.6537 | Effect stable |
| Percent APO A | Thompson,2015 | 92.1% | 22.6576 | Effect stable |

Table S11.14 Leave one out analysis for Serious adverse events

| Outcome | Study Omitted | I^2 | tau^2 | Interpretation |
| --- | --- | --- | --- | --- |
| Serious adverse events | Bajaj,2020 | 0% | 0 | Effect stable |
| Serious adverse events | Ballantyne,2020 | 0% | 0 | Effect stable |
| Serious adverse events | Ballantyne,2016 | 0% | 0 | Effect stable |
| Serious adverse events | Ballantyne,2018 | 0% | 0 | Effect stable |
| Serious adverse events | ballantyne,2004_b | 0% | 0 | Effect stable |
| Serious adverse events | Farnier,2005 | 0% | 0 | Effect stable |
| Serious adverse events | Gagne´,2002 | 0% | 0 | Effect stable |
| Serious adverse events | goldberg,2019 | 0% | 0 | Effect stable |
| Serious adverse events | GOLDBERG,2004 | 0% | 0 | Effect stable |
| Serious adverse events | Harada-Shiba,2024 | 0% | 0 | Effect stable |
| Serious adverse events | insull,2001 | 0% | 0 | Effect stable |
| Serious adverse events | Laufs,2019 | 0% | 0 | Effect stable |
| Serious adverse events | Lee,2021 | 0% | 0 | Effect stable |
| Serious adverse events | Moon,2023 | 0% | 0 | Effect stable |
| Serious adverse events | Nicholls,2025 | 0% | 0 | Effect stable |
| Serious adverse events | Qian,2022_a | 0% | 0 | Effect stable |
| Serious adverse events | Qian,2022_b | 0% | 0 | Effect stable |
| Serious adverse events | Ray,2019 | 0% | 0 | Effect stable |
| Serious adverse events | Sarraju,2025 | 0% | 0 | Effect stable |
| Serious adverse events | Sasaki,2012 | 0% | 0 | Effect stable |
| Serious adverse events | Stein,2004 | 0% | 0 | Effect stable |
| Serious adverse events | Stein,2008 | 0% | 0 | Effect stable |
| Serious adverse events | Stein,2007 | 0% | 0 | Effect stable |
| Serious adverse events | Thompson,2016 | 0% | 0 | Effect stable |
| Serious adverse events | Thompson,2015 | 0% | 0 | Effect stable |
| Serious adverse events | Yamashita,2025 | 0% | 0 | Effect stable |
| Serious adverse events | Yang,2017 | 0% | 0 | Effect stable |
| Serious adverse events | Yu,2012 | 0% | 0 | Effect stable |

Table S11.15 Leave one out analysis for Treatment discontinuation

| Outcome | Study Omitted | I^2 | tau^2 | Interpretation |
| --- | --- | --- | --- | --- |
| Treatment discontinuation | Bajaj,2020 | 0% | 0 | Effect stable |
| Treatment discontinuation | ballantyne,2004_b | 0% | 0 | Effect stable |
| Treatment discontinuation | Ballantyne,2016 | 0% | 0 | Effect stable |
| Treatment discontinuation | Ballantyne,2018 | 0% | 0 | Effect stable |
| Treatment discontinuation | Ballantyne,2020 | 0% | 0 | Effect stable |
| Treatment discontinuation | Ballantyne,2023 | 0% | 0 | Effect stable |
| Treatment discontinuation | Farnier,2005 | 0% | 0 | Effect stable |
| Treatment discontinuation | Gagne´,2002 | 0% | 0 | Effect stable |
| Treatment discontinuation | goldberg,2019 | 0% | 0 | Effect stable |
| Treatment discontinuation | GOLDBERG,2004 | 0% | 0 | Effect stable |
| Treatment discontinuation | Harada-Shiba,2024 | 0% | 0 | Effect stable |
| Treatment discontinuation | Hovingh,2015 | 0% | 0 | Effect stable |
| Treatment discontinuation | insull,2001 | 0% | 0 | Effect stable |
| Treatment discontinuation | Laufs,2019 | 0% | 0 | Effect stable |
| Treatment discontinuation | Lee,2021 | 0% | 0 | Effect stable |
| Treatment discontinuation | Moon,2023 | 0% | 0 | Effect stable |
| Treatment discontinuation | Nicholls,2017 | 0% | 0 | Effect stable |
| Treatment discontinuation | Nicholls,2025 | 0% | 0 | Effect stable |
| Treatment discontinuation | Qian,2022_a | 0% | 0 | Effect stable |
| Treatment discontinuation | Ray,2019 | 0% | 0 | Effect stable |
| Treatment discontinuation | Sakamoto,2017 | 0% | 0 | Effect stable |
| Treatment discontinuation | Sarraju,2025 | 0% | 0 | Effect stable |
| Treatment discontinuation | Sasaki,2012 | 0% | 0 | Effect stable |
| Treatment discontinuation | Stein,2004 | 0% | 0 | Effect stable |
| Treatment discontinuation | Stein,2008 | 0% | 0 | Effect stable |
| Treatment discontinuation | Thompson,2016 | 0% | 0 | Effect stable |
| Treatment discontinuation | Thompson,2015 | 0% | 0 | Effect stable |
| Treatment discontinuation | Yamashita,2025 | 0% | 0 | Effect stable |
| Treatment discontinuation | Yang,2017 | 0% | 0 | Effect stable |

Table S11.16 Leave one out analysis for Myalgia

| Outcome | Study Omitted | I^2 | tau^2 | Interpretation |
| --- | --- | --- | --- | --- |
| Myalgia | Bajaj,2020 | 0% | 0 | Effect stable |
| Myalgia | Ballantyne,2023 | 0% | 0 | Effect stable |
| Myalgia | Ballantyne,2020 | 0% | 0 | Effect stable |
| Myalgia | Ballantyne,2016 | 0% | 0 | Effect stable |
| Myalgia | Ballantyne,2018 | 0% | 0 | Effect stable |
| Myalgia | ballantyne,2004_b | 0% | 0 | Effect stable |
| Myalgia | Farnier,2005 | 0% | 0 | Effect stable |
| Myalgia | goldberg,2019 | 0% | 0 | Effect stable |
| Myalgia | Harada-Shiba,2024 | 0% | 0 | Effect stable |
| Myalgia | Hovingh,2015 | 0% | 0 | Effect stable |
| Myalgia | Laufs,2019 | 0% | 0 | Effect stable |
| Myalgia | Lee,2021 | 0% | 0 | Effect stable |
| Myalgia | Qian,2022_a | 0% | 0 | Effect stable |
| Myalgia | Qian,2022_b | 0% | 0 | Effect stable |
| Myalgia | Ray,2019 | 0% | 0 | Effect stable |
| Myalgia | Sakamoto,2017 | 0% | 0 | Effect stable |
| Myalgia | Stein,2004 | 0% | 0 | Effect stable |
| Myalgia | Stein,2008 | 0% | 0 | Effect stable |
| Myalgia | Thompson,2016 | 0% | 0 | Effect stable |
| Myalgia | Thompson,2015 | 0% | 0 | Effect stable |
| Myalgia | Yamashita,2025 | 0% | 0 | Effect stable |

Table S11.17 Leave one out analysis for Headache

| Outcome | Study Omitted | I^2 | tau^2 | Interpretation |
| --- | --- | --- | --- | --- |
| Headache | Bajaj,2020 | 0% | 0 | Effect stable |
| Headache | Ballantyne,2016 | 0% | 0 | Effect stable |
| Headache | Ballantyne,2020 | 0% | 0 | Effect stable |
| Headache | Ballantyne,2023 | 0% | 0 | Effect stable |
| Headache | Hovingh,2015 | 0% | 0 | Effect stable |
| Headache | insull,2001 | 0% | 0 | Effect stable |
| Headache | Lee,2021 | 0% | 0 | Effect stable |
| Headache | Nicholls,2025 | 0% | 0 | Effect stable |
| Headache | Sarraju,2025 | 0% | 0 | Effect stable |
| Headache | Stein,2004 | 0% | 0 | Effect stable |
| Headache | Thompson,2016 | 0% | 0 | Effect stable |
| Headache | Thompson,2015 | 0% | 0 | Effect stable |
| Headache | Yu,2012 | 0% | 0 | Effect stable |

Table S11.18 Leave one out analysis for Elevated liver enzymes

| Outcome | Study Omitted | I^2 | tau^2 | Interpretation |
| --- | --- | --- | --- | --- |
| Elevated liver enzymes | Ballantyne,2020 | 0% | 0 | Effect stable |
| Elevated liver enzymes | Ballantyne,2016 | 0% | 0 | Effect stable |
| Elevated liver enzymes | Ballantyne,2018 | 0% | 0 | Effect stable |
| Elevated liver enzymes | Ballantyne,2023 | 0% | 0 | Effect stable |
| Elevated liver enzymes | Farnier,2005 | 0% | 0 | Effect stable |
| Elevated liver enzymes | Gagne´,2002 | 0% | 0 | Effect stable |
| Elevated liver enzymes | goldberg,2019 | 0% | 0 | Effect stable |
| Elevated liver enzymes | GOLDBERG,2004 | 0% | 0 | Effect stable |
| Elevated liver enzymes | Lee,2021 | 0% | 0 | Effect stable |
| Elevated liver enzymes | Nicholls,2017 | 0% | 0 | Effect stable |
| Elevated liver enzymes | Nicholls,2025 | 0% | 0 | Effect stable |
| Elevated liver enzymes | Qian,2022_a | 0% | 0 | Effect stable |
| Elevated liver enzymes | Qian,2022_b | 0% | 0 | Effect stable |
| Elevated liver enzymes | Ray,2019 | 0% | 0 | Effect stable |
| Elevated liver enzymes | Sakamoto,2017 | 0% | 0 | Effect stable |
| Elevated liver enzymes | Sarraju,2025 | 0% | 0 | Effect stable |
| Elevated liver enzymes | Stein,2004 | 0% | 0 | Effect stable |
| Elevated liver enzymes | Stein,2008 | 0% | 0 | Effect stable |
| Elevated liver enzymes | Thompson,2016 | 0% | 0 | Effect stable |
| Elevated liver enzymes | Yamashita,2025 | 0% | 0 | Effect stable |
| Elevated liver enzymes | Yang,2017 | 0% | 0 | Effect stable |

**Supplement S10: PRISMA check list**

| **Section and Topic** | **Item #** | **Checklist item** | **Location where item is reported** |
| --- | --- | --- | --- |
| **TITLE** | | |  |
| Title | 1 | Identify the report as a systematic review. | 1 |
| **ABSTRACT** | | |  |
| Abstract | 2 | See the PRISMA 2020 for Abstracts checklist. | 2 |
| **INTRODUCTION** | | |  |
| Rationale | 3 | Describe the rationale for the review in the context of existing knowledge. | 3 |
| Objectives | 4 | Provide an explicit statement of the objective(s) or question(s) the review addresses. | 3 |
| **METHODS** | | |  |
| Eligibility criteria | 5 | Specify the inclusion and exclusion criteria for the review and how studies were grouped for the syntheses. | 4 |
| Information sources | 6 | Specify all databases, registers, websites, organisations, reference lists and other sources searched or consulted to identify studies. Specify the date when each source was last searched or consulted. | 4 |
| Search strategy | 7 | Present the full search strategies for all databases, registers and websites, including any filters and limits used. | 4 |
| Selection process | 8 | Specify the methods used to decide whether a study met the inclusion criteria of the review, including how many reviewers screened each record and each report retrieved, whether they worked independently, and if applicable, details of automation tools used in the process. | 5,6 |
| Data collection process | 9 | Specify the methods used to collect data from reports, including how many reviewers collected data from each report, whether they worked independently, any processes for obtaining or confirming data from study investigators, and if applicable, details of automation tools used in the process. | 5,6 |
| Data items | 10a | List and define all outcomes for which data were sought. Specify whether all results that were compatible with each outcome domain in each study were sought (e.g. for all measures, time points, analyses), and if not, the methods used to decide which results to collect. | 5 |
|  | 10b | List and define all other variables for which data were sought (e.g. participant and intervention characteristics, funding sources). Describe any assumptions made about any missing or unclear information. | 5 |
| Study risk of bias assessment | 11 | Specify the methods used to assess risk of bias in the included studies, including details of the tool(s) used, how many reviewers assessed each study and whether they worked independently, and if applicable, details of automation tools used in the process. | 5 |
| Effect measures | 12 | Specify for each outcome the effect measure(s) (e.g. risk ratio, mean difference) used in the synthesis or presentation of results. | 5 |
| Synthesis methods | 13a | Describe the processes used to decide which studies were eligible for each synthesis (e.g. tabulating the study intervention characteristics and comparing against the planned groups for each synthesis (item #5)). | 5 |
|  | 13b | Describe any methods required to prepare the data for presentation or synthesis, such as handling of missing summary statistics, or data conversions. | 5 |
|  | 13c | Describe any methods used to tabulate or visually display results of individual studies and syntheses. | 5 |
|  | 13d | Describe any methods used to synthesize results and provide a rationale for the choice(s). If meta-analysis was performed, describe the model(s), method(s) to identify the presence and extent of statistical heterogeneity, and software package(s) used. | 5 |
|  | 13e | Describe any methods used to explore possible causes of heterogeneity among study results (e.g. subgroup analysis, meta-regression). | 5 |
|  | 13f | Describe any sensitivity analyses conducted to assess robustness of the synthesized results. | 5 |
| Reporting bias assessment | 14 | Describe any methods used to assess risk of bias due to missing results in a synthesis (arising from reporting biases). | 6 |
| Certainty assessment | 15 | Describe any methods used to assess certainty (or confidence) in the body of evidence for an outcome. | 6 |
| **RESULTS** | | |  |
| Study selection | 16a | Describe the results of the search and selection process, from the number of records identified in the search to the number of studies included in the review, ideally using a flow diagram. | 4, supplement, figure 1 |
|  | 16b | Cite studies that might appear to meet the inclusion criteria, but which were excluded, and explain why they were excluded. | 4 |
| Study characteristics | 17 | Cite each included study and present its characteristics. | 4, supplement |
| Risk of bias in studies | 18 | Present assessments of risk of bias for each included study. | 6, supplement |
| Results of individual studies | 19 | For all outcomes, present, for each study: (a) summary statistics for each group (where appropriate) and (b) an effect estimate and its precision (e.g. confidence/credible interval), ideally using structured tables or plots. | 7-11 |
| Results of syntheses | 20a | For each synthesis, briefly summarise the characteristics and risk of bias among contributing studies. | 7-11 |
|  | 20b | Present results of all statistical syntheses conducted. If meta-analysis was done, present for each the summary estimate and its precision (e.g. confidence/credible interval) and measures of statistical heterogeneity. If comparing groups, describe the direction of the effect. | 7-11 |
|  | 20c | Present results of all investigations of possible causes of heterogeneity among study results. | 7-11 |
|  | 20d | Present results of all sensitivity analyses conducted to assess the robustness of the synthesized results. | 7-11, supplement |
| Reporting biases | 21 | Present assessments of risk of bias due to missing results (arising from reporting biases) for each synthesis assessed. | 6, supplement |
| Certainty of evidence | 22 | Present assessments of certainty (or confidence) in the body of evidence for each outcome assessed. | 6, supplement |
| **DISCUSSION** | | |  |
| Discussion | 23a | Provide a general interpretation of the results in the context of other evidence. |  |
|  | 23b | Discuss any limitations of the evidence included in the review. | 12-13 |
|  | 23c | Discuss any limitations of the review processes used. | 12-13 |
|  | 23d | Discuss implications of the results for practice, policy, and future research. | 12-13 |
| **OTHER INFORMATION** | | |  |
| Registration and protocol | 24a | Provide registration information for the review, including register name and registration number, or state that the review was not registered. | 4 |
|  | 24b | Indicate where the review protocol can be accessed, or state that a protocol was not prepared. | 4 |
|  | 24c | Describe and explain any amendments to information provided at registration or in the protocol. | 4 |
| Support | 25 | Describe sources of financial or non-financial support for the review, and the role of the funders or sponsors in the review. | 14 |
| Competing interests | 26 | Declare any competing interests of review authors. | 14 |
| Availability of data, code and other materials | 27 | Report which of the following are publicly available and where they can be found: template data collection forms; data extracted from included studies; data used for all analyses; analytic code; any other materials used in the review. | 14 |

**References:**

1. Stein E, Stender S, Mata P, et al. Achieving lipoprotein goals in patients at high risk with severe hypercholesterolemia: Efficacy and safety of ezetimibe co-administered with atorvastatin. *Am Heart J*. 2004;148(3):447-455. doi:10.1016/j.ahj.2004.03.052

2.	Marazzi G, Caminiti G, Perrone MA, et al. Addition of Bempedoic Acid to Statin-Ezetimibe versus Statin Titration in Patients with High Cardiovascular Risk: A Single-Centre Prospective Study. *J Cardiovasc Dev Dis*. 2024;11(9). doi:10.3390/JCDD11090286

3.	Goldberg AC, Leiter LA, Stroes ESG, et al. Effect of Bempedoic Acid vs Placebo Added to Maximally Tolerated Statins on Low-Density Lipoprotein Cholesterol in Patients at High Risk for Cardiovascular Disease: The CLEAR Wisdom Randomized Clinical Trial. *JAMA*. 2019;322(18):1780-1788. doi:10.1001/JAMA.2019.16585

4.	Harada-Shiba M, Davdison MH, Ditmarsch M, et al. Obicetrapib as an Adjunct to Stable Statin Therapy in Japanese Subjects: Results from a Randomized Phase 2 Trial. *J Atheroscler Thromb*. 2024;31(10):1386-1397. doi:10.5551/JAT.64828

5.	Ballantyne CM, Ditmarsch M, Kastelein JJ, et al. Obicetrapib plus ezetimibe as an adjunct to high-intensity statin therapy: A randomized phase 2 trial. *J Clin Lipidol*. 2023;17(4):491-503. doi:10.1016/j.jacl.2023.05.098

6.	Sarraju A, Brennan D, Hayden K, et al. Fixed-dose combination of obicetrapib and ezetimibe for LDL cholesterol reduction (TANDEM): a phase 3, randomised, double-blind, placebo-controlled trial. *Lancet*. 2025;405(10491):1757-1768. doi:10.1016/S0140-6736(25)00721-4

7.	Laufs U, Banach M, Mancini GBJ, et al. Efficacy and Safety of Bempedoic Acid in Patients With Hypercholesterolemia and Statin Intolerance. *J Am Heart Assoc*. 2019;8(7). doi:10.1161/JAHA.118.011662

8.	Ballantyne CM, Laufs U, Ray KK, et al. Bempedoic acid plus ezetimibe fixed-dose combination in patients with hypercholesterolemia and high CVD risk treated with maximally tolerated statin therapy. *Eur J Prev Cardiol*. 2020;27(6):593-603. doi:10.1177/2047487319864671

9.	Ballantyne CM, McKenney JM, MacDougall DE, et al. Effect of ETC-1002 on Serum Low-Density Lipoprotein Cholesterol in Hypercholesterolemic Patients Receiving Statin Therapy. *Am J Cardiol*. 2016;117(12):1928-1933. doi:10.1016/j.amjcard.2016.03.043

10.	Ray KK, Bays HE, Catapano AL, et al. Safety and Efficacy of Bempedoic Acid to Reduce LDL Cholesterol. *N Engl J Med*. 2019;380(11):1022-1032. doi:10.1056/NEJMOA1803917

11.	Hovingh GK, Kastelein JJP, Van Deventer SJH, et al. Cholesterol ester transfer protein inhibition by TA-8995 in patients with mild dyslipidaemia (TULIP): A randomised, double-blind, placebo-controlled phase 2 trial. *Lancet*. 2015;386(9992):452-460. doi:10.1016/S0140-6736(15)60158-1

12.	Teramoto T, Sawada T, Iwamoto K, Daida H. Clinical Efficacy and Tolerability of Ezetimibe in Combination With Atorvastatin in Japanese Patients With Hypercholesterolemia-Ezetimibe Phase IV Randomized Controlled Trial in Patients With Hypercholesterolemia. *Curr Ther Res - Clin Exp*. 2012;73(1-2):16-40. doi:10.1016/j.curtheres.2012.02.002

13.	Yang YJ, Lee SH, Kim BS, et al. Combination Therapy of Rosuvastatin and Ezetimibe in Patients with High Cardiovascular Risk. *Clin Ther*. 2017;39(1):107-117. doi:10.1016/j.clinthera.2016.11.014

14.	Nicholls SJ, Ray KK, Ballantyne CM, et al. Comparative effects of cholesteryl ester transfer protein inhibition, statin or ezetimibe on lipid factors: The ACCENTUATE trial. *Atherosclerosis*. 2017;261:12-18. doi:10.1016/j.atherosclerosis.2017.04.008

15.	Sasaki J, Otonari T, Sawayama Y, et al. Double-dose pravastatin versus add-on ezetimibe with low-dose pravastatin - effects on LDL cholesterol, cholesterol absorption, and cholesterol synthesis in Japanese patients with hypercholesterolemia (PEAS study). *J Atheroscler Thromb*. 2012;19(5):485-493. doi:10.5551/JAT.12013

16.	Insull W, Toth P, Mullican W, et al. Effectiveness of colesevelam hydrochloride in decreasing LDL cholesterol in patients with primary hypercholesterolemia: a 24-week randomized controlled trial. *Mayo Clin Proc*. 2001;76(10):971-982. doi:10.4065/76.10.971

17.	Sakamoto K, Kawamura M, Watanabe T, et al. Effect of ezetimibe add-on therapy over 52 weeks extension analysis of prospective randomized trial (RESEARCH study) in type 2 diabetes subjects. *Lipids Health Dis*. 2017;16(1). doi:10.1186/S12944-017-0508-4

18.	Ruggenenti P, Cattaneo D, Rota S, et al. Effects of combined ezetimibe and simvastatin therapy as compared with simvastatin alone in patients with type 2 diabetes: a prospective randomized double-blind clinical trial. *Diabetes Care*. 2010;33(9):1954-1956. doi:10.2337/DC10-0320

19.	Lee SA, Kim W, Hong TJ, et al. Effects of Fixed-dose Combination of Low-intensity Rosuvastatin and Ezetimibe Versus Moderate-intensity Rosuvastatin Monotherapy on Lipid Profiles in Patients With Hypercholesterolemia: A Randomized, Double-blind, Multicenter, Phase III Study. *Clin Ther*. 2021;43(9):1573-1589. doi:10.1016/j.clinthera.2021.07.016

20.	Moreira FT, Ramos SC, Monteiro AM, et al. Effects of two lipid lowering therapies on immune responses in hyperlipidemic subjects. *Life Sci*. 2014;98(2):83-87. doi:10.1016/j.lfs.2014.01.001

21.	Ballantyne CM, Banach M, Mancini GBJ, et al. Efficacy and safety of bempedoic acid added to ezetimibe in statin-intolerant patients with hypercholesterolemia: A randomized, placebo-controlled study. *Atherosclerosis*. 2018;277:195-203. doi:10.1016/j.atherosclerosis.2018.06.002

22.	Yamashita S, Kiyosue A, Fujita H, Yokota D, Nakamura Y, Yasuda S. Efficacy and Safety of Bempedoic Acid in Japanese Patients With Hypercholesterolemia - A Randomized, Double-Blind, Placebo-Controlled Phase 3 Study (the CLEAR-J Trial). *Circ J*. 2025;89(8):1256-1265. doi:10.1253/CIRCJ.CJ-25-0089

23.	Yokote K, Kanada S, Matsuoka O, et al. Efficacy and Safety of Bococizumab (RN316/PF-04950615), a Monoclonal Antibody Against Proprotein Convertase Subtilisin/Kexin Type 9, in Hypercholesterolemic Japanese Subjects Receiving a Stable Dose of Atorvastatin or Treatment-Naive - Results From a Ran…. *Circ J*. 2017;81(10):1496-1505. doi:10.1253/CIRCJ.CJ-16-1310

24.	Gagné C, Bays HE, Weiss SR, et al. Efficacy and safety of ezetimibe added to ongoing statin therapy for treatment of patients with primary hypercholesterolemia. *Am J Cardiol*. 2002;90(10):1084-1091. doi:10.1016/S0002-9149(02)02774-1

25.	Ballantyne CM, Blazing MA, King TR, Brady WE, Palmisano J. Efficacy and safety of ezetimibe co-administered with simvastatin compared with atorvastatin in adults with hypercholesterolemia. *Am J Cardiol*. 2004;93(12):1487-1494. doi:10.1016/j.amjcard.2004.02.060

26.	Goldberg AC, Sapre A, Liu J, Capece R, Mitchel YB. Efficacy and safety of ezetimibe coadministered with simvastatin in patients with primary hypercholesterolemia: a randomized, double-blind, placebo-controlled trial. *Mayo Clin Proc*. 2004;79(5):620-629. doi:10.4065/79.5.620

27.	Farnier M, Freeman MW, Macdonell G, et al. Efficacy and safety of the coadministration of ezetimibe with fenofibrate in patients with mixed hyperlipidaemia. *Eur Heart J*. 2005;26(9):897-905. doi:10.1093/EURHEARTJ/EHI231

28.	Qian J, Li Z, Zhang X, et al. Efficacy and Tolerability of Ezetimibe/Atorvastatin Fixed-dose Combination Versus Atorvastatin Monotherapy in Hypercholesterolemia: A Phase III, Randomized, Active-controlled Study in Chinese Patients. *Clin Ther*. 2022;44(10):1282-1296. doi:10.1016/j.clinthera.2022.08.013

29.	Stein EA, Ballantyne CM, Windler E, et al. Efficacy and Tolerability of Fluvastatin XL 80 mg Alone, Ezetimibe Alone, and the Combination of Fluvastatin XL 80 mg With Ezetimibe in Patients With a History of Muscle-Related Side Effects With Other Statins. *Am J Cardiol*. 2008;101(4):490-496. doi:10.1016/j.amjcard.2007.09.099

30.	Torimoto K, Okada Y, Mori H, et al. Efficacy of combination of Ezetimibe 10 mg and rosuvastatin 2.5 mg versus rosuvastatin 5 mg monotherapy for hypercholesterolemia in patients with type 2 diabetes. *Lipids Health Dis*. 2013;12(1). doi:10.1186/1476-511X-12-137

31.	Ballantyne CM, Hoogeveen RC, Raya JL, Cain VA, Palmer MK, Karlson BW. Efficacy, safety and effect on biomarkers related to cholesterol and lipoprotein metabolism of rosuvastatin 10 or 20 mg plus ezetimibe 10 mg vs. simvastatin 40 or 80 mg plus ezetimibe 10 mg in high-risk patients: Results of the GRAVITY randomized study. *Atherosclerosis*. 2014;232(1):86-93. doi:10.1016/J.ATHEROSCLEROSIS.2013.10.022

32.	Yu CC, Lai W Ter, Shih KC, et al. Efficacy, safety and tolerability of ongoing statin plus ezetimibe versus doubling the ongoing statin dose in hypercholesterolemic Taiwanese patients: an open-label, randomized clinical trial. *BMC Res Notes*. 2012;5. doi:10.1186/1756-0500-5-251

33.	Stein EA, Ose L, Retterstøl K, et al. Further reduction of low-density lipoprotein cholesterol and C-reactive protein with the addition of ezetimibe to maximum-dose rosuvastatin in patients with severe hypercholesterolemia. *J Clin Lipidol*. 2007;1(4):280-286. doi:10.1016/J.JACL.2007.07.003

34.	Bajaj HS, Brown RE, Jiandani D, et al. Goal achievement of HbA1c and LDL-cholesterol in a randomized trial comparing colesevelam with ezetimibe: GOAL-RCT. *Diabetes Obes Metab*. 2020;22(10):1722-1728. doi:10.1111/DOM.14084

35.	Luo P, Wang L, Zhu H, Du S, Wang G, Ding S. Impact of Atorvastatin Combined with Ezetimibe for the Treatment of Carotid Atherosclerosis in Patients with Coronary Heart Disease. *Acta Cardiol Sin*. 2016;32(5):578-585. doi:10.6515/ACS20151013H

36.	Ballantyne CM, Lipka LJ, Sager PT, et al. Long-term safety and tolerability profile of ezetimibe and atorvastatin coadministration therapy in patients with primary hypercholesterolaemia. *Int J Clin Pract*. 2004;58(7):653-658. doi:10.1111/J.1368-5031.2004.00278.X

37.	Ward NC, Reid CM, Watts GF. Low-density lipoprotein-cholesterol lowering effect of a nutraceutical regimen with or without ezetimibe in hypercholesterolaemic patients with statin intolerance. *Front Cardiovasc Med*. 2022;9. doi:10.3389/FCVM.2022.1060252

38.	Nicholls SJ, Nelson AJ, Ditmarsch M, et al. Safety and Efficacy of Obicetrapib in Patients at High Cardiovascular Risk. *N Engl J Med*. 2025;393(1):51-61. doi:10.1056/NEJMOA2415820

39.	Efrati S, Averbukh M, Dishy V, Faygenzo M, Friedensohn L, Golik A. The effect of simvastatin, ezetimibe and their combination on the lipid profile, arterial stiffness and inflammatory markers. *Eur J Clin Pharmacol*. 2007;63(2):113-121. doi:10.1007/S00228-006-0238-4

40.	Moon JS, Park IR, Kim SS, et al. The Efficacy and Safety of Moderate-Intensity Rosuvastatin with Ezetimibe versus High-Intensity Rosuvastatin in High Atherosclerotic Cardiovascular Disease Risk Patients with Type 2 Diabetes Mellitus: A Randomized, Multicenter, Open, Parallel, Phase 4 St…. *Diabetes Metab J*. 2023;47(6):818-825. doi:10.4093/DMJ.2023.0171

41.	Strony J, Hoffman R, Hanson M, Veltri E. Tolerability and effects on lipids of ezetimibe coadministered with pravastatin or simvastatin for twelve months: results from two open-label extension studies in hypercholesterolemic patients. *Clin Ther*. 2008;30(12):2280-2297. doi:10.1016/J.CLINTHERA.2008.12.008

42.	Thompson PD, MacDougall DE, Newton RS, et al. Treatment with ETC-1002 alone and in combination with ezetimibe lowers LDL cholesterol in hypercholesterolemic patients with or without statin intolerance. *J Clin Lipidol*. 2016;10(3):556-567. doi:10.1016/j.jacl.2015.12.025

43.	Thompson PD, Rubino J, Janik MJ, et al. Use of ETC-1002 to treat hypercholesterolemia in patients with statin intolerance. *J Clin Lipidol*. 2015;9(3):295-304. doi:10.1016/j.jacl.2015.03.003
